# Within-family effect of ancestry on complex traits in a Mexican population

**DOI:** 10.1038/s41586-026-11039-9

**Published:** 2026-09-09

**Authors:** Siqi Wang, Jaime Berumen, Alejandra Vergara-Lope, Paulina Baca, Elizabeth Barrera, Fernando Rivas, Diego Aguilar-Ramirez, Rory Collins, Jonathan R. Emberson, Michael Hill, Michael E. Goddard, Loic Yengo, Alexander Strudwick Young, Jesus Alegre-Díaz, Pablo Kuri-Morales, Roberto Tapia-Conyer, Jason M. Torres, Peter M. Visscher

**Affiliations:** 1 Nuffield Department of Population Health, University of Oxford, Oxford, UK; 2 Experimental Research Unit from the Faculty of Medicine, National Autonomous University of Mexico, Mexico City, Mexico; 3 Centre for AgriBioscience, Agriculture Victoria, Melbourne, Victoria Australia; 4 Faculty of Veterinary and Agricultural Sciences, University of Melbourne, Parkville, Victoria Australia; 5 Institute for Molecular Bioscience, The University of Queensland, Brisbane, Queensland Australia; 6 Human Genetics Department, UCLA David Geffen School of Medicine, Los Angeles, CA USA; 7 Tecnológico de Monterrey, Proyecto OriGen, Monterrey, Mexico; 8 Faculty of Medicine, National Autonomous University of Mexico, Mexico City, Mexico

**Keywords:** Genetics research, Epidemiology, Heritable quantitative trait, Genetic association study, Population genetics

## Abstract

Human populations differ in disease prevalence and phenotypes, but the extent to which differences are caused by genetic factors is unknown for most complex traits. Comparing phenotypic means across populations is confounded by environmental differences and using polygenic predictors can lead to biased inference^1,2^. Family-based analyses of people of genetically admixed ancestry enable estimation of ancestry effects unconfounded by ancestry–environment correlations. Here we leverage genetic data from admixed adults in the Mexico City Prospective Study^3,4^ to estimate within-family ancestry effects^5^. We assessed genetic ancestry and 15 complex traits in 52,583 unrelated people and 39,714 relatives from 17,627 families. At the population level, relative to European ancestry, the effect of Indigenous American ancestry was −1.98 s.d. (*P* < 2 × 10^−16^) for height and a natural log odds ratio of 1.73 (95% confidence interval, 1.54–1.92) for type 2 diabetes. Within families, the effect of Indigenous American ancestry was −1.51 s.d. (*P* = 10^−8^) for height and natural log odds ratio of 5.13 (95% confidence interval, 2.48–7.78) for type 2 diabetes. These effects are supported by between-ancestry differences in trait-increasing allele counts and evidence of selection at trait-associated loci. We found no within-family ancestry effect on educational attainment or other traits despite significant associations at the population level, implying environmental causes or confounding. Overall, this study provides an experimental design to study between-ancestry genetic effects and identifies significant ancestry differences for height, type 2 diabetes and metabolic traits in a genetically diverse population from Mexico City.

## Abstract (web summary)

This study uses a within-family design to identify significant ancestry differences in complex traits such as height and type 2 diabetes in a genetically diverse population from Mexico City.

## Main

Natural selection and genetic drift produce differences in allele frequencies among populations, which could differentially shape phenotype distributions^6^. In humans, some phenotypic differences among genetic ancestry groups are known to have a genetic basis, including pigmentation^7^, drug metabolism^8^ and the prevalence of single-gene disorders^9,10^. (Throughout this paper, we use the term ‘genetic ancestry’ to refer to inherited variation from ancestral populations that differ in allele frequencies across the genome and that can be inferred from genetic data. We acknowledge that there is no consensus on terminology^11,12^ and that human populations are not genetically discrete.) Whether inherited genetic differences also contribute to population differences in complex traits such as height and common diseases such as type 2 diabetes (T2D) remains poorly understood. Here we combine large-scale genomic data with a within-family study design to address this question.

In admixed populations, the genomes of individual people vary in their proportions of different founder ancestries, and these proportions can be estimated with genetic markers^13^. Ancestry proportions can correlate with trait values at the population level^14–16^, but may also track environmental effects, confounding attempts to separate genetic from non-genetic contributions to trait variation. For example, in Mexico, obesity^17^ and T2D are highly prevalent and associated with ancestry^18^, but the underlying mechanisms remain unclear.

In gene–trait association studies, the gold standard for inferring causal effects of genotypes on phenotypes is to perform a within-family association analysis, which conditions on parental genotypes^19,20^. Within-family genetic variation is due to random segregation of parental alleles during meiosis, therefore associations between within-family genotype variation and phenotype are expected to be due only to the causal effects of inherited alleles^21–23^. Here ancestry proportions are first inferred from genotype data, and we then apply the same underlying logic to estimate within-family effects of genome-wide ancestry. We note that, without additional supporting evidence, a within-family causal effect on complex traits can be mediated by biological effects on a proximal trait and/or by social environments acting on distal traits (Supplementary Fig. 1). For example, in admixed populations where skin tone is associated with genome-wide ancestry, sibling differences in complex traits could be mediated by skin tone-associated discrimination (‘colourism’)^24,25^.

**Fig. 1:**
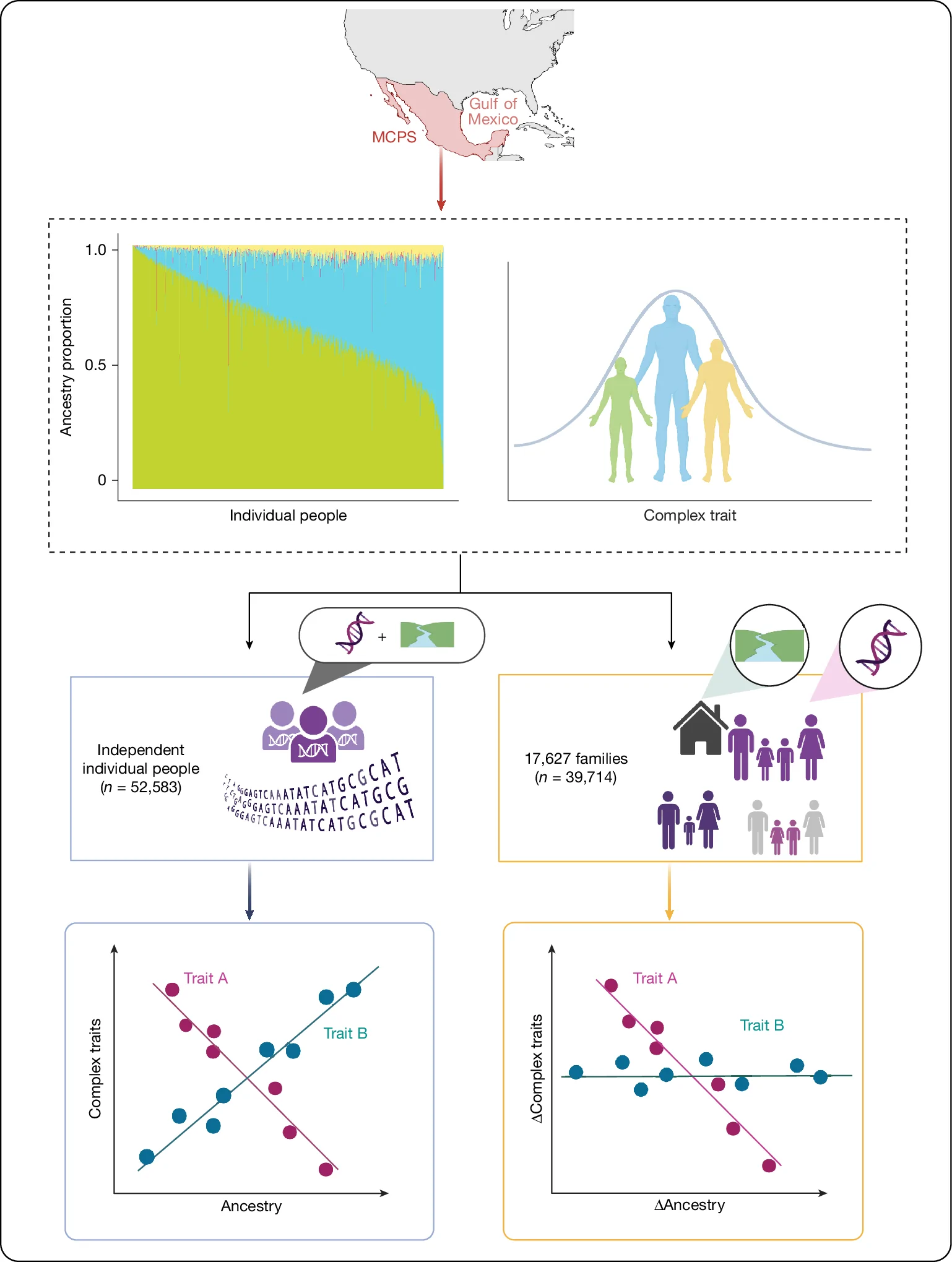
Graphical overview of the study. The aim of the study is to estimate within-family- and between-family-associated ancestry effects on complex traits using admixed samples from the MCPS. Phenotypic trait values and genome-wide ancestry proportions vary between people in the population and within families. Ancestry–trait associations were estimated in two subsets: a sample of *n* = 52,583 unrelated people and a sample of *n* = 39,714 people from 17,627 families. Comparing the two subsets allows the separation of within-family genetic effects, and environmental effects that are associated with ancestry, on traits. The same association in both sets is consistent with the association being driven by a within-family genetic effect (Trait A). Conversely, the absence of an association within families is consistent with an environmental explanation (Trait B). Figure created in BioRender; Wang, S. https://biorender.com/92284jd (2026).

With its large sample size, substantial genomic ancestry variation and more than 17,000 families, the Mexico City Prospective Study (MCPS)^3,4^ enables the quantification and dissection of associations between ancestry and complex traits. We use genome-wide genotyping data from related and unrelated people from the MCPS to estimate within-family effects of ancestry on 15 complex phenotypes, including anthropometric traits, biomarkers, T2D and educational attainment (EA). By studying a population with substantial inferred Indigenous American (IAM) ancestry, we aim to quantify and understand the between- and within-family effects of ancestry as an exposure on outcomes in Mexico City, which is relevant to population health in Mexico. An overview of the study design is provided in Fig. 1.

## Ancestry between and within families

Genome-wide proportions of inferred IAM, European (EUR), African (AFR) and East Asian (EAS) ancestries were derived for 140,829 people from the MCPS^4^ using participants from the 1000 Genomes Project (1KG) and the Human Genome Diversity Project (HGDP) as reference populations (Methods). We formed two subsets: a sample of 52,583 people who were unrelated up to the fourth degree and a sample of 39,714 participants from 17,627 first-degree families (full siblings and trios) (Fig. 1 and Extended Data Table 1; Methods).

On average, inferred genetic ancestry proportions were 67% (s.d. 18%), 29% (s.d. 16%), 4% (s.d. 3%) and less than 1% (s.d. 2%) of IAM, EUR, AFR and EAS ancestry, respectively (Supplementary Fig. 2 and Extended Data Table 2). As expected, inferred ancestry proportions were highly correlated with previously derived principal components (PCs) in the MCPS^4^ (Supplementary Fig. 3) as both PCs and admixture estimates are low-dimensional approximations of observed structure in genotype data^26^. The squared multiple correlation coefficient between ancestry proportion and PCs is up to 98% (Supplementary Table 1). From data on 2,847 families with both parents genotyped, we estimated the association between maternal and paternal genetic ancestry proportions and quantified the segregation variance within families. There is strong evidence for assortative mating on genetic ancestry, with correlations of 0.53 (95% confidence interval (CI), 0.50–0.55), 0.52 (95% CI, 0.49–0.55), 0.41 (95% CI, 0.38–0.44) and 0.02 (95% CI, −0.02–0.06) between estimated paternal and maternal ancestry proportions for IAM, EUR, AFR and EAS, respectively. Similarly, previously estimated PCs were correlated positively among parents (Supplementary Fig. 4). Consistent with the evidence of substantial assortative mating on genetic ancestry, the variance of ancestry proportions between families is much larger than the segregation variance. For IAM ancestry, the between-family s.d. is 0.167 whereas the within-family s.d. is only 0.020 (Extended Data Table 3). These results confirm that people in MCPS are highly admixed, that there is a positive correlation in estimated genome-wide ancestries between parents and that genome-wide ancestry proportions segregate within families.

## Genetic ancestries associate with traits

We quantified the association between genome-wide ancestry proportions and 15 complex traits in a sample of 52,583 unrelated people. For each trait, we regressed standardized trait values on genome-wide ancestry proportions and expressed estimated effect sizes (β, in s.d. units) relative to a 100% EUR baseline (Methods). Traits included height, weight, lipids and other biomarkers, T2D and EA (Extended Data Table 4; Methods). People in MCPS were recruited from two districts (Iztapalapa and Coyoacán) with different socio-economic profiles^4,27^. Supplementary Table 2 reports the study data separated by district.

Of the 45 trait–ancestry associations, 24 (13, 9 and 2 for IAM, AFR and EAS ancestry, respectively) were statistically significant at the study-wide significance *P* threshold of 5.89 × 10^−4^ (Methods), some with large effect sizes (Supplementary Table 3). IAM ancestry was associated significantly with height (β = −1.98, *P* < 2 × 10^−16^), body mass index (BMI, β = 0.27, *P* < 2 × 10^−16^), low-density lipoprotein (LDL) cholesterol (β = −0.69, *P* < 2 × 10^−16^), apolipoprotein B (ApoB, β = −0.43, *P* < 2 × 10^−16^), T2D (natural log odds ratio (lnOR) = 1.73 (95% CI, 1.54–1.92); *P* < 2 × 10^−16^) and EA (β = −2.03, *P* < 2 × 10^−16^). We also considered a stricter definition of controls for T2D and a finer-grained measure of EA (Methods). These sensitivity analyses showed a moderate increase in the effect of IAM on T2D risk and a small increase in the effect of IAM on EA (Supplementary Table 3). Genome-wide AFR ancestry proportions were also associated with several traits, such as glycated haemoglobin (HbA1c) (β = 0.92, *P* = 1.7 × 10^−8^), systolic blood pressure (SBP) (β = 0.91, *P* = 9.6 × 10^−9^) and EA (β = −3.24, *P* < 2×10^−16^). Relative to the EUR baseline, the IAM-associated effect sizes correspond to a reduction of approximately 13 cm in height and an increase in T2D prevalence of approximately 19 percentage points (Supplementary Table 3; Methods).

Taken together, these results show that genome-wide ancestry proportions are associated strongly with anthropometric traits, biomarkers, T2D and EA in MCPS, and that IAM ancestry is associated with increased T2D and its risk factors. However, these associations do not distinguish genetic effects from social and environmental factors correlated with inferred genetic ancestry. We therefore turned to the family data, where population-level associations can be partitioned into within-family ancestry effects and between-family ancestry associations (Supplementary Note 1), and applied an analytical approach based on Mendelian segregation.

## Ancestry associations within families

**Extended Data Fig. 1:**
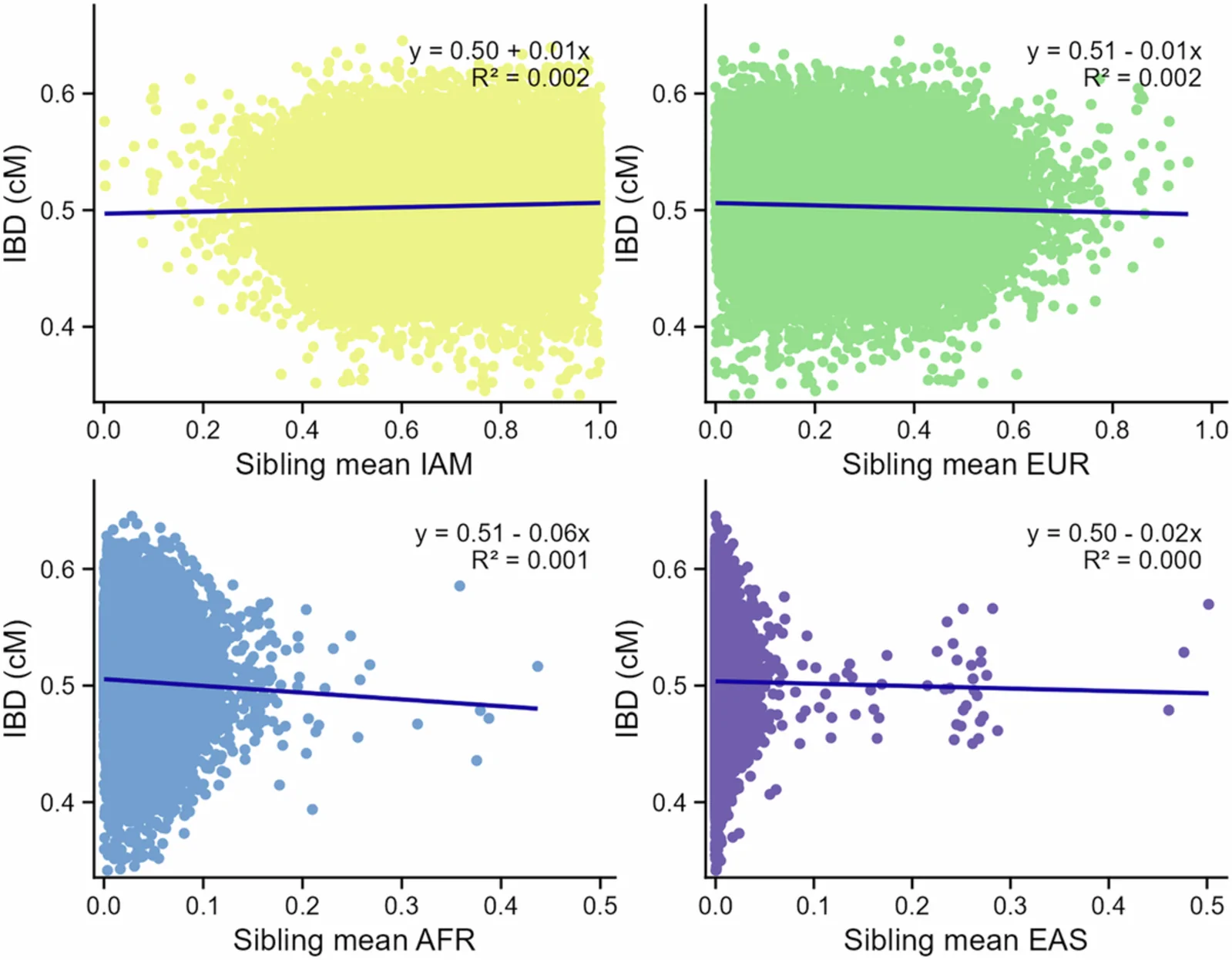
Inferred genome-wide Identity-By-Descent (IBD) proportions are uncorrelated with sibling mean ancestry proportions. Analyses were performed on n = 30,407 sibling pairs, using IBD estimated from snipar. Abbreviation: IBD = identical-by-descent, cM = centimorgan, IAM = Indigenous American, EUR = European, AFR = African, EAS = East Asian. Source data

We utilized 17,627 families from MCPS, comprising 30,407 sibling pairs, simultaneously estimating both between- and within-family effects of genetic ancestry, using linear mixed models to take account of the covariance structure of the data (Supplementary Note 1; Methods). Genome-wide identity-by-descent (IBD) proportions were estimated between all sibling pairs (Methods) and were uncorrelated with pair-wise estimated genetic ancestry (Extended Data Fig. 1). For families with no parental genotypes, we estimated the average parental ancestry proportions from the mean genetic ancestry of the genotyped offspring (Supplementary Fig. 5 and Supplementary Note 1).

**Fig. 2:**
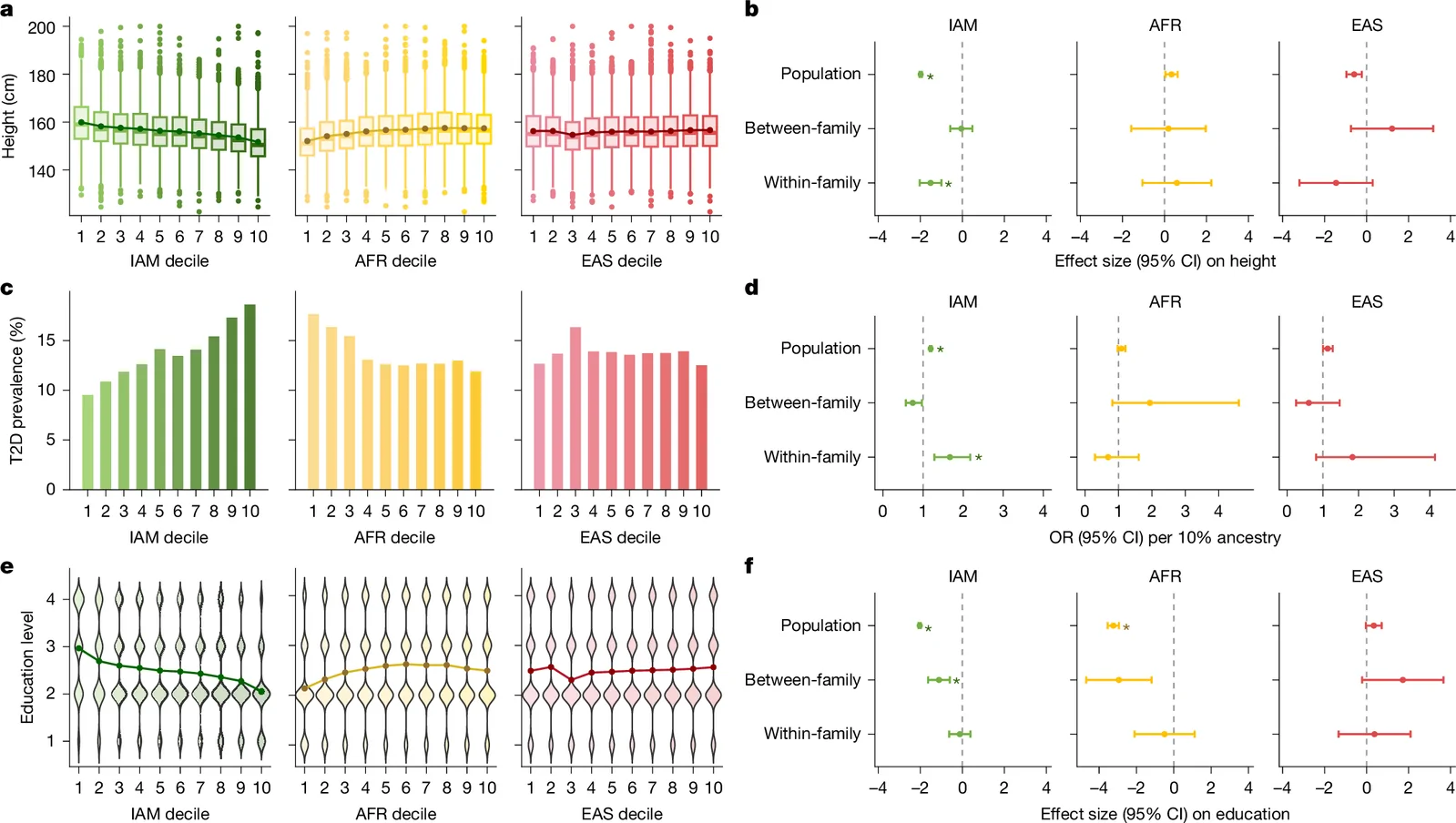
Between- and within-family effects of ancestry on height, T2D and EA. **a**, Height distribution across ancestry proportion deciles. Centre line, median; box, interquartile range; whiskers, 1.5× interquartile range; points beyond whiskers, outliers; dots, mean height for each decile (*n* = 52,064). **b**, Estimated ancestry effects on height. Points, effect size estimates. **c**, T2D prevalence by ancestry deciles in the population (*n* = 46,992). **d**, Effect size in odds ratio (OR) per 10% ancestry on T2D. **e**, EA level distribution across ancestry deciles in the population (4, university or high school; 3, middle school; 2, elementary school; 1, illiterate or literate). **f**, Estimated ancestry effect on EA level. Points, effect size estimates. In **b**, **d** and **f**, error bars represent 95% CIs. Population estimates were obtained using linear (**b**, **f**) or logistic (**d**) regression, whereas between- and within-family estimates were obtained using linear mixed models (**b**, **f**) or a generalized linear mixed model with a single random effect of family fitted for T2D (**d**; Methods). Two-sided *P *values are provided in Supplementary Tables 3 and 4. Asterisks, study-wide significant associations after adjustment for multiple testing (*P < 5.89 × 10^−4^). For each trait and each ancestry, population estimates are derived from unrelated people (*n* = 52,064 for height, *n* = 46,992 for T2D, *n* = 52,558 for EA). Between- and within-family effect sizes were estimated from family data (height, *n* = 39,298 people from 17,614 families; T2D, *n* = 35,385 people from 17,301 families; education level, *n* = 39,698 people from 17,627 families). Note that the mean ancestry proportions in the deciles vary across ancestries (**a**, **c**, **e**). For IAM, AFR and EAS, the mean ancestry proportions in the first and tenth deciles are (0.32, 0.95), (0.01, 0.11) and (10^−5^, 0.03), respectively. Source data

**Fig. 3:**
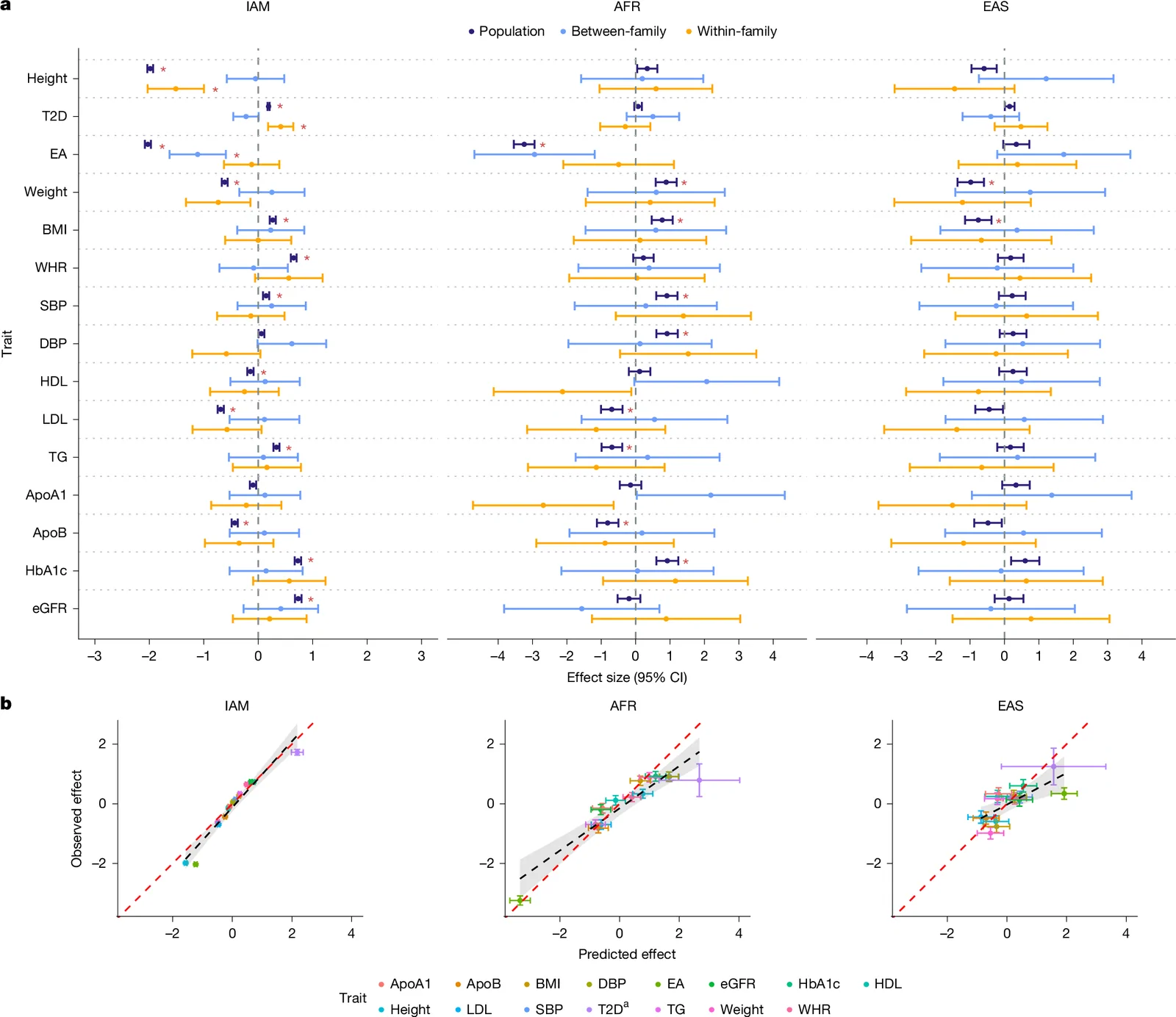
Between- and within-family effects of ancestry on all 15 traits. **a**, Effect of different ancestries on all study traits. Red asterisk, study-wide significance (*P < 5.89 × 10^−4^; Methods). Points, effect size estimates; error bars, 95% CIs. All population-level effect sizes shown were estimated from a linear model, and between- and within-family effect sizes were estimated by a mixed linear model, using the observed 0–1 scale for T2D. All *P *values are two-sided. Exact *P *values are provided in Supplementary Tables 3 and 4. **b**, Comparison between the ancestry effect sizes at the population level from the sample of unrelated individuals (*y* axis, ‘observed’) and their estimates predicted (Methods) from the analysis of the family data (*x* axis, ‘predicted’). Points, β estimates; horizontal and vertical error bars, ±1 s.e. Black dashed line, fitted linear regression line; grey shaded area, 95% CI. Red dashed line, reference line *y* = *x*. ^a^For T2D, effect sizes are in lnOR units, with the effect sizes from the family data estimated from a generalized linear mixed model with a single random effect of family (Methods). For each trait and each ancestry, population estimates are given from a sample of unrelated people, with sample sizes ranging from 40,744 to 52,558 (Extended Data Table 4). Between- and within-family estimates are from family data, with sample sizes ranging from *n* = 30,500 (16,198 families) to *n* = 39,698 (17,627 families) (Extended Data Table 4). ApoA1, apolipoprotein A1; DBP, diastolic blood pressure; eGFR, estimated glomerular filtration rate; HDL, high-density lipoprotein cholesterol; TG, total triglycerides; WHR, waist-to-hip ratio. Source data

**Extended Data Fig. 2:**
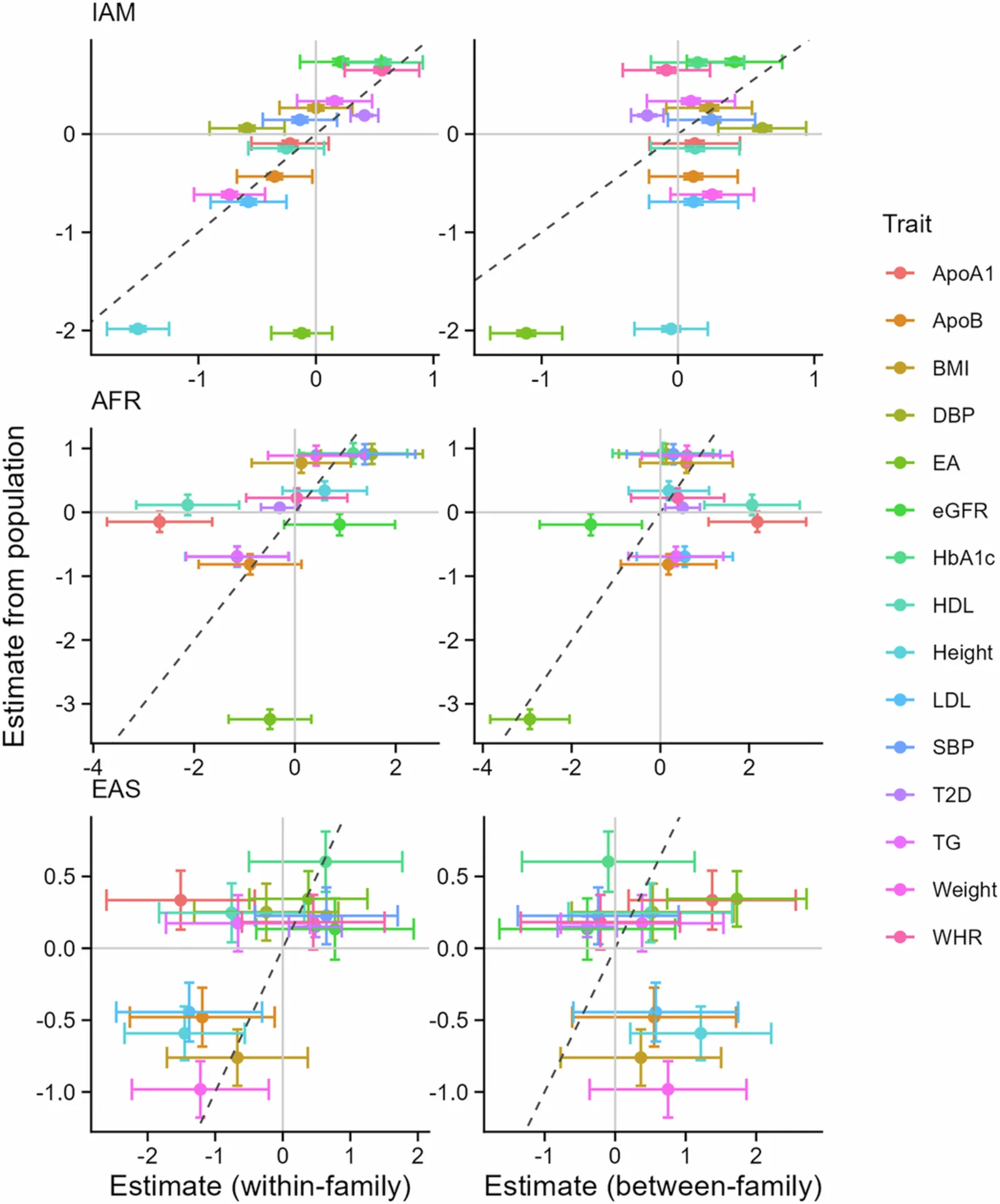
Comparison of ancestry proportion effects on all study traits from population and family data. All population-level effect sizes shown in the figure were estimated from a linear model, and between- and within-family effect sizes were estimated by a mixed linear model, using the observed 0-1 scale for T2D. Population-level estimates were obtained from a sample of unrelated individuals, with sample sizes ranging from 40,744 to 52,558 (Extended Data Table 4). Between- and within-family estimates were obtained from family data, with sample sizes ranging from 30,500 individuals (16,198 families) to 39,698 individuals (17,627 families), depending on the phenotype (Extended Data Table 4). Horizontal error bars represent ±1 standard error of the within-family estimates (left) and between-family estimates (right), whereas vertical error bars indicate ±1 standard error of the population-level estimates. Abbreviation: IAM = Indigenous American ancestry; AFR = African; EAS = East Asian; ApoA1 = apolipoprotein A1; ApoB = apolipoprotein B; BMI = body mass index; DBP = diastolic blood pressure; EA = educational attainment; eGFR = estimated glomerular filtration rate; HbA1c = glycated haemoglobin; HDL = high-density lipoprotein; LDL = low-density lipoprotein; SBP = systolic blood pressure; T2D = type 2 diabetes; TG = total triglycerides; WHR = waist-to-hip ratio. Source data

For the 90 trait–ancestry combinations, we found three significant associations within families (Supplementary Table 4). There was a within-family ancestry effect of IAM ancestry on height −1.51 s.d. (95% CI, −2.03 to −1.00; *P* = 1 × 10^−8^), whereas the between-family ancestry effect was not statistically significant (Fig. 2), consistent with the population-level associations being driven by genetic effects, which are accounted for by the within-family effect. Similarly, we found a strong within-family ancestry effect of IAM on T2D risk (lnOR = 5.13, 95% CI 2.48–7.78; *P* = 1.51 × 10^−4^). In contrast, the estimated within-family ancestry effect for EA was close to zero and not statistically significant, despite a large and significant (β = −1.11, *P* = 2.51 × 10^−5^) between-family association (Fig. 2). The associations of ancestries with all study traits are shown in Fig. 3a and Extended Data Fig. 2.

**Extended Data Fig. 3:**
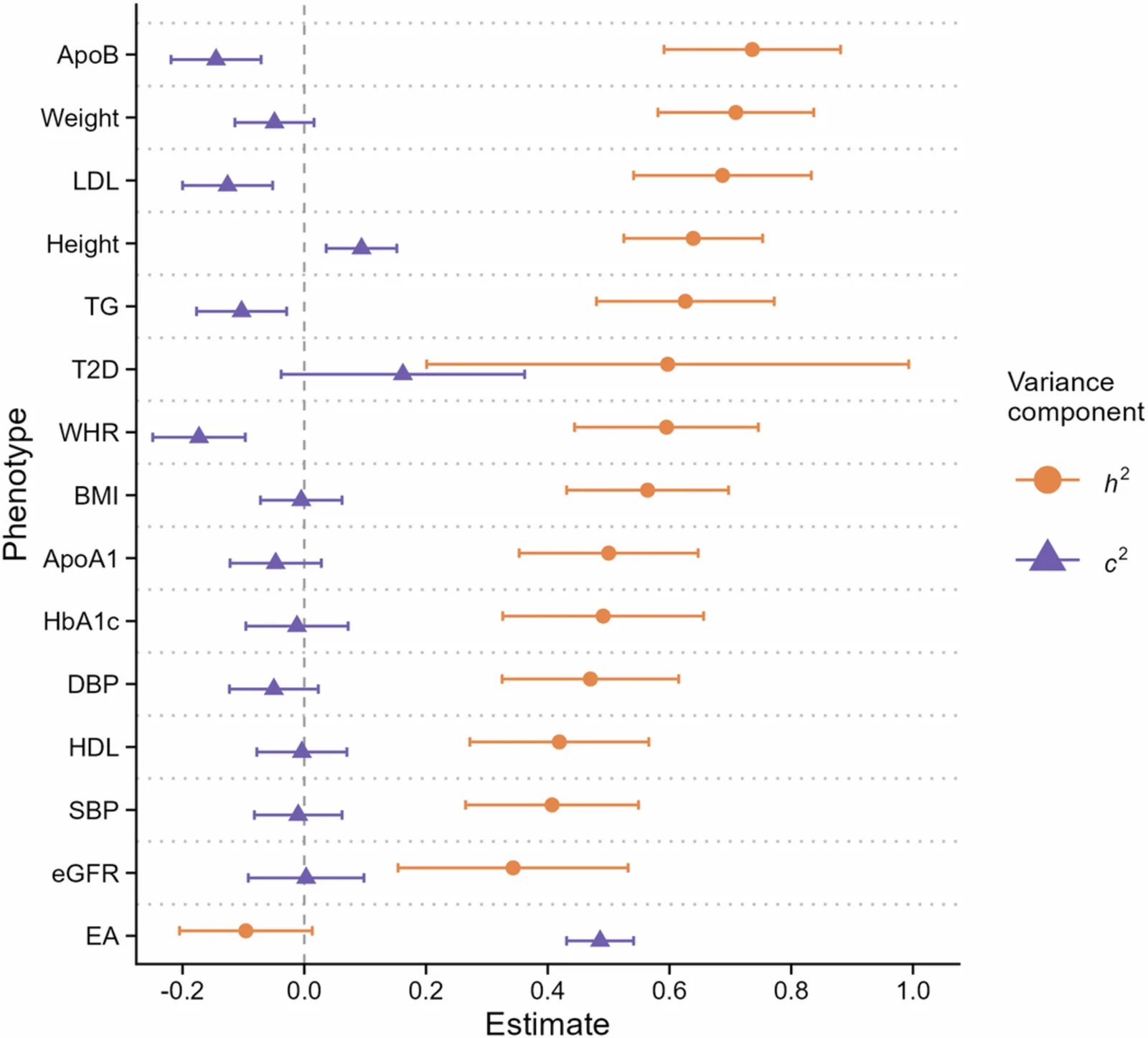
Variance components estimation. Forest plot showing estimates of the proportion of phenotypic variance explained by IBD sharing (*h*^2^) and other effects (*c*^2^). Error bars represent standard errors. Results are presented for T2D on the scale of liability. Variance components were estimated from family data, with sample sizes ranging from 30,500 individuals (16,198 families) to 39,698 individuals (17,627 families) across phenotypes (Extended Data Table 4). Abbreviation: ApoB = apolipoprotein B; LDL = low-density lipoprotein; TG = total triglycerides; T2D = type 2 diabetes; WHR = waist-to-hip ratio; BMI = body mass index; ApoA1 = apolipoprotein A1; HbA1c = glycated haemoglobin; DBP = diastolic blood pressure; HDL = high-density lipoprotein; SBP = systolic blood pressure; eGFR = estimated glomerular filtration rate; EA = educational attainment. Source data

As a by-product of the family analyses, we obtain estimates of within-ancestry genetic segregation variance and estimates of variance due to effects that are common to siblings but not explained by IBD sharing^28,29^. These residual sharing effects could be caused by common environmental factors or assortative mating^29–31^. Estimates of heritability and the proportion of variance due to residual sharing effects are shown in Extended Data Fig. 3 and Supplementary Table 5. Although the s.e. values are large, estimates of heritability are substantial and much larger than estimates of residual shared variance for nearly all traits. An exception is EA, where the data are consistent with all covariance among siblings being due to residual shared factors. The finer-grained measure of EA gave similar results (Supplementary Table 5). Given the large s.e. of the estimate, the apparent lack of genetic variation could be due to sampling error. It could also be obscured by the effect of assortative mating.

The estimates obtained from the family data predict the expected effect size from a population sample without family data (Supplementary Note 1). Figure 3b shows strong concordance across all traits when comparing the effect sizes from the independent population and family samples.

In summary, among the 15 complex traits studied with the family data, we report evidence of a within-family ancestry effect on height and risk of T2D and a between-family effect on EA.

## Trait alleles associate with ancestry

The within-family effect of ancestry on height and T2D is consistent with a substantial genetic contribution but uninformative with respect to genetic architecture. To investigate whether the estimated within-family effects could be due partly to ancestry differences in the frequency of trait-associated variants, we estimated a count of the number of trait-increasing alleles (cTIA) for each person in the population sample from multi-ancestry genome-wide association analysis study (GWAS) for height^32^ and T2D^33^ and correlated it with genome-wide ancestry proportions (Methods). For height, we found a large negative partial correlation between genome-wide IAM ancestry and cTIA (*r* = −0.46, *P* < 1 × 10^−300^). Significant but small partial correlations were observed for AFR (*r* = −0.10, *P* = 1.17 × 10^−123^) and EAS (*r* = −0.06, *P* = 7.27 × 10^−40^). For T2D risk, there was a positive partial correlation between IAM ancestry and cTIA (*r* = 0.35, *P* < 1 × 10^−300^) and positive but smaller correlations for AFR (*r* = 0.12, *P* = 2.58 × 10^−176^) and EAS (*r* = 0.05, *P* = 9.61 × 10^−35^). A population specific cTIA from variants is unlikely to be an unbiased reflection of the true mean genetic value of that population as lead single-nucleotide polymorphisms (SNPs) from GWAS are not necessarily causal variants and linkage disequilibrium (LD) patterns vary by population. Conditional on being associated with a trait, coding variants are more likely to be causal^34–36^. We therefore considered a cTIA of missense and loss-of-function variants, identifying 314 for height and 36 for T2D (Methods). Using the coding variant cTIA attenuated the association with genetic ancestry for height and gave inconclusive effects for T2D (Supplementary Table 6), probably owing to a combination of a small number of variants and rare variant ascertainment bias, as most of the 36 missense variants have low minor allele frequency in EUR (Supplementary Table 7), where most of the GWAS discovery data were ascertained. The observed partial correlations between cTIA and ancestry proportions remained statistically significant for the full set of associated variants when compared with random permutations of the label of the trait-increasing allele at each associated locus (Methods), but not when considering the smaller set of coding variants (Supplementary Fig. 6).

If the cTIA captures a proportion of the within-family ancestry effect we estimated in the family data, then adjusting for it should attenuate their estimated effect sizes. We therefore estimated the effects of ancestry and cTIA simultaneously in the family analysis and observed a significant attenuation (Supplementary Table 8). The IAM within-family effect on height changed from −1.51 s.d. to −0.43 (*P* > 0.05) and the IAM effect (lnOR) on T2D from 5.13 to 3.37 (*P* = 0.01). A smaller effect of IAM on height but no change for T2D was observed when the missense cTIA was included (Supplementary Table 8). To test for robustness of the within-family results, we performed sensitivity analyses in which we fitted cTIA values for traits that did not correspond to the outcome of interest (for example, fitting the cTIA for T2D in the analysis of height). We also quantified the within-family results after including EA as a proxy for socio-economic indicators. These analyses did not result in any meaningful change of the within-family estimates (Supplementary Table 8).

These findings show that the within-family ancestry effects for height and risk of T2D are consistent with differences in the number of trait-increasing alleles between ancestries involving variants that are segregating across populations.

## Selection on height and T2D loci

**Fig. 4:**
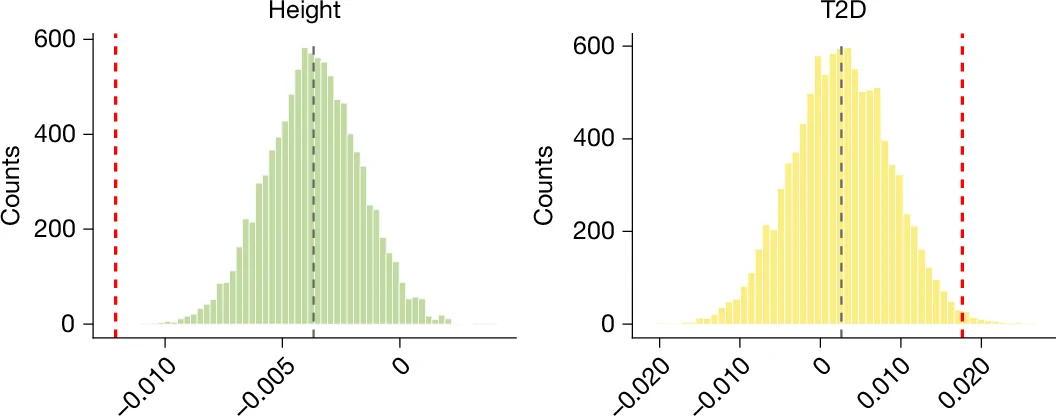
Mean difference in the frequency of trait-increasing alleles between IAM and EUR populations for height and T2D. Histogram, null distribution of mean fTIA differences from matched control sets; grey dashed line, mean value; red dashed line, mean value of the fTIA difference for the trait-associated SNPs. Control SNPs were matched on frequency and LD scores of the genome-wide significant loci and repeatedly sampled from the GWAS summary statistics (Methods). Source data

We investigated whether the genetic differences between IAM and EUR ancestries for height and T2D could have been driven by natural selection, using estimated allele frequencies from samples that were approximately 100% IAM or 100% EUR ancestry. We obtained IAM allele frequencies by selecting 1,012 people from MCPS whose estimated IAM ancestry in the genome was larger than 99.5% and validated those against frequencies from IAM in the HGDP (Supplementary Fig. 7; Methods). We then estimated the mean fixation index (*F*_ST_) at the trait-associated loci between IAM and EUR ancestry populations for height and T2D and compared the estimates with control SNPs (Methods). Mean *F*_ST_ of associated loci was significantly higher than that of matched control SNPs for height (0.120 versus 0.116, *P* = 2.16 × 10^−4^), but not for T2D (0.110 versus 0.109, *P* = 0.83) (Supplementary Fig. 8). We subsequently calculated the IAM–EUR difference in frequency of trait-increasing alleles at loci that were associated statistically with height and T2D in the GWAS discovery sets^32,33^ and compared those with frequency differences of trait-increasing alleles at control SNPs in the GWAS that were matched on frequency of trait-increasing alleles (fTIA) and LD score (Methods). The top 20 loci with the largest difference in fTIA between IAM and EUR for height and T2D are shown in Supplementary Table 9. The largest difference in fTIA is −0.87 for height (rs174570) and 0.75 for T2D (rs12625671). Figure 4 shows that, for both traits, there is a systematic and significant difference in trait-increasing allele frequency, in the direction consistent with IAM having a lower load of height-increasing alleles (*P* = 2.16 × 10^−5^) and a higher load of T2D-increasing alleles (*P* = 1.48 × 10^−2^). Our test for selection is conservative because our control SNPs can also be associated with the trait even when not in LD with genome-wide significant loci, in particular for height where a randomly chosen SNP in the discovery data is genome-wide significant^32,37^. This is a likely explanation of why the mean frequency difference of the null distribution is not zero and in the direction of the genetic mean difference (Fig. 4). As an additional control, we performed the same analyses on two traits, SBP and BMI, with well-powered GWAS summary statistics^38,39^ for which there is no evidence of a genetic difference between IAM and EUR ancestries. The results show no systematic difference in fTIA for these traits (Supplementary Fig. 9).

If genetic differences are due partially to selection, then loci that explained more trait variation when selection occurred should be more differentiated in frequency. We therefore correlated the difference in trait-increasing allele frequencies with their predicted amount of variance explained in EUR (Methods), but did not detect a statistically significant association (Supplementary Fig. 10).

The results from these analyses imply that the genetic difference between IAM and EUR ancestries in polygenic load for height is driven partially by natural selection acting upon height-associated variants, whereas the evidence for selection on T2D-associated variants is inconclusive.

## Discussion

We exploited the random segregation of parental chromosomes during meiosis to estimate within-family effects of genetic ancestry differences on complex traits in an admixed sample from Mexico City. In principle this design can be applied whenever there is phenotypic and genome-wide genetic data from admixed families, enabling the separation of within-family ancestry effects from population-level correlations between genetic ancestry and phenotype owing to other factors. By conducting a large family analysis in an admixed population, we have provided evidence that between-ancestry differences for complex traits can be genetic and explained by genome-wide differences in the frequency of trait-increasing alleles. Specifically, we found strong evidence for an IAM–EUR genetic difference in height and T2D risk in the MCPS, consistent with the direction of phenotypic differences and polygenic load between these ancestries. Our study provides both a template on how to perform robust ancestry associations on complex traits (including disease) with family data while modelling the covariance between siblings, and provides calculations that identify the sources of information that determine statistical power and quantifies future sample sizes needed to perform these analyses in different ancestries.

Although our within-family ancestry effect estimates can be given a causal interpretation, they do not imply a specific mechanism. It is possible that ancestry differences cause phenotype differences through social mechanisms such as ancestry-related appearance-based discrimination. Previous studies have reported between-sibling differences in skin tone that are associated with health and other outcomes^24,25^. Because these studies are based upon full siblings and without genome-wide genetic data, they cannot distinguish between a model in which genome-wide ancestry causes differences in both skin tone and disease susceptibility and a model in which genome-wide ancestry differences cause skin tone differences, which cause differences in social environments that predispose to disease (Supplementary Fig. 1). Our estimates are consistent with ancestry associated phenotype differences in height and T2D risk being explained entirely by within-family ancestry effects, but the absence of a between-family effect does not imply or prove a mechanism by itself. When we fitted EA in the model as a proxy for social environment, there was no change in the estimates of the within-family effects for height and T2D (Supplementary Table 8), implying that any hypothetical social mechanism is not correlated with EA.

At the population level, genetic ancestry effects were large and statistically significant for most traits, yet most of these estimates did not carry over when estimating their effects within families. The difference was particularly striking for EA, for which extremely large associations with ancestry (of 2 s.d. or more) were estimated at the population level. However, our family-based analysis suggests that environmental factors correlated with ancestry rather than causal effects of alleles that differ in frequency between IAM and EUR ancestries explain the association between genetic ancestry and EA in the MCPS. This shows that associations between genome-wide ancestry and phenotypes—especially socio-economic phenotypes—in the absence of family data could lead to misleading inference^40^. In addition to no evidence for a within-family genetic ancestry effect on EA, we found no evidence for any within-family genetic segregation variance in the MCPS (Extended Data Fig. 3). However, the s.e. of the estimate of heritability is large (0.11), the residual sharing effects may contain effects due to assortative mating and there is evidence for genetic variation associated with EA in populations of EUR ancestry^41,42^. Given the s.e. of the within-family estimate, we had sufficient statistical power to detect effect sizes of 1 s.d. unit or more (Supplementary Notes 1 and 2).

It is not known whether our findings translate to phenotypic differences between non-admixed IAM and EUR populations. Our inference is strictly on MCPS participants from Mexico City, and our estimates are weighted towards offspring of parents with more ancestry differences between their paternal and maternal genomes, analogous to how family-based genetic association estimates are weighted towards families whose parents have greater levels of heterozygosity^43^. This implies that within-family ancestry effect estimates from admixed families may differ from the genetic contribution to phenotype differences between more homogeneous samples. Systematic differences could be induced by assortative mating with respect to both phenotype and ancestry and whether effects of alleles depend on genetic and environmental background (gene–environment and gene–gene interactions). Furthermore, environmental effects may differ in un-admixed populations compared with the admixed samples used here.

Lack of concordance of estimated effect sizes within families compared with those estimated in the population sample can also be due to reduced statistical power. At the population level, the precision of estimated effect sizes is proportional to the product of sample size and the between-family variance in ancestry proportion, whereas it is approximately proportional to the product of the number of sibling pairs and the within-family variance for the estimated effect size within families (Supplementary Notes 1 and 2). For IAM ancestry, the ratios of sample sizes and ancestry proportion variances are 0.58 and 0.014, respectively (Extended Data Tables 2 and 3), implying a difference in precision of more than 100-fold. Precision is also lower for binary traits compared with quantitative traits, as shown in our results for T2D. Although the estimate of the within-family effect of IAM ancestry is statistically significant, the estimate is extremely large and imprecise (the 95% CI of the lnOR is 2.48–7.78) and its sampling correlation with the between-family IAM effect is −0.99 (Supplementary Table 10). The estimated effect sizes of the statistically significant effects are also probably over-estimated owing to the winner’s curse (selection bias). Therefore, even larger sample sizes are needed to separate within-family and between-family ancestry effects in admixed populations for common diseases. In Supplementary Note 2 we provide power calculations to detect significant within-family effects in admixed populations.

In the MCPS we found strong evidence that IAM ancestry has a within-family effect on the risk of T2D and is enriched for common T2D risk alleles. This provides evidence of a genetic difference in susceptibility to a common disease between ancestries that is associated with differences in disease prevalence. There is a high prevalence of T2D in Mexico^44,45^, and diabetes is a leading cause of premature death in Mexico^46^. Our results are strongly supportive of a within-family, ancestry-related genetic contribution to T2D burden in this population, beyond environmental influences such as a diabetogenic diet and limited healthcare access. Notably, the association between T2D burden and IAM ancestry does not seem to be explained by environmental factors shared within families.

We provided evidence that natural selection has acted upon height- and T2D-associated loci, in a direction consistent with our within-family estimates. These analyses are not informative about where and when selection took place or its mechanism. Evidence of selection for reduced height has been reported in people of Peruvian ancestry^47^, from the observation that a trait-decreasing *FBN1* missense variant (rs200342067) was at much higher frequency (0.047) in Peru than in Europe (<0.001). The frequency of that variant is 2.27% in the MCPS sample of 1,012 people with >99.5% inferred IAM ancestry and the estimated effect size is also large (−0.29 s.d., or approximately −2 cm, *P* < 2 × 10^−16^ among the set of unrelated people). Our results are consistent with selection for reduced height being widespread in Central and South America. The study in people from Peru also estimated the association between ancestry proportions and height at the population level and reported an IAM ancestry effect of −14.75 cm relative to a European ancestry baseline, remarkably close to the estimate in the MCPS of −2 s.d., which is approximately −13 cm. Of note, rs174570 has the greatest fTIA difference for height between IAM and EUR in the present study (Supplementary Table 9). It lies within the *FADS2* gene that encodes an enzyme involved in the production of long-chain polyunsaturated fatty acids^48^. Selection at the *FADS2* region showed opposite patterns in Greenlandic Inuit^49^ and Europeans, reflecting adaptation to marine versus plant-based diets, respectively^50^.

Our approach is similar to that of Lin and colleagues^5^, who estimated ancestry effects between and within families on 26 traits in 697 families with Inuit–European admixture in Greenland. The authors reported significant within-family ancestry differences for weight-associated phenotypes and, consistent with a partitioning analysis (Supplementary Note 1), concordance in effect sizes estimated at the population level and within families. Their estimate for height was −14 cm for Inuit ancestry at the population level and −3.5 cm (s.e. 6.8 cm, statistically not significantly different from zero) estimated within families. Statistical significance for each trait was determined from a false discovery rate approach for each parameter tested, which is less stringent than our approach.

Recently, a within-family analysis of nine complex traits in White British participants from the UK Biobank compared the effects of genetic PCs estimated within sibships and in the population^51^. Similar effect sizes were observed in both analyses, suggesting that genetic PCs capture inherited genetic differences in addition to environmental confounding. Together with our findings, these results indicate that genome-wide summaries of population structure, such as genetic PCs and ancestry proportions, can capture trait-specific effects arising from inherited genetic differences.

Our study has a few limitations. We focus on a select number of traits from a single admixed population where most admixture is between ancestral Mesoamerican and European populations, sampled from a single location (Mexico City). Additional data from IAM–EUR admixed populations sampled in different locations would show how general our conclusions are regarding the differing effects of IAM and EUR ancestries. Additional data on more traits and diseases in other admixed populations would allow a fuller quantification of between-population mean genetic differences for complex traits.

In summary, our study shows that within-family analyses of admixed families can be used to estimate the effects of genetic ancestry on complex traits and diseases. Using data from the MCPS, we found evidence for a within-family effect of IAM ancestry on height and risk of T2D relative to European ancestry, and that these ancestry differences were shaped, at least in part, by natural selection.

## Methods

### Study population and ethics declaration

The MCPS is a prospective cohort of over 150,000 adult participants from Mexico City^3,4^. The baseline survey took place between 14 April 1998 and 28 September 2004 and focused on households in two urban districts, Coyoacán and Iztapalapa. Residents aged 35 years or older were invited to participate in the study. Of the 112,333 households with eligible residents, at least one person from 106,059 households participated^3,4^.

### Admixture analysis and estimation of genome-wide ancestry proportions

Whole-genome sequence data from the 1KG and the HGDP were downloaded and filtered to the set of autosomal variants present on the Illumina Global Screening Array v.2 (GSAv.2). These datasets were then merged with the MCPS GSAv.2 array dataset with 140,829 participants (previously quality controlled as described in ref. ^4^ with the adjustment of filtering for genotype missingness before filtering for individual missingness, allowing us to retain 2,318 more participants than the 138,511 people evaluated in that study). This resulted in a merged dataset of 485,043 unambiguous bi-allelic SNPs. 1KG and HGDP participants representing four global ‘superpopulations’ were designated as reference samples and included 765, 727, 658 and 408 people of AFR, EAS, EUR and IAM ancestry, respectively. This reference set was then supplemented with 1,000 randomly selected MCPS participants unrelated to the fourth degree, resulting in a total of 1,408 IAM and MCPS samples and 3,558 reference samples overall.

Ancestry-specific allele frequencies and per-person ancestry proportions were estimated with ADMIXTURE^13^ v.1.3.0. An admixture model was fit for *K* = 4 ancestral populations inferred among the set of reference participants using an unsupervised procedure. The choice of setting *K* = 4 was guided by previous work that showed that the continental-level ‘superpopulations’ most represented by genetic similarity in the MCPS cohort were IAM, EUR and, to a lesser extent, AFR and EAS^4^. Ancestry proportions for the remaining set of 139,829 MCPS participants were then estimated by projection with the -P option. Each of the estimated *K* ancestries was assigned to a global ‘superpopulation’ based on averages within the reference samples (for example, the ancestry with the highest average proportion among EUR reference samples was assigned to an inferred EUR ancestral population). Ancestry proportions from each of the four inferred ancestral populations were used in subsequent analyses.

### Selection of population and family samples

From the 140,829 participants we selected two non-overlapping subsets, a set of families (consisting of two or more genotyped siblings and zero, one or two genotyped parents; Extended Data Table 1) and a separate set of people who were not related up to the fourth degree of relatedness. Pair-wise relatedness for all samples was inferred with KING^52^, using the option --ibdseg, as described previously^4^. From the 30,450 sibling pairs identified by KING, those identified as full siblings were grouped into family units. To ensure that all pairs within a family were full siblings according to KING’s estimation, 25 people were removed. Furthermore, four people were excluded to ensure that each family had no more than two putative parents. The filtration resulted in a family dataset comprising 30,407 full-sibling pairs within 16,187 families (*n* = 38,274). To refine the family structure further, we incorporated parent–offspring relationships, identifying people with genotype data for both parents, resulting in 1,440 complete parent–offspring trios. Consequently, the family-based dataset comprised 39,714 people with either full siblings or two genotyped parents. The ‘population sample’ was selected from the 63,130 people who were unrelated up to the fourth degree of relatedness, excluding family set people and their parents, leading to a sample set of 52,583. Restriction to this unrelated set was done to minimize confounding due to pedigree relatedness in estimating population-level effects.

### Estimation of IBD sharing among siblings

For each identified family, genome-wide IBD was estimated with snipar (single-nucleotide imputation of parents)^53^ based on genotype array data. snipar employs a hidden Markov model to infer IBD segments shared between siblings, achieving near-theoretical accuracy and reducing IBD errors compared to KING^53^. When running snipar, genotyping array variants were filtered based on the following criteria: minor allele frequency less than 5%, significant deviation from Hardy–Weinberg equilibrium (*P* < 1 × 10^−4^), or missingness greater than 1%. Furthermore, a genotyping error probability threshold of 4.5 × 10^−4^ was applied.

A comparison of estimated genome-wide IBD proportions among sibling pairs using either genetic or physical genome length is shown in Supplementary Fig. 11. For subsequent analyses, we used the realized relationships among sibling pairs estimated from snipar, with IBD defined as the genome-wide proportion of genetic map length (in centimorgans) shared.

### Phenotype selection

Fifteen complex traits data at baseline were explored in the analysis, including height, weight, BMI, waist-to-hip ratio (WHR), SBP, diastolic blood pressure (DBP), high-density lipoprotein cholesterol (HDL), LDL, total triglycerides, apolipoprotein A1 (ApoA1), ApoB, HbA1c, estimated glomerular filtration rate (eGFR), an ordinal categorical variable, EA, and one disease, T2D. eGFR was calculated using the formula of the Chronic Kidney Disease Epidemiology Collaboration 2021, which considers age, sex, and creatinine plasma concentration^54^. SBP and DBP were adjusted by adding 15 or 10, respectively, separately if the participants took antihypertension medication^55^. EA was represented by a category variable, education (4, university or high school; 3, middle school; 2, elementary; 1, illiterate or literate), which is treated as a continuous trait in the analysis. As a sensitivity analysis we also considered a finer-grained measure of education using 14 categories (Supplementary Table 11). The inverse normal distribution function was used to derive category thresholds for the 14 categories from their cumulative probabilities, and the mean *z*-score for each category was calculated assuming an underlying normal distribution with several thresholds. This transformation placed the 14 ordinal EA categories on an underlying normal scale. The phenotypic correlation between the 1–4 scale and the 14-category *z*-score scale in the entire MCPS cohort was 0.94.

People were considered to have T2D if they reported a previous diagnosis of diabetes (diagnosed age at least 35 years) or were taking anti-diabetic medication at baseline. People who reported a diagnosis before age 35 years and were on insulin were considered likely to have type 1 diabetes and were excluded from the T2D case set. Participants without a previous diabetes diagnosis or on anti-diabetic medication and with HbA1c < 6.0% were classified as normoglycaemic controls. The American Diabetes Association uses an HbA1c threshold <5.7%^56^, and therefore we used this threshold to select controls in a sensitivity analysis. Phenotype outliers were excluded if height was less than 120 cm or greater than 200 cm, weight less than 35 kg or greater than 250 kg, BMI (kg m^−2^) less than 15 or more than 60, and WHR less than 0.5 or more than 1.5. To make the effect scale comparable across phenotypes, continuous traits were adjusted for age and age^2^ and standardized (mean 0, s.d. 1) within each sex, and outliers were further excluded according to mean ± 5 s.d. Further adjustments were applied for specific traits: BMI was residualized in the standardization for WHR, and fasting duration was residualized for HDL, LDL, total triglycerides, ApoA1 and ApoB. Stata (v.18.5) was used for phenotype data processing.

### Multiple testing adjustment

To determine the statistical significance of estimated ancestry effects, we calculated the total number of independent tests and adjusted the *P* threshold accordingly. Each trait had three estimates (one from the population sample and two from the family sample) for each of the three ancestries, resulting in a total of nine comparisons per trait. Given that the 15 traits were correlated (Supplementary Table 12), we estimated the effective number of independent traits using the eigenvalues of the phenotypic correlation matrix^57^. The estimated number of independent traits was 9.43, leading to a study-wide significance threshold of 5.89 × 10^−4^ (0.05/(9 × 9.43)).

### Estimation of ancestry effects

For the sample of unrelated people, linear regression analyses were conducted using R (v.4.3.2) to estimate the association between ancestries and complex traits. District (Iztapalapa or Coyoacán) was included as a covariate for all traits. Sex, age and age^2^ were included as covariates for T2D analyses. HbA1c and eGFR were analysed exclusively in the subset of non-diabetic people, defined as those without a T2D diagnosis, not taking anti-diabetic medication and with HbA1c under 6.5%^58^. Furthermore, sensitivity analyses were performed, adjusting for the first seven genetic PCs or without fitting district as covariates (Supplementary Table 13). Note that only the first seven PCs were used as these PCs had normally distributed SNP loadings across the genome—a signature of population structure—as opposed to non-normally distributed loadings indicative of long-range LD^4,59^.

Quantitative traits in the family data were analysed using linear mixed models, fitting individual genetic effects and shared environmental effects as random effects and between- and within-family ancestry effects (IAM, AFR and EAS) as fixed effects (Supplementary Note 1). These analyses were performed in GCTA^60^ (v.1.94.3). The covariance structure between the phenotypes of siblings was modelled by fitting the estimated IBD relationship matrix and a matrix for shared environmental effects, as done previously^28,29^. These models simultaneously estimate between- and within-family ancestry effects and variance components for genetic and shared environmental effects.

Ancestry association analyses were conducted with EUR ancestry specified as the reference. IAM and EUR ancestries together account for most (greater than 90% on average) genome-wide inferred ancestry in this population, and model identifiability constraints permit the inclusion of only three of the four ancestry components. Retaining IAM in the model therefore enabled a more interpretable result in this study population where genetically inferred IAM ancestry comprises the largest proportion.

### Analysis of T2D

T2D was the only binary (0–1) trait and was analysed using both linear (mixed) models and generalized linear (mixed) models. The statistical software packages we used did not have an option for a generalized linear mixed model with multiple random effect and user-specified covariance structures. We did have that option for linear mixed models and used that for T2D so that on the 0–1 scale it is the same model as for the quantitative traits. For the population set, we first fitted a simple linear model using lm in R (v.4.3.2) and subsequently fitted a generalized linear model with a logit link function, using glm in R. For the family data we fitted a linear mixed model on the 0–1 scale using GCTA, as was done for the quantitative traits, and transformed the variance components to a liability scale (as implemented in GCTA), assuming a population prevalence of 15.54%, which is the prevalence in the entire MCPS population with T2D data (*n* = 125,042). We also fitted a generalized linear mixed model using glmer in R (lme4 package). To facilitate model convergence, age was rescaled to have a mean of 0 and an s.d. of 1. However, this implementation can only fit a single random effect with a simple covariance structure and therefore we fitted family as the only random effect.

Effect estimates on the linear (0–1) scale are therefore on the scale of prevalence, which aids interpretation. To approximate predicted prevalence of T2D in 100% IAM from the logistic multiple regression analysis on the population sample, we used $p(x)=1/(1+{e}^{-(\alpha +\beta x)})$, where p(x) is the probability of disease given IAM ancestry proportion x, β is the estimated coefficient from the logistic regression and α is estimated as $\alpha \approx \mathrm{logit}(K)-\beta \bar{x}$, with K the prevalence of T2D in the sample. This provides an approximation of p(1)−p(0), the counterfactual contrast of disease prevalence in 100% versus 0% IAM genome-wide ancestry proportions. Alternatively, a first-order approximation to the marginal effect on the prevalence scale is given by ${\beta }_{\mathrm{linear}}\approx \beta K(1-K)$, evaluated at the sample prevalence *K*.

### cTIA analysis

We estimated a cTIA for height and T2D for each person in the sample of 52,583 unrelated people. For each of these traits, we used results from trans-ancestry GWAS. For height we took the set of 12,111 SNPs from a conditional and joint (COJO) SNP analysis^32^. For T2D, we took 1,289 independent genome-wide significant variants from Suzuki and colleagues^33^. Height COJO variants and T2D GWAS variants were matched to MCPS genomes using previously TOPMED-imputed hard-call genotypes^4^ and, for each person, the cTIA was calculated by adding up the number of trait-increasing alleles, using the --score sum function in PLINK^61^ (v.1.9). The Ensembl Variant Effect Predictor^62^ tool was used to make annotations for the trait-associated SNPs of 12,111 COJO variants from Yengo and colleagues^32^ and the 1,289 independent genome-wide significant variants from Suzuki and colleagues^33^. Loss-of-function variants were defined as the variants with annotation frameshift, stop gained, stop lost, start lost, splice acceptor or splice donor^63^. A total of 314 (2 of 316 missing in the MCPS) missense and loss-of-function variants were identified for height-associated SNPs and 36 (6 of 42 missing in the MCPS) missense variants for T2D. To evaluate the association between cTIA and a specific ancestry while controlling for the effects of the other two ancestries, we conducted a partial correlation analysis using the ppcor^64^ package in R. To estimate the effect of ancestry after accounting for a cTIA we fitted the latter as a fixed effect in the mixed linear model analysis of the family data. For the T2D analysis, the cTIA was scaled to have a mean of 0 and an s.d. of 1. In the sensitivity analyses, we also fitted a cTIA associated with EA, including 2,925 COJO variants identified in people of European ancestry^65^.

### Selection analyses

We followed the approach of Guo and colleagues^37^. Allele frequencies for EUR ancestry were taken from the independent European population (*n* = 348,658) in the UK Biobank. Allele frequencies for IAM were calculated from a sample of 1,012 people in the MCPS who had an estimated genome-wide proportion of IAM ancestry greater than 99.5%. Allele frequencies were therefore estimated from people who were either of approximately 100% IAM ancestry or 100% EUR ancestry using imputed hard-call genotype data and were not affected by admixture or assortative mating within the MCPS. As both the GWAS for height and T2D were from predominantly EUR samples, we used European samples (*n* = 503) of the 1KG reference to calculate LD scores, applying the --ld-score and --ld-score-adj functions in GCTA^60^ with a window size of 1,000 kb. SNPs from the GWAS summary statistics^32,33^ were matched with those having EUR frequency, IAM frequency and LD score, resulting in 1,183,521 and 8,447,340 variants for height and T2D, respectively. The fTIA in IAM and EUR was calculated for the trait-associated 12,111 (11,778 available) COJO variants from Yengo and colleagues^32^ and the 1,289 (1,208 available) genome-wide significant variants from Suzuki and colleagues^33^. *F*_ST_ for each of the associated variants was calculated from Hudson’s *F*_ST_ equation^66^:

$${F}_{{\rm{st}}}=[({\mathop{p}\limits^{ \sim }}_{1}-{\mathop{p}\limits^{ \sim }}_{2}{)}^{2}-({\mathop{p}\limits^{ \sim }}_{1}(1-{\mathop{p}\limits^{ \sim }}_{1})/({n}_{1}-1))-({\mathop{p}\limits^{ \sim }}_{2}(1-{\mathop{p}\limits^{ \sim }}_{2})/({n}_{2}-1))]/[{\mathop{p}\limits^{ \sim }}_{1}(1-{\mathop{p}\limits^{ \sim }}_{2})+{\mathop{p}\limits^{ \sim }}_{2}(1-{\mathop{p}\limits^{ \sim }}_{1})]$$

where ${\mathop{p}\limits^{ \sim }}_{1}$ is the estimated fTIA in IAM, ${\mathop{p}\limits^{ \sim }}_{2}$ the estimated fTIA in EUR and *n*_i_ the sample size. To generate a null distribution, control SNPs matched on fTIA in EUR and LD score were sampled from the GWAS summary statistics (excluding the trait-associated variants), and their fTIA were calculated in IAM and EUR^37^. This was repeated 10,000 times, both for the analysis of *F*_ST_ and the analysis of the difference in trait-increasing alleles.

To correlate fTIA differences with effect sizes for the genome-wide trait-associated variants, we multiplied the square of the estimated effect size in the GWAS with expected heterozygosity (2 × fTIA × (1 − fTIA)) in EUR (Supplementary Fig. 10).

For the association between SNP rs200342067 and height, we performed linear regression analyses in the set of 52,583 unrelated people, adjusting for ancestries, age, age^2^, sex and district.

### Comparison of estimates from the population and family data

To explore concordance of the estimated ancestry effects from the family and population datasets, we used the estimated between and within-family effect sizes from the family data, predicted the population effect size (Supplementary Note 2) and then compared the predicted values with the ‘observed’ estimate from the independent population sample. The predicted value is a linear combination of the between and within-family effect size and we derived its sampling variance (and s.e.) using the reml-est-fix-varcov function in GCTA, which provides the full variance–covariance matrix of the fixed effect estimates from the mixed model analysis.

### Ethics statement

Ethics approval was obtained from the Mexican Ministry of Health, the Mexican National Council for Science and Technology and the University of Oxford, UK. All participants provided written informed consent.

### Reporting summary

Further information on research design is available in the Nature Portfolio Reporting Summary linked to this article.

## Online content

Any methods, additional references, Nature Portfolio reporting summaries, source data, extended data, supplementary information, acknowledgements, peer review information; details of author contributions and competing interests; and statements of data and code availability are available at https://doi.org/10.1038/s41586-026-11039-9.

## Supplementary information

Supplementary Information Supplementary Methods, including Supplementary Notes 1 and 2 and Supplementary Figs. 1–11.

Reporting Summary

Supplementary Tables Supplementary Tables 1–13.

Peer Review File

## Source data

Source Data Fig. 2

Source Data Fig. 3

Source Data Fig. 4

Source Data Extended Data Fig. 1

Source Data Extended Data Fig. 2

Source Data Extended Data Fig. 3

## Figures and Tables

**Extended Data Table 1:**
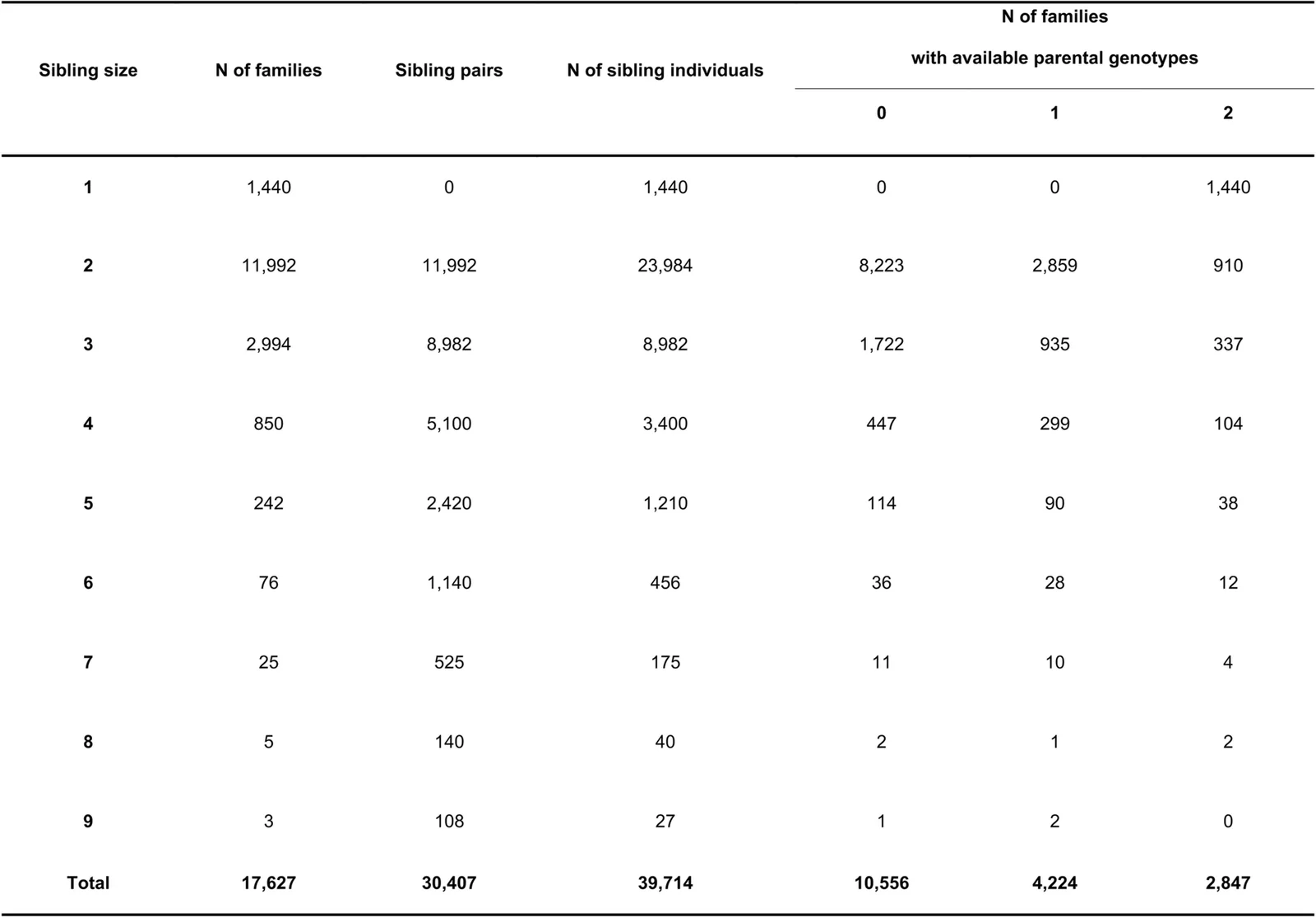
Distribution of family sizes

**Extended Data Table 2:**
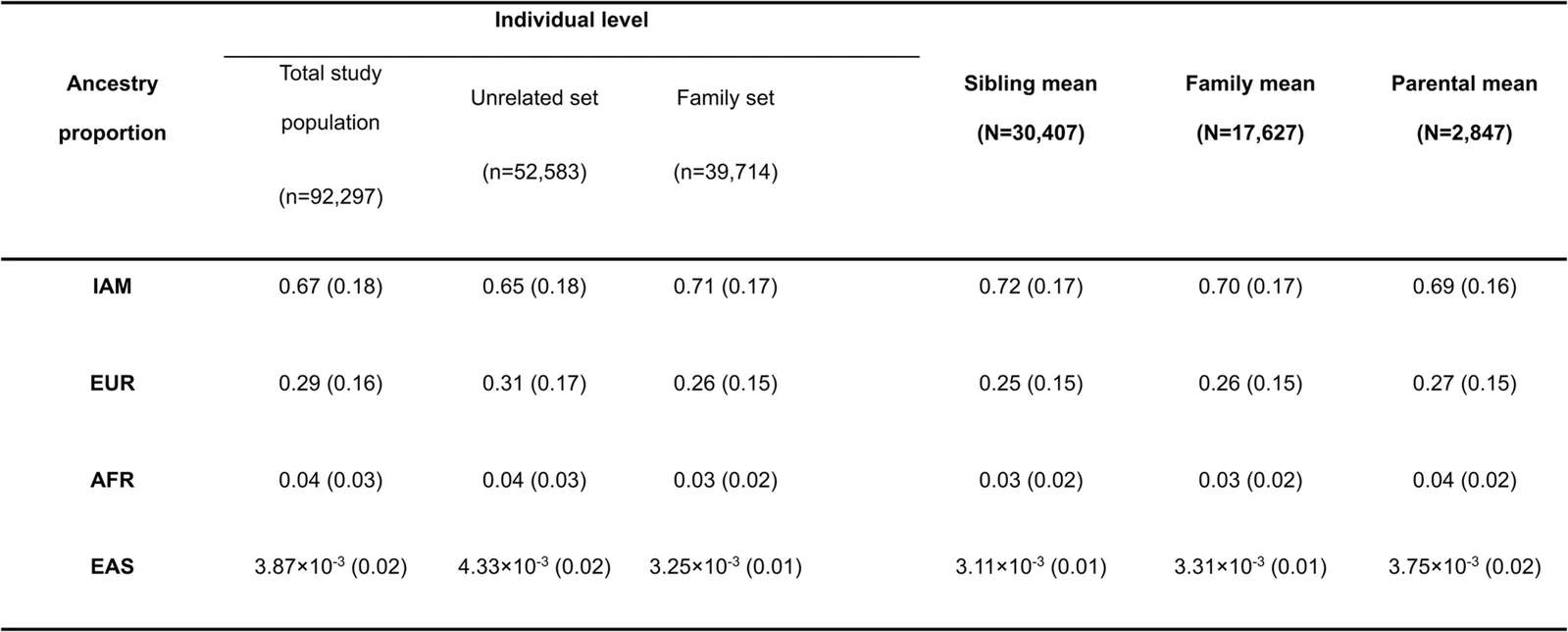
Ancestry proportions (mean [SD]) of individuals, sibling pairs, families, and parent pairs

**Extended Data Table 3:**
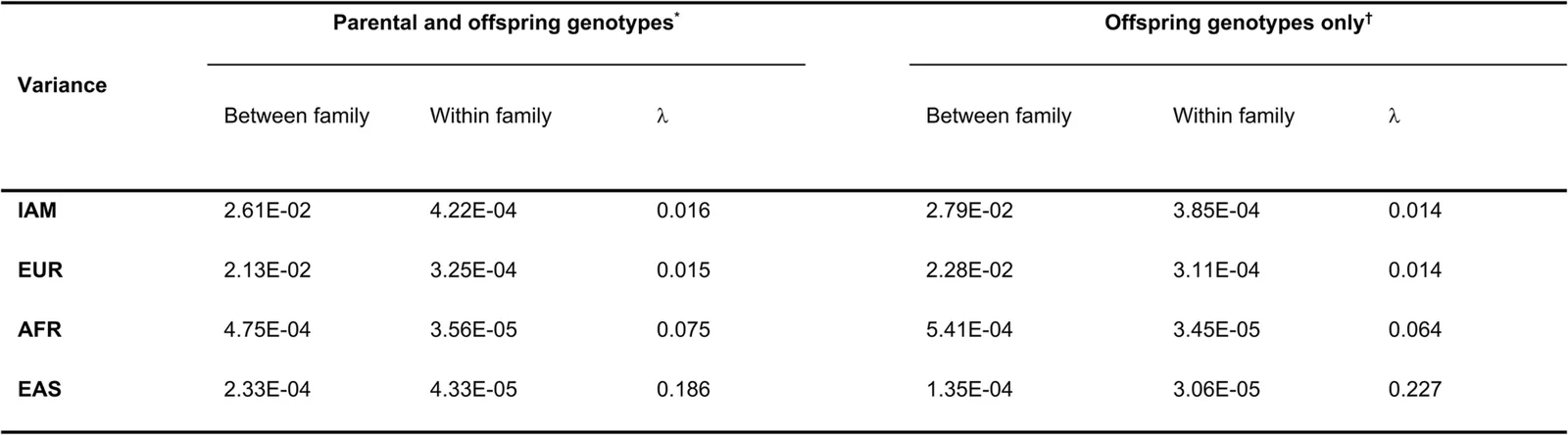
Between-family and within-family variance of ancestry proportions

^*^Results show the estimates of the between-family and within-family variance of ancestry proportions. For the 2,847 families with parental genotypes, the between-family variance is the variance of the parental mean, and the within-family variance is the residual variance from a regression of ancestry proportion on the parental mean. ^†^Using all 16,187 families with at least two genotyped offspring, the estimates of between and within-family variance are from a linear mixed model with family as a random effect. Parameter λ is the ratio of the within to between variance (Supplementary Note 1). For the prediction results (Eq(2) in Supplementary Note 1), the values of λ from the larger sample size were used.

**Extended Data Table 4:**
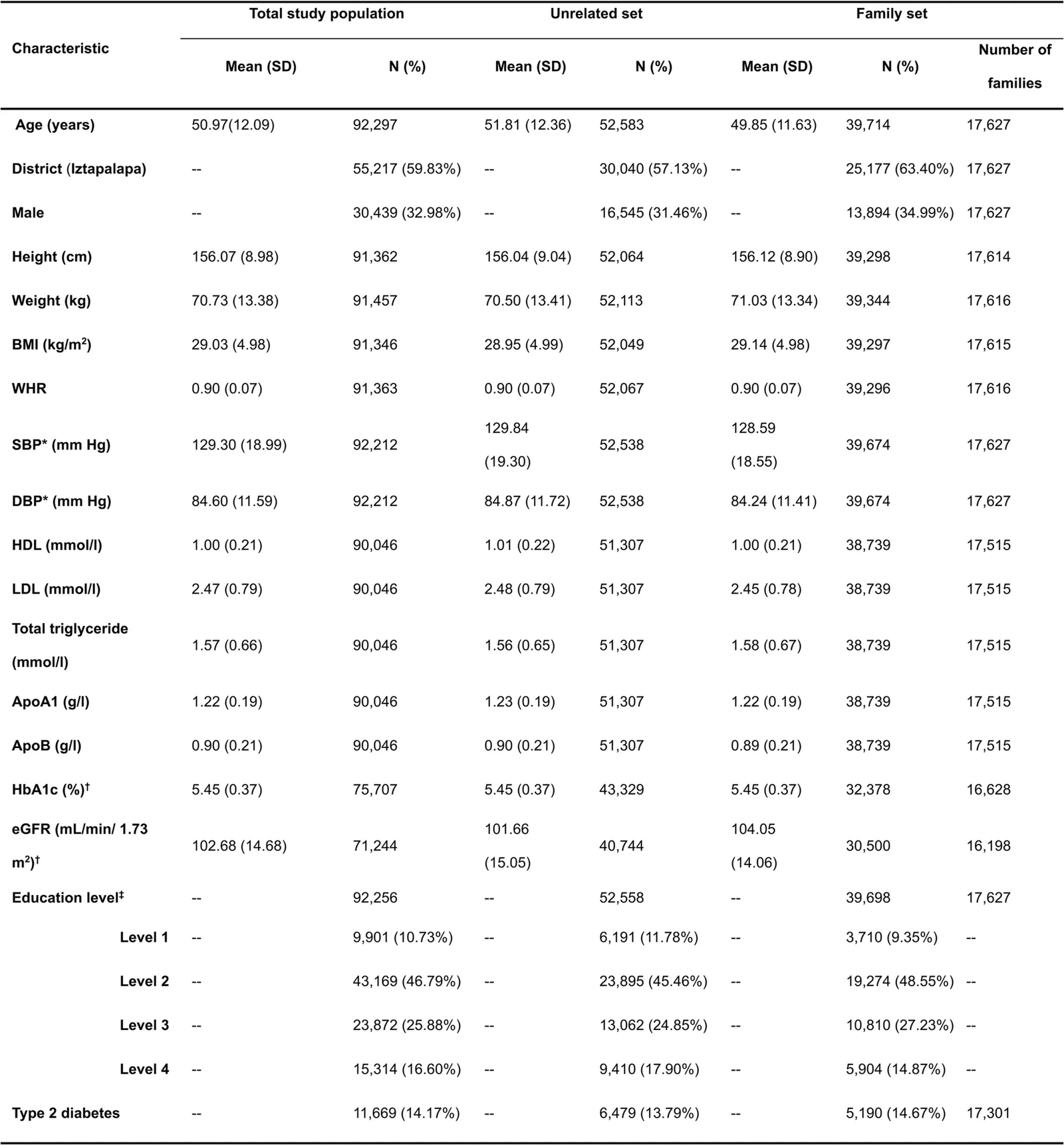
Baseline characteristics of study population

Continuous variables are presented as mean ± SD and Type 2 diabetes as number of cases (percentage). SD = Standard deviation, N = Number, BMI = Body mass index, WHR = Waist-to-hip ratio, SBP* = Systolic blood pressure adjusted for hypertension medication, DBP* = Diastolic blood pressure adjusted for hypertension medication, HDL = High-density lipoprotein cholesterol, LDL = Low-density lipoprotein cholesterol. ^†^Within non-diabetic population. ^‡^Educational level (4 = university or high school, 3 = middle school, 2 = elementary, 1 = illiterate or literate).

## Data Availability

Data from the MCPS, including the GSAv.2 genotype array dataset^4^ used in this study, are available to bona fide researchers upon application. The MCPS Data and Sample Sharing policy is available in English and Spanish at https://www.ctsu.ox.ac.uk/research/mcps and metadata describing the available datasets can be accessed through the MCPS Data Showcase (https://datashare.ndph.ox.ac.uk/mexico/). This study also used individual-level data from the UK Biobank (project ID 116122). UK Biobank data are available through application to the UK Biobank (https://www.ukbiobank.ac.uk/). Source data are provided with this paper.

## Code availability

The code used in this study is available at GitHub (http://github.com/mcps-analysts/within-family-effect-of-ancestry-on-complex-traits-in-a-Mexican-population) and Code Ocean (https://doi.org/10.24433/CO.5164456.v1)^67^.
