# Supplementary material for: Within-family effect of ancestry on complex traits in a Mexican population
Source: Nature. 2026 Sep 9;659(8139):133–40. doi: 10.1038/s41586-026-11039-9 (PMC7619614; doi:10.1038/s41586-026-11039-9)
Supplement: Supplementary file 1 [file 41586_2026_11039_MOESM1_ESM.pdf]

nature portfolio

https://doi.org/10.1038/s41586-026-11039-9

Supplementary information

Within-family effect of ancestry on complex traits in a Mexican population

In the format provided by the
authors and unedited

SUPPLEMENTARY INFORMATION

Supplementary Methods

Supplementary Note 1: Models to estimate ancestry effects between and within families

This supplementary note provides a derivation of the expectation of regression coefficients to estimate between and within family ancestry effects on complex traits in family and population data and gives details of the models that were applied to the MCPS data. The objective is to clarify the interpretation of regression coefficients within the context of genetic ancestry studies in populations and families.

Notation

To keep the notation simple, we initially assume admixture of only two ancestries whose genome-wide proportions within individuals sum to 1.0. We focus on the ancestry proportion (π) of one of the ancestries, so that the proportion of the unmodelled other ancestry is 1- π, and consider multiple admixed families with sibship size *n*. Define:

π_i: True parental average ancestry component of family *i*

π_{ij}: Ancestry of offspring *j* in family *i*

$d_{ij} = \pi_{ij} - \pi_i$: Deviation of offspring ancestry from the parental mean

$\hat{\pi}_i = \sum_j^n \pi_{ij} / n$: Mean sibship ancestry component in family *i*

By definition, $\hat{\pi}_i = \pi_i + e_i, with\ Var(e_i) = \sigma_{e_i}^2 = \frac{\sigma_d^2}{n}$.

Generating and analysis models

Assume the following generating model:

$$E(y_{ij}|\pi_i, \pi_{ij}) = \alpha\pi_i + \beta\pi_{ij} = (\alpha + \beta)\pi_i + \beta d_{ij}, \quad [1]$$

where α represents a between-family effect that is associated with ancestry and β represents the within-family genetic ancestry effect. If π_i is observed, then multiple linear regression of y_{ij} on π_i and π_{ij} will give unbiased estimates of parameters α and β because the model of analysis is the same as the assumed generating model. When π_i is unobserved due to the unavailability of parental genotypes for offspring with phenotypic data, the sibship average ancestry proportion is used instead and the analysis model becomes: $y_{ij} = b_B\hat{\pi}_i + b_W\pi_{ij} + \epsilon_{ij}$.

To derive the expectations of ordinary least-squares estimates of b_B and b_W we need the relevant variance and covariance terms:

$$\sigma^2_{\hat{\pi}_i} = \sigma^2_{\pi_i} + \frac{\sigma^2_d}{n}$$

$$\sigma^2_{\pi_{ij}} = \sigma^2_{\pi_i} + \sigma^2_d$$

$$cov(\hat{\pi}_i, \pi_{ij}) = \sigma^2_{\pi_i} + \frac{\sigma^2_d}{n} = \sigma^2_{\hat{\pi}_i}$$

$$cov(y_{ij}, \hat{\pi}_i) = (\alpha + \beta)\sigma^2_{\pi_i} + \beta\frac{\sigma^2_d}{n}$$

$$cov(y_{ij}, \pi_{ij}) = (\alpha + \beta)\sigma^2_{\pi_i} + \beta\sigma^2_d$$

Solving for b_B and b_W using these (co)variance components gives:

$E(b_B) = \alpha n/(n + \lambda)$, where $\lambda = \sigma^2_d / \sigma^2_{\pi_i}$, and $E(b_W) = \beta$.

Hence, when parental genotypes are not observed, the estimated between-family effect is shrunk towards zero, depending on sibship size *n* and variance ratio λ. This shrinkage reflects

the uncertainty of the true family mean ancestry component. The estimate of the within-family effect remains unbiased.

In the absence of family data, when the analysis is performed at the population level, the model simplifies to: $y = b_P\pi + e$, resulting in the regression coefficient: $E(b_P) = \beta + \alpha/(1 + \lambda)$. This expression demonstrates how the contribution of the between-family effect diminishes as λ increases, while the contribution of the within-family genetic effect β remains consistent. These results allow us to predict the expected value of b_P in a population sample from estimates of b_B and b_W in family data,

$$E(b_P | b_B, b_W) = b_B \frac{(n+\lambda)}{(1+\lambda)n} + b_W = cb_B + b_W, \quad [2]$$

with sampling variance

$$var(cb_B + b_W) = c^2 var(b_B) + var(b_W) + (2c)cov(b_B, b_W) \quad [3]$$

MCPS analyses

There are four ancestry groups in MCPS data. Therefore, each individual is assigned four genome-wide ancestry proportions, which sum to 1.0. There are only three estimable effects of ancestry, and we modelled the effects of IAM, AFR and EAS, so that the results are relative to a EUR ancestry baseline. For the analysis of unrelated samples, let $\pi_j^{(k)}$ be the genome-wide proportion of ancestry k of individual j. For each trait that we adjusted for age, age^2 and sex and standardised to a mean of 0 and a variance of 1, we fitted the model

$$y_j = \mu + \sum_{k=1}^{3} b_{P(k)}\pi_j^{(k)} + e_j, \quad [4]$$

where $b_{P(k)}$ are the effects associated with ancestry. For the binary trait type 2 diabetes, the linear part of the model is the same but y_j is replaced by logit($P(y_j = 1)$).

For the analysis of the family data, let $\pi_{ij}^{(k)}$ be the genome-wide proportion of ancestry k of individual *j* in family i. For each standardised trait *y*, we fitted the model

$$y_{ij} = \mu + \sum_{k=1}^{3}\left(b_{W(k)}\pi_{ij}^{(k)} + b_{B(k)}\bar{\pi}_{i}^{(k)}\right) + g_{ij} + c_i + e_{ij}, \qquad [5]$$

where $b_{W(k)}$ and $b_{B(k)}$ are the within-family and between-family effects of ancestry *k*, $\bar{\pi}_{i}^{(k)}$ is the average genome-wide proportion of ancestry k in family i, g_{ij} is the genetic value of individual j in family i and c_j is a family effect for family i. For $\bar{\pi}_{i}^{(k)}$ we took the average parental estimates if available and otherwise used the sibship average ancestry proportion. Sibling covariance for individual *j* and *l* in family *i* was modelled as,

$$cov(y_{ij}, y_{il}) = \gamma_{jl}\sigma_g^2 + \sigma_c^2,$$

with γ_{jl} the proportion of the genome shared identical-by-descent. For the generalised linear mixed model, y_{ij} is replaced by logit($P(y_{ij} = 1)$) and only a single random family effect c_i was fitted to account of the covariance between siblings.

In addition to estimation the population effects $b_{P(k)}$ directly in the unrelated set, we also predicted their expected value from the estimates of $b_{B(k)}$ and $b_{W(k)}$ obtained from the linear mixed model for each ancestry, using λ values from Extended Data Table 3 and the average sibship size for *n*. Standard errors of the predicted values were calculated from Eq[3],

using the sampling (co)variance of the regression coefficients from the option *--reml-est-fix-varcov* in GCTA.

Note that in the MCPS data, the within-family ancestry segregation variance σ_d^2 is very small relative to the between-family variance $\sigma_{\pi_i}^2$ (Extended Data Table 3), so that $\lambda \approx 0$, $E(b_B) \approx \alpha$ and $E(b_P) \approx \alpha + \beta$, the sum of the between and within-family effects.

Supplementary Note 2: Statistical power to detect a within-family ancestry effect

Statistical power to detect a within-family ancestry effect depends on the effect size and its sampling variance, the latter being a function of the sample size and the within-family variance in ancestry proportions. We consider the simplest case of two ancestries and *N* independent families each with 2 siblings. Let ρ be the sibling phenotypic correlation and consider the regression $y_{i1} - y_{i2} = b_W(\pi_{i1} - \pi_{i2}) + \epsilon_{i1} - \epsilon_{i2}$, with var($\epsilon_{i1} - \epsilon_{i2}$) ≈ 2(1-ρ) = var($y_{i1} - y_{i2}$). The expectation of $\hat{b}_W$, the ordinary least squares estimator of b_W, is E($\hat{b}_W$) = β and its sampling variance is, approximately, var($\hat{b}_W$) = 2(1- ρ) / ($N2\sigma_d^2$) = (1- ρ) / ($N\sigma_d^2$). The non-centrality-parameter (NCP) of a χ^2 test of $\beta = 0$ is NCP = $\beta^2/\text{var}(\hat{b}_W)$ = $N\sigma_d^2\beta^2/(1-\rho)$. To calculate statistical power, an experimenter has to define the false positive rate (α_0), the value of β , the number of sibling pairs *N*, sibling correlation ρ and the within-family variance in ancestry proportion σ_d^2. If we define $F(x,\kappa)$ as the cumulative density function of a χ^2 distribution with 1 degree of freedom with NCP equal to κ, and $Q(p)$ as the quantile function of a central χ^2 distribution with 1 degree of freedom and probability *p*, then the power of detection (Power) is

$$\text{Power} = 1 - F(Q(1-\alpha_0), N\sigma_d^2\beta^2/(1-\rho))$$

In the software R, power can be calculated as

```
Power <- function(N, rho, sigma2d, beta, alpha) {1 -
pchisq(qchisq(1-alpha, 1), 1, N * sigma2d * beta^2 / (1-rho))}
```

with rho = ρ, sigma2d = σ_d^2, beta = β and alpha = α_0.

We illustrate the power with the example of height and the IAM ancestry proportion, where $\rho = 0.41$, $N = 29{,}796$ and $\sigma_d^2 = 3.85\times10^{-4}$. The predicted SE of the estimate of b_W is $\sqrt{[(1-\rho) / (N\sigma_d^2)]} = 0.23$ and the power to detect an effect size of, for example, $\beta = 0.5$ standard deviations is 0.60 at $\alpha_0 = 0.05$. We note that the SE we report for the analysis of height from the full family analysis is 0.26. The main reason for this discrepancy is that we fit additional ancestries in the model and those are correlated with IAM ancestry, which increasing sampling variance. In particular, the AFR ancestry has a correlation of -0.51 with IAM ancestry. The effect of fitting AFR ancestry is to increase the sampling variance by a factor of $1/(1- (-0.51)^2) = 1.35$, and decrease the NCP by the same proportion, leading to a predicted sampling variance of 0.26, as observed from the mixed linear model analysis (Supplementary Table 4).

In real data applications there can be variation in family size and, as in our study, both a between- and within-family ancestry effect can be fitted. In addition, as in our study, parental genotypes may be available on only a subset of sibships. These factors will influence statistical power somewhat, but the essential information that determines statistical power is the total number of sibling pairs, the sibling correlation, and the variance of the difference in ancestry proportion between siblings.

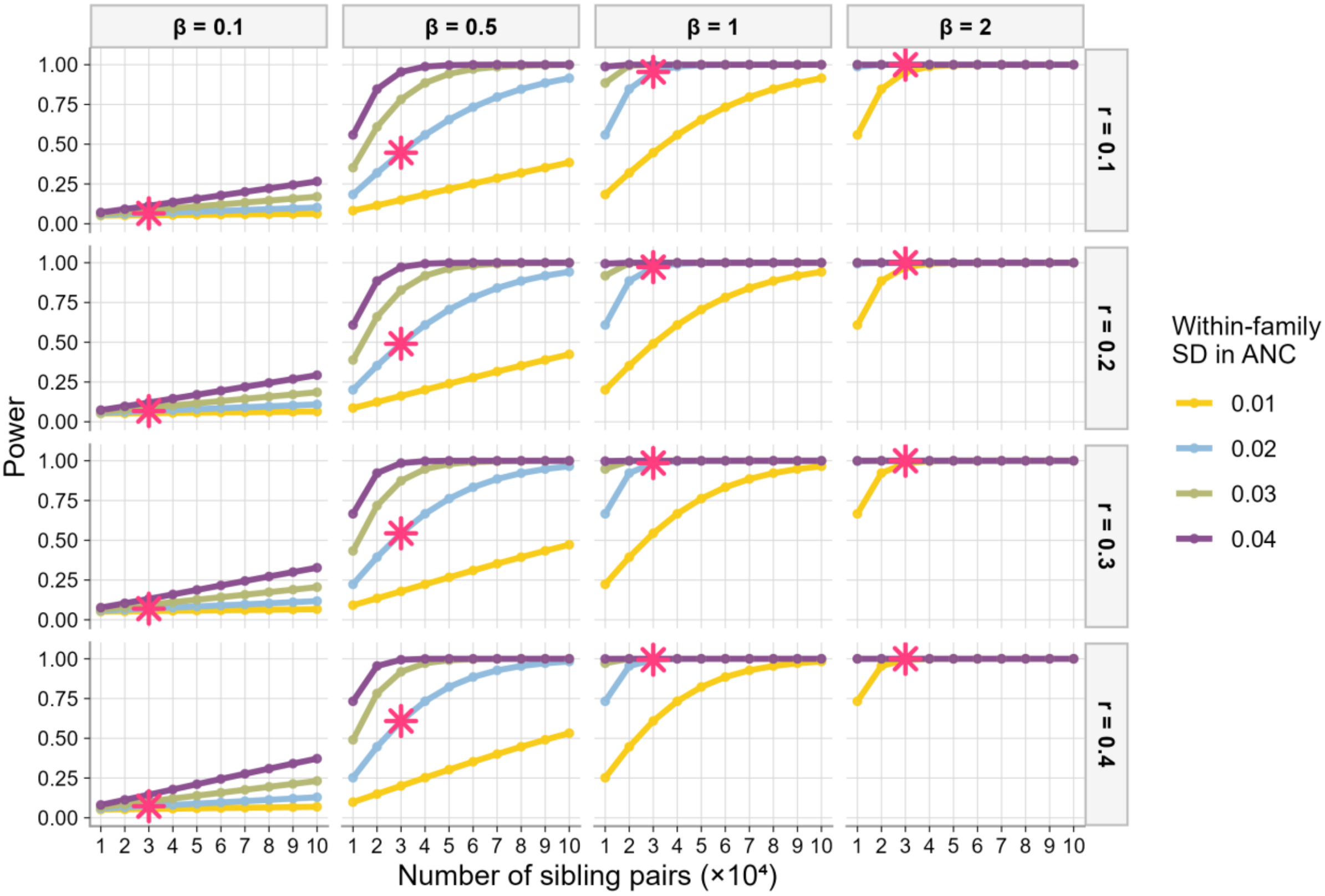

Supplementary Note Figure. Statistical power to detect a within-family ancestry effect.

Statistical power (at a type-I error rate of 0.05) to detect a within-family ancestry effect in a population admixed with two ancestries for a given number of sibling pairs (10,000 to 100,000), sibling phenotypic correlation (0.1 to 0.4), and within-family standard deviation in ancestry proportion (0.01 to 0.04). We have indicated the approximate power of the MCPS study with a red star corresponding to 30,000 sibling pairs and a within-family SD of 0.02. Effect size β is expressed in population standard deviation units.

Supplementary Figures

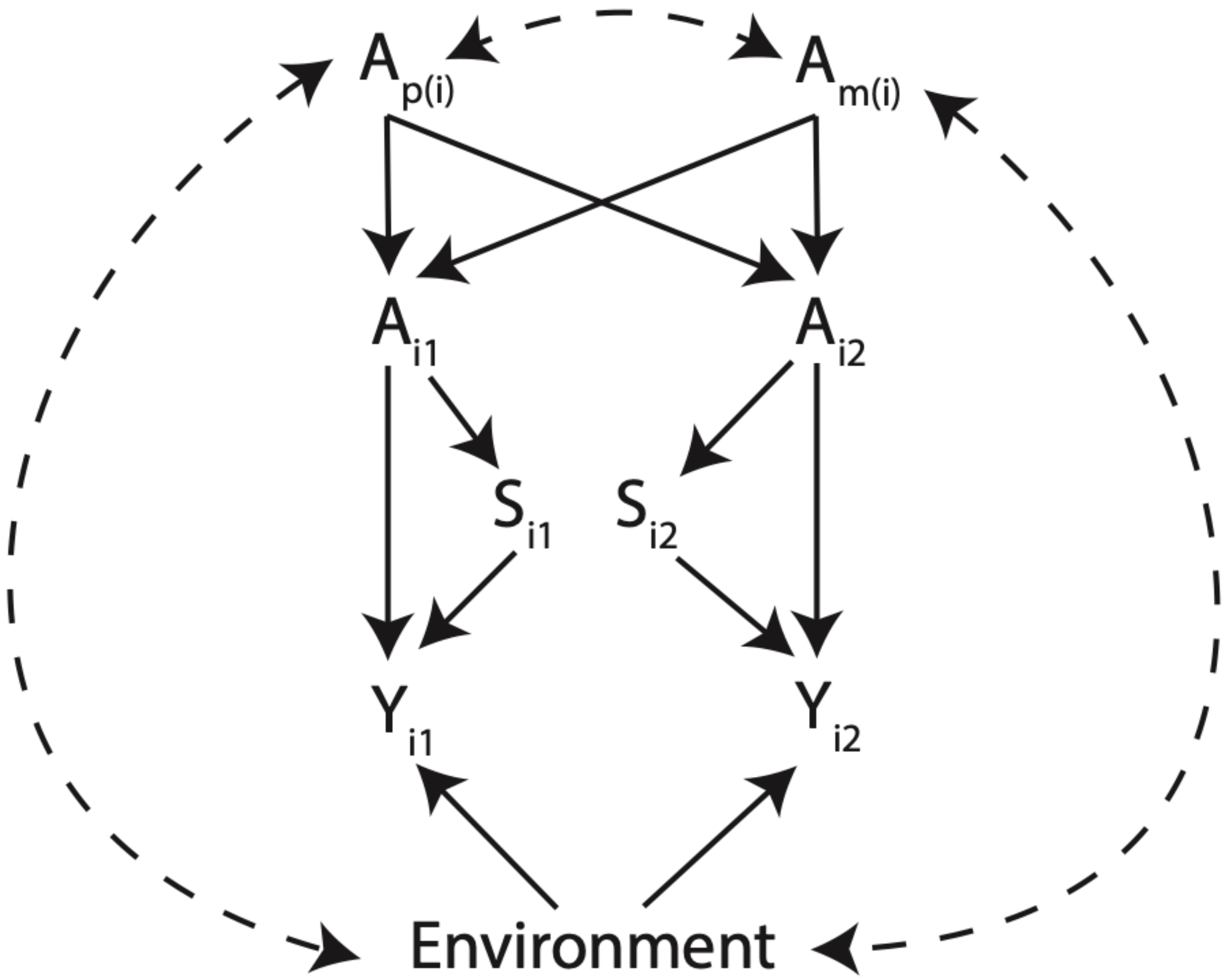

Supplementary Figure 1. Causal paths that lead to an association between ancestry (A) and a trait (Y) at the population and family level.

The black single-headed arrows represent causal effects, and the dashed double-headed arrows are correlations (not necessarily causal). $A_{p(i)}$ and $A_{m(i)}$ are the ancestries of the father and mother in family i. A_{i1} and A_{i2} are the ancestries of two siblings in family i. This shows that the sibling ancestries are conditionally independent of the environment given the parental ancestries (implying that their difference is also independent). This also shows that the within-family ancestry effect of A_{ij} on Y_{ij} can 'directly' affect the trait through a mechanism such as proximal trait biology (A → Y) or be mediated through S_{ij}, the ancestry-induced social environment of sibling j in family i (A → S → Y), for example the effect of ancestry on skin tone, which induces social discrimination that affects the trait.

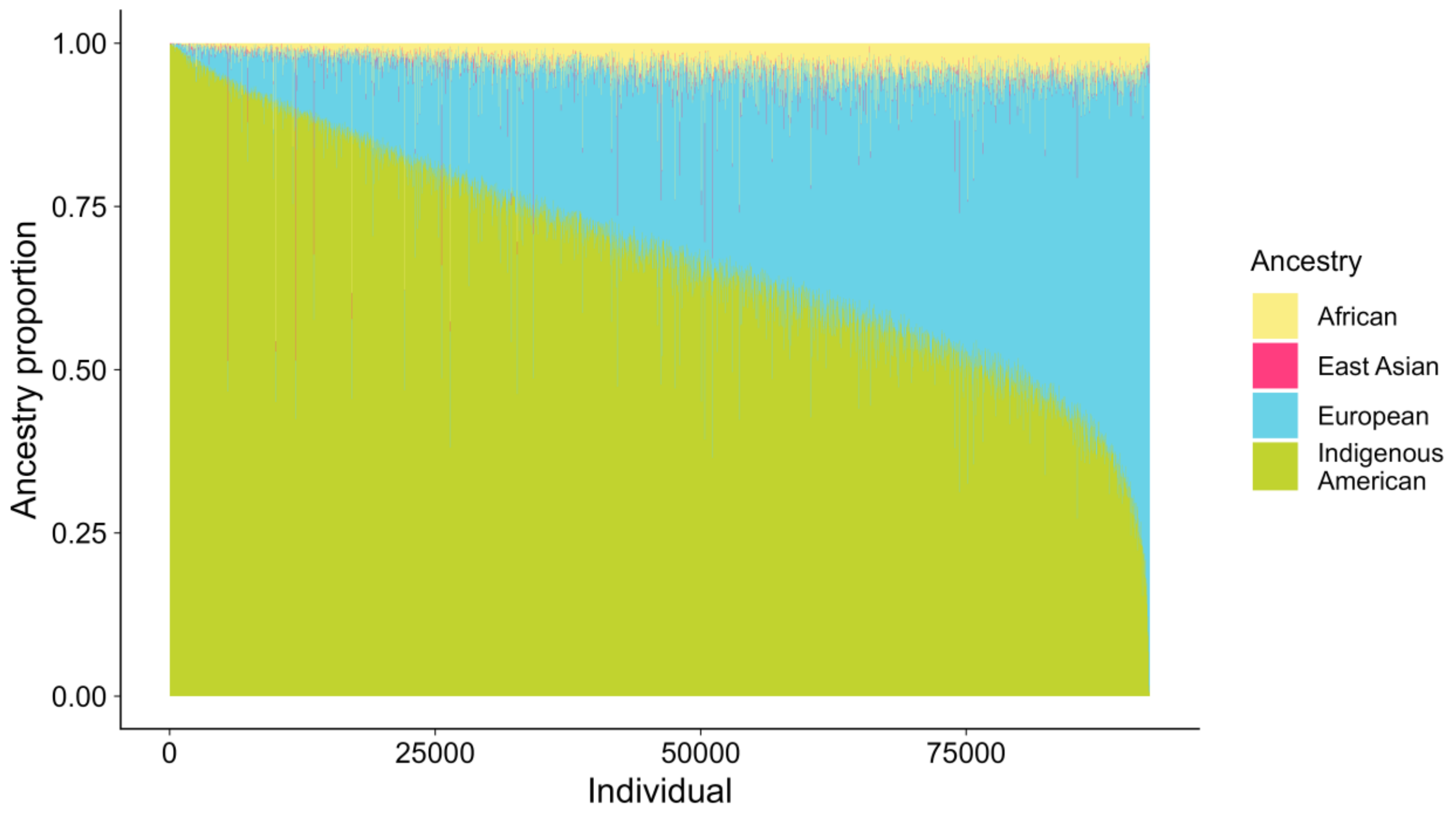

Supplementary Figure 2. Ancestry proportion distribution of the 92,297 study individuals.

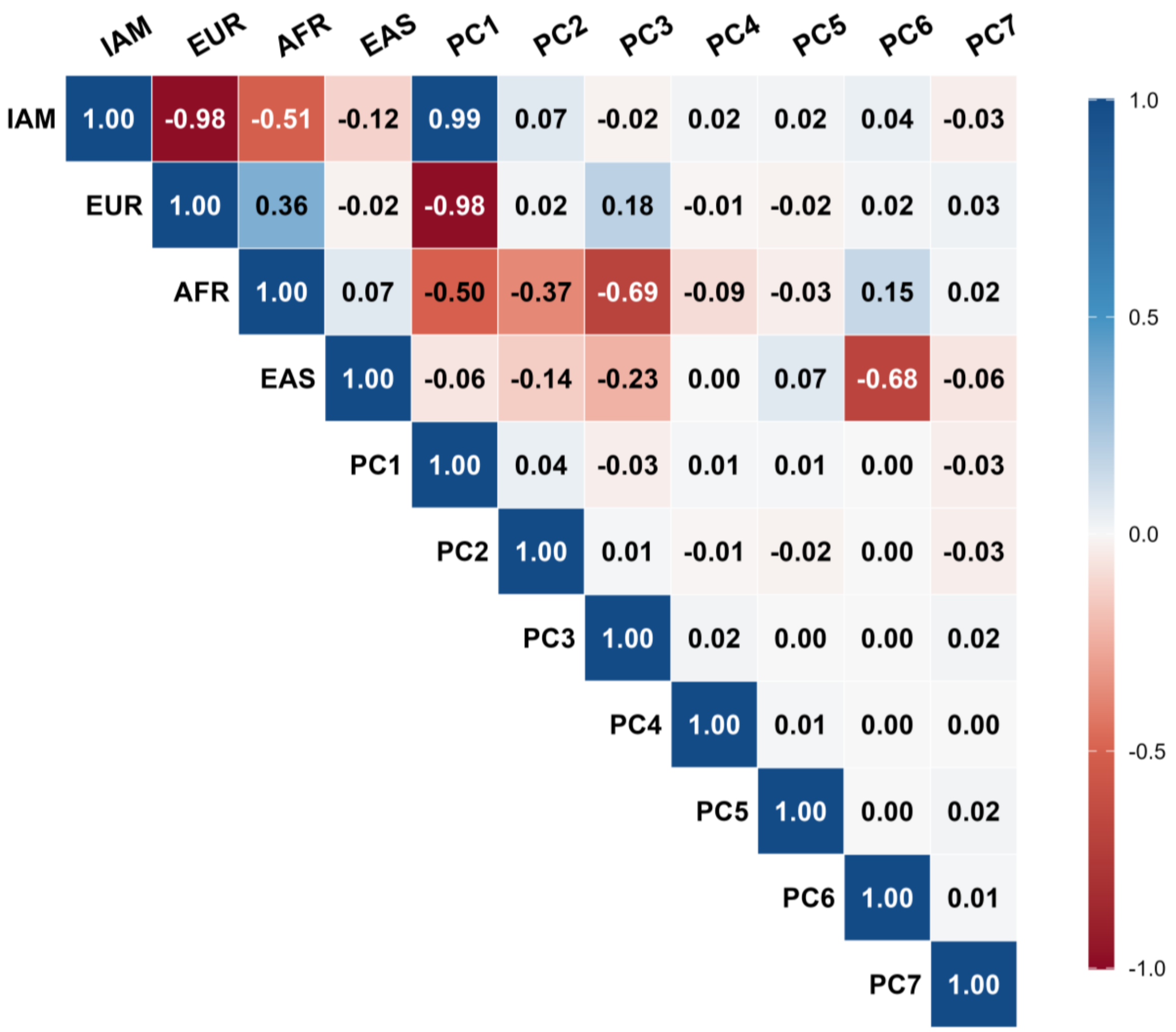

Supplementary Figure 3. Correlation plot of ancestry proportions and PCs in a sample of 52,583 unrelated individuals.

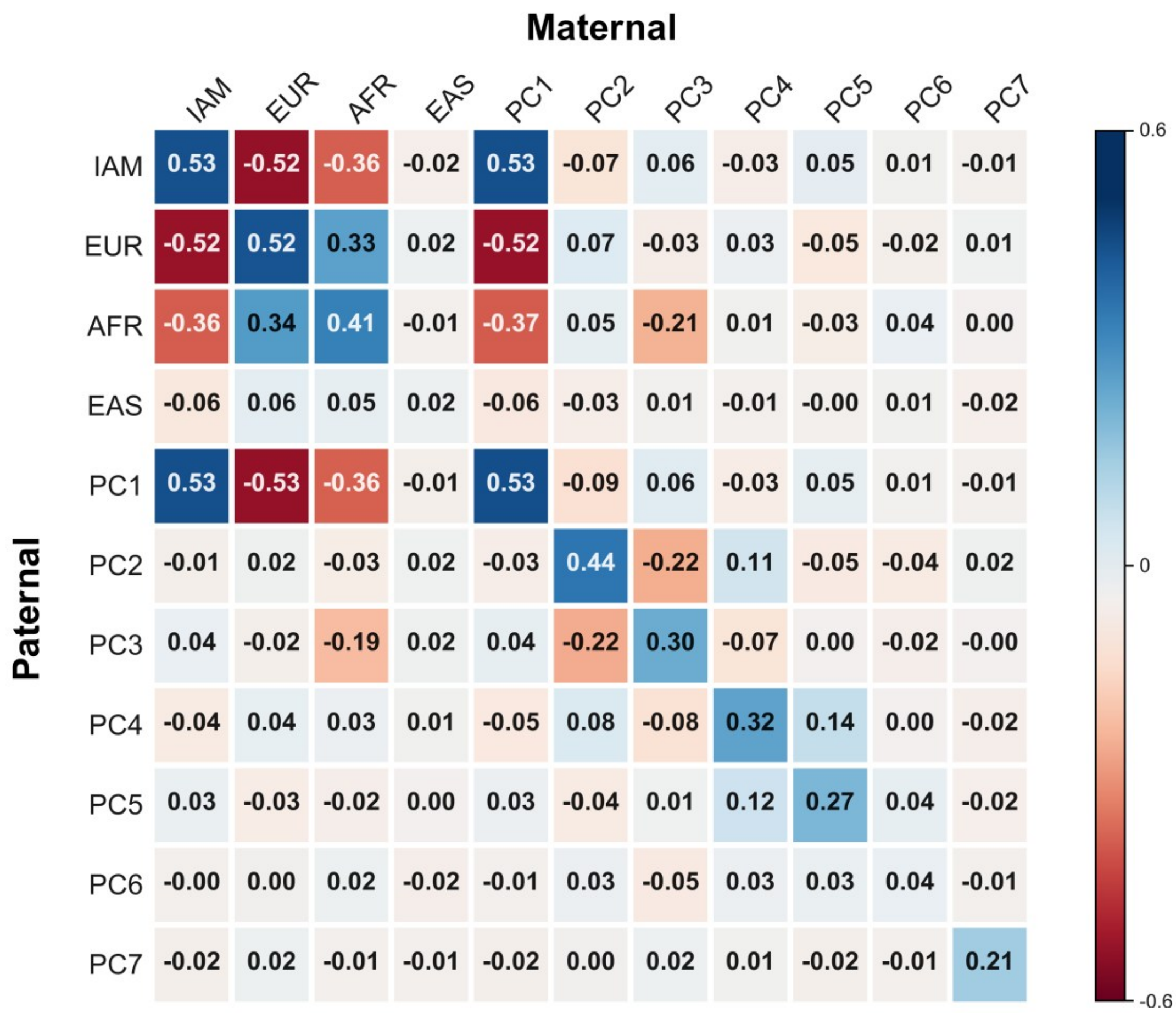

Supplementary Figure 4. Correlations between paternal and maternal ancestry proportions and PCs observed from 2,847 parent pairs.

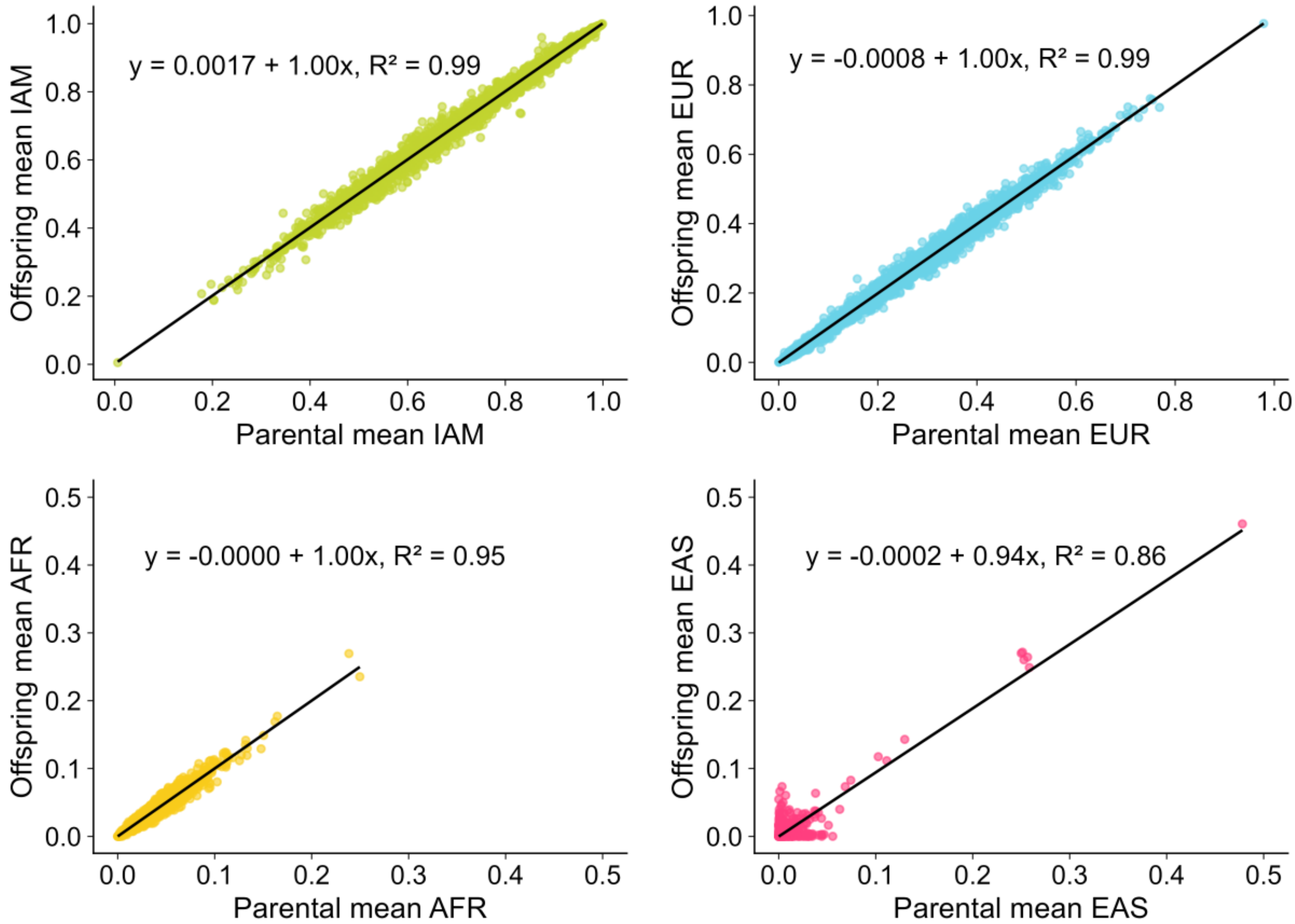

Supplementary Figure 5. Regression of offspring average ancestry proportion on the parental average ancestry proportion.

Correlations were observed in families (*n* = 2,847) with available paternal and maternal genotype data.

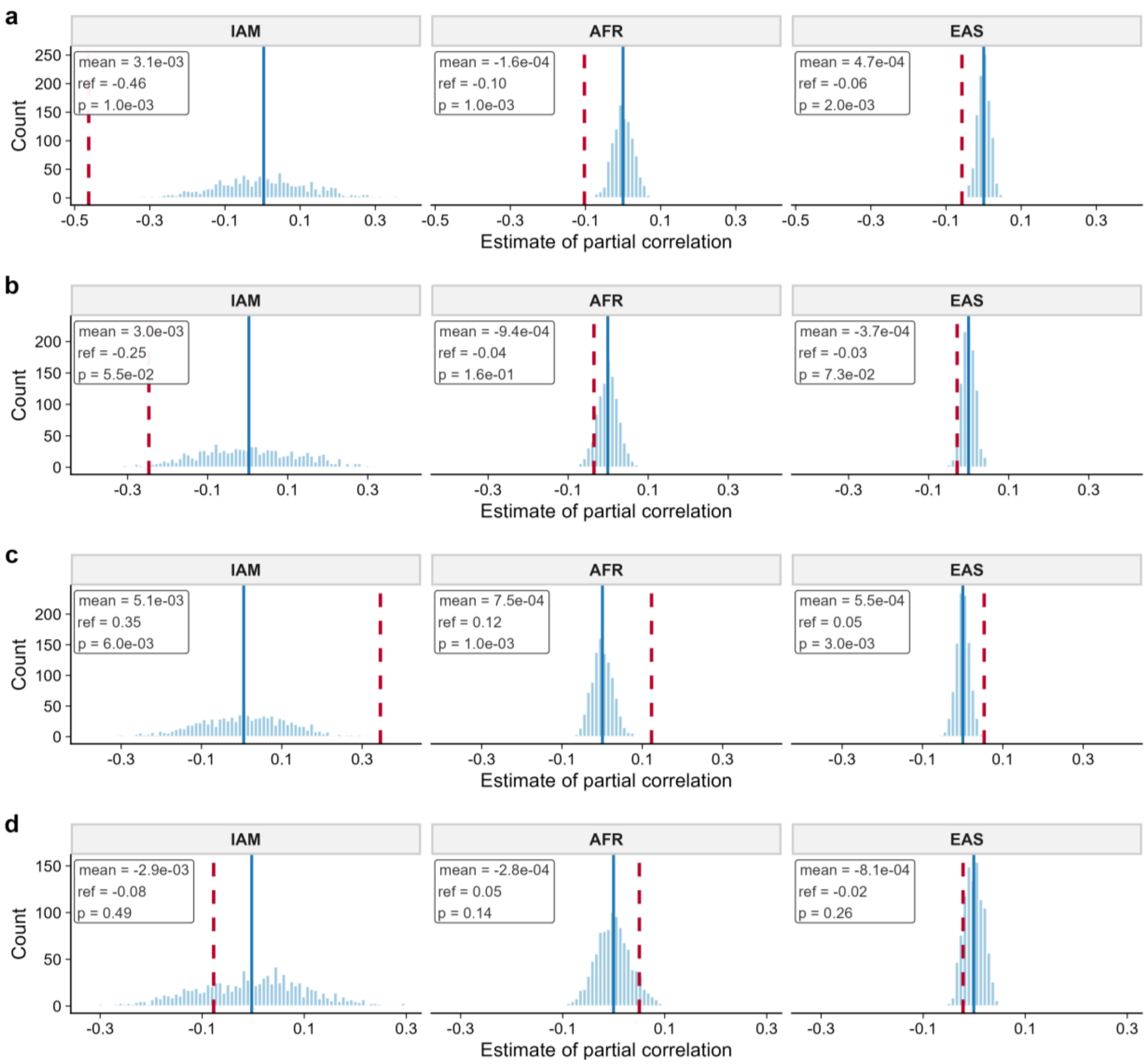

Supplementary Figure 6. Partial correlation between ancestry and the sign-permuted allele count across (a) 12k height-associated variants (b) 314 height-associated LoF and missense variants (c) 1k T2D-associated variants (d) 36 T2D-associated missense variants. Histograms show the empirical distributions of partial correlation coefficients between ancestry and sign-permuted allele count obtained from 1,000 permutations. In each iteration, the sign of the trait-increasing allele at associated loci was randomly permuted to generate a null distribution. Blue solid lines indicate the mean of the null distribution, while red dashed lines denote the observed partial correlation coefficients (reference value) between ancestry and trait-increasing allele counts. Insets report the mean, reference value, and empirical p-value.

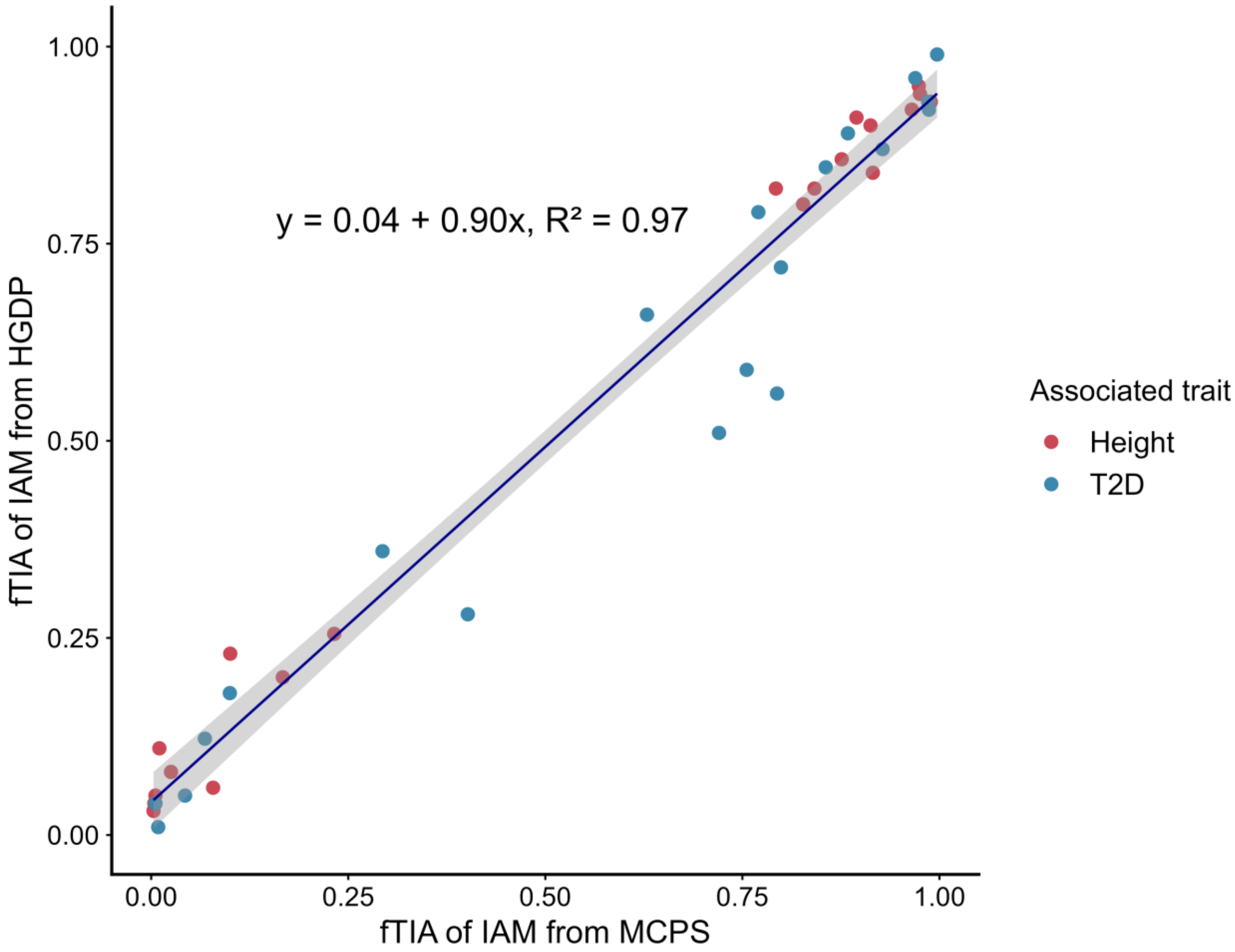

Supplementary Figure 7. Comparison of IAM allele frequencies in MCPS and HGDP at the top 20 loci with the largest EUR–IAM differences for height and type 2 diabetes separately.

The dark blue line represents the fitted linear regression, and the grey shaded area indicates the 95% confidence interval of the fitted regression line. Abbreviation: fTIA = frequency of trait-increasing alleles, IAM = Indigenous American, EUR = European.

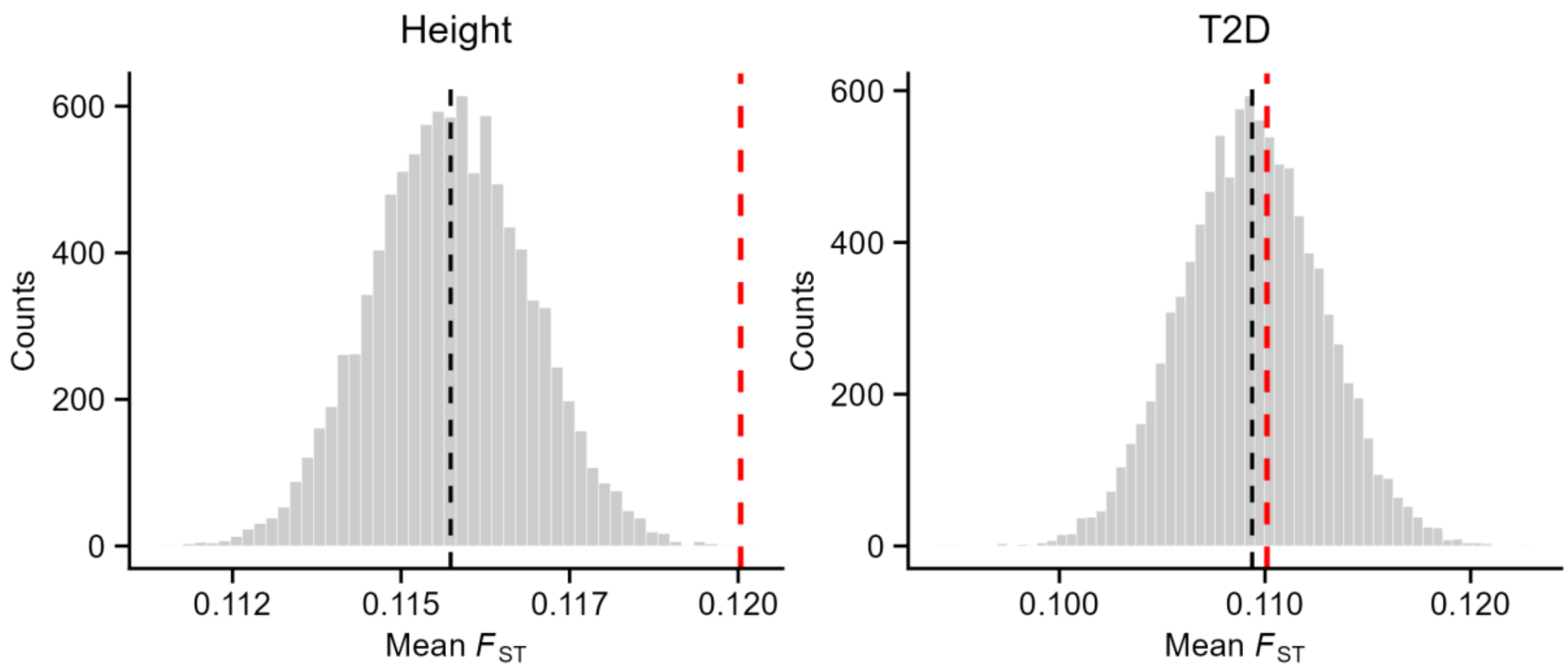

Supplementary Figure 8. Comparison of mean F_{ST} values between IAM and EUR for height and type 2 diabetes.

Histogram showing the null distribution of mean F_{ST} values derived from control SNP sets for height and type 2 diabetes, and the black dashed line indicates the mean value of the distribution. The red dashed line indicates the observed mean F_{ST} of the trait-associated SNPs.

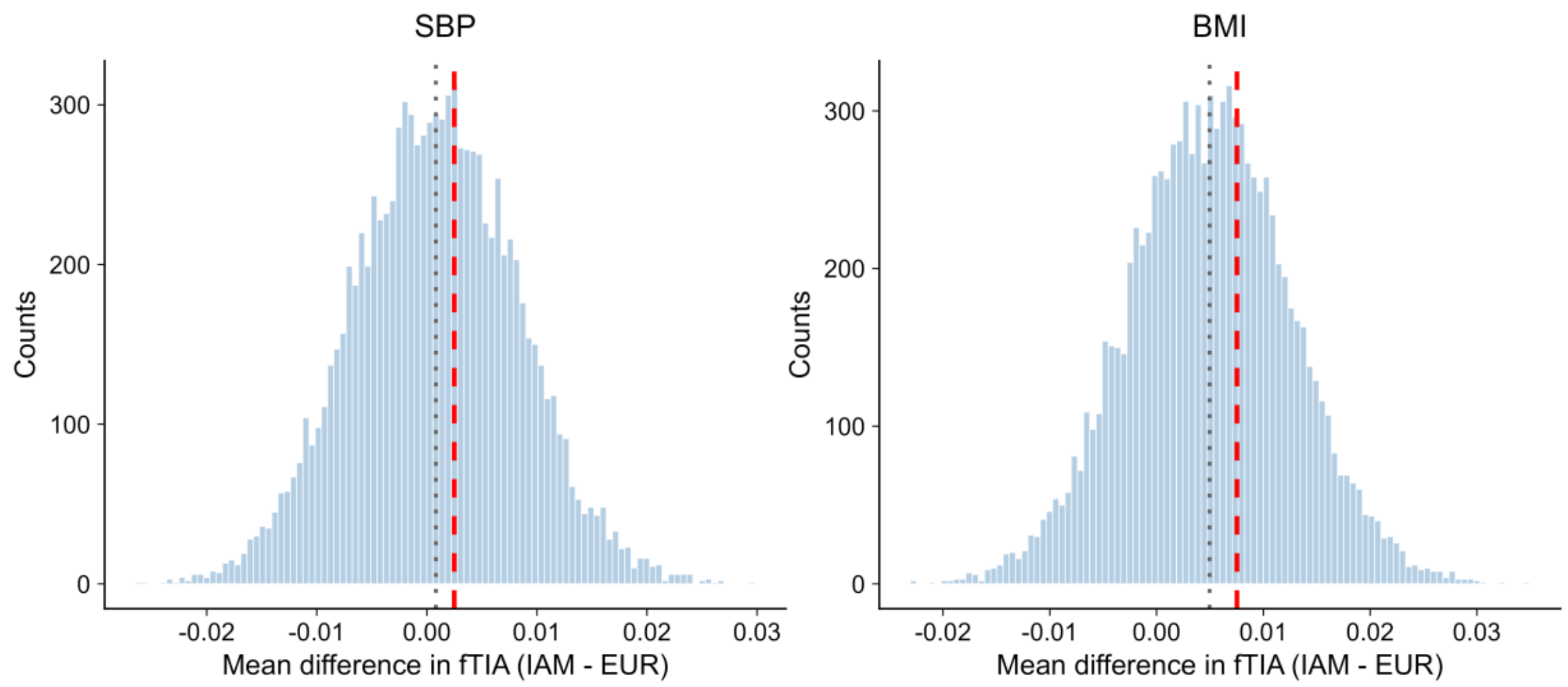

Supplementary Figure 9. Mean difference in the frequency of trait-increasing alleles between IAM and EUR populations for SBP and BMI.

The histogram shows the null distribution of mean fTIA differences from matched control sets, and the grey dashed line represents its mean value. The red dashed line indicates the mean value of the fTIA difference for the trait-associated SNPs.

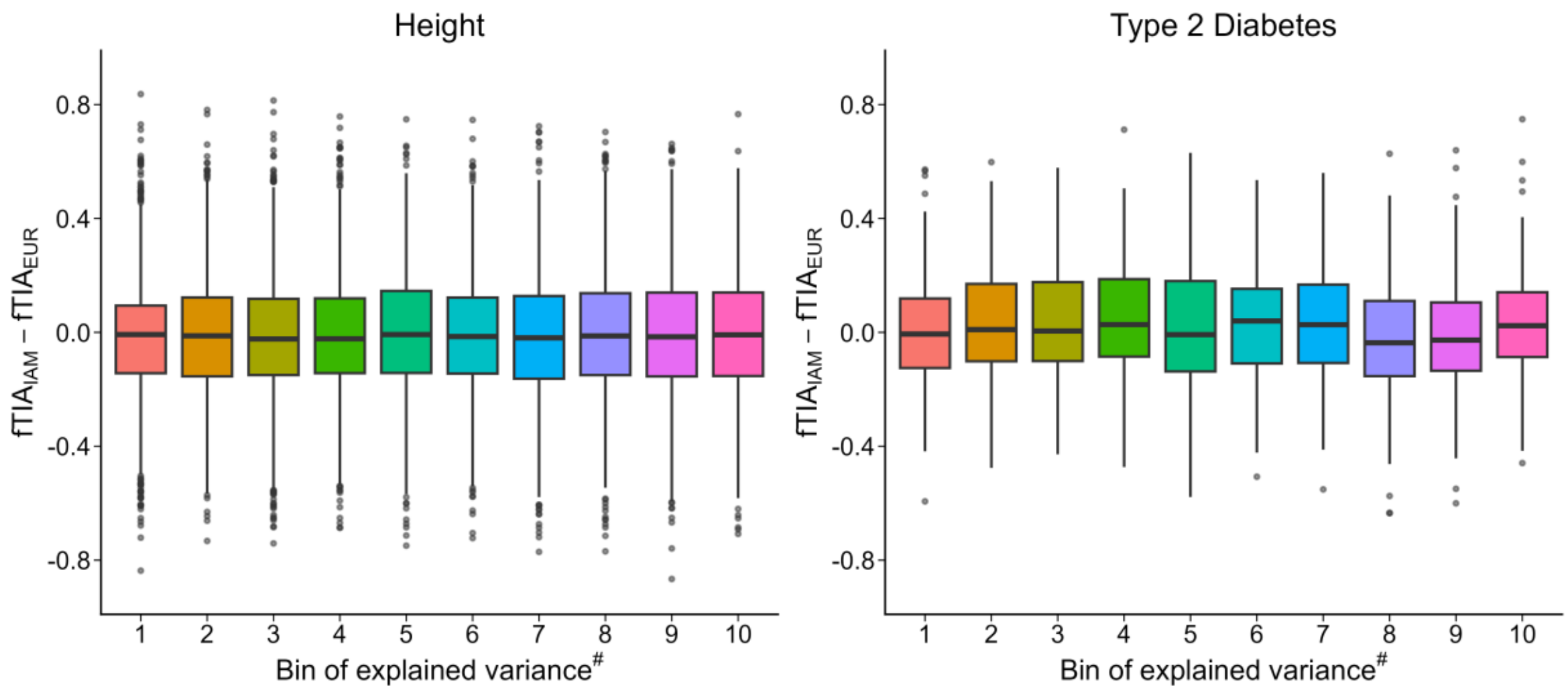

Supplementary Figure 10. fTIA differences against variance explained for 11,778 and 1,208 genome-wide significant variants of height and T2D, respectively.

The x-axis is the predicted variance# explained in EUR, assuming Hardy-Weinberg proportions of genotypes. The y-axis is the difference in trait-increasing allele frequency between IAM and EUR. #Explained Variance = $2 \times fTIA_{EUR} \times (1\text{-}fTIA_{EUR}) \times \beta^2$, where β is the estimated effect size from the discovery GWAS and $fTIA_{EUR}$ the frequency of trait-increasing alleles in the UK Biobank. Loci were grouped into 10 variance bins, with an equal number of loci per bin. For both height and T2D, there is no statistical association between increasing trait differentiation and predicted variance explained.

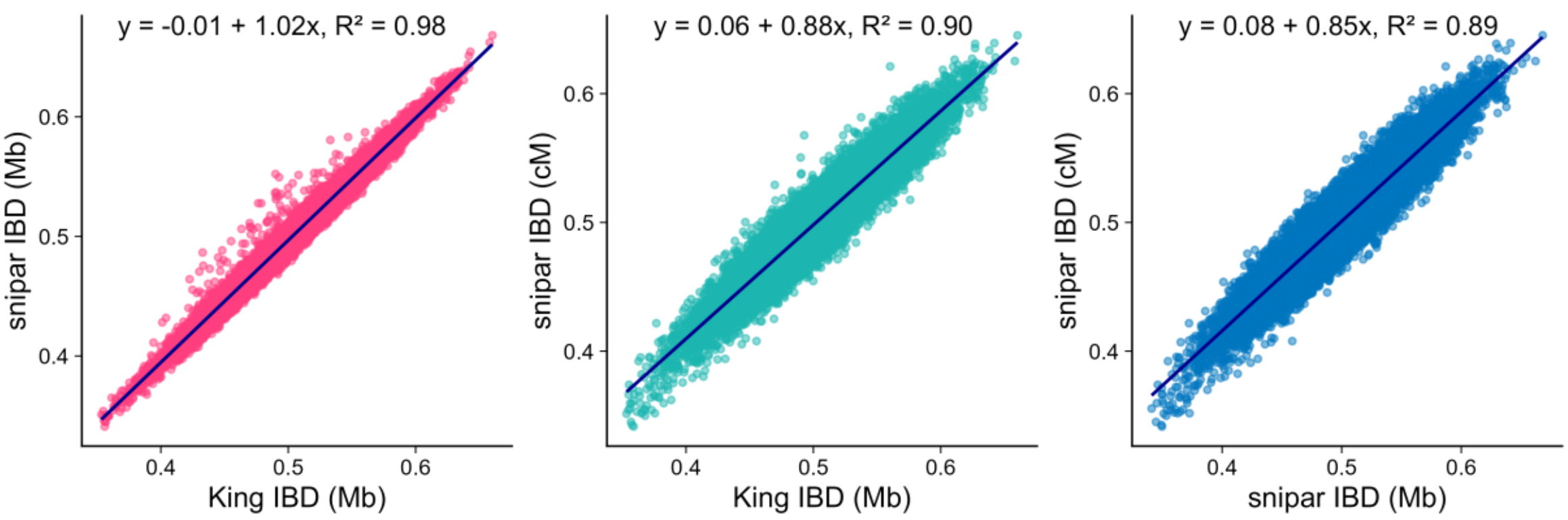

Supplementary Figure 11. Comparison between genome-wide IBD proportions calculated by KING and snipar from 30,407 full-sibling pairs.

Abbreviation: IBD = identical-by-descent, cM = centimorgan.
