# Supplementary material for: Within-family effect of ancestry on complex traits in a Mexican population
Source: Nature. 2026 Sep 9;659(8139):133–40. doi: 10.1038/s41586-026-11039-9 (PMC7619614; doi:10.1038/s41586-026-11039-9)
Supplement: Supplementary file 2 [file 41586_2026_11039_MOESM2_ESM.pdf]

nature portfolio

Corresponding author(s): Siqi Wang, Jason M. Torres, Peter M Visscher

Last updated by author(s): Aug 3, 2026

Reporting Summary

Nature Portfolio wishes to improve the reproducibility of the work that we publish. This form provides structure for consistency and transparency in reporting. For further information on Nature Portfolio policies, see our Editorial Policies and the Editorial Policy Checklist.

Statistics

For all statistical analyses, confirm that the following items are present in the figure legend, table legend, main text, or Methods section.

n/a	Confirmed	
☐	☒	The exact sample size (*n*) for each experimental group/condition, given as a discrete number and unit of measurement
☐	☒	A statement on whether measurements were taken from distinct samples or whether the same sample was measured repeatedly
☐	☒	The statistical test(s) used AND whether they are one- or two-sided<br>*Only common tests should be described solely by name; describe more complex techniques in the Methods section.*
☐	☒	A description of all covariates tested
☐	☒	A description of any assumptions or corrections, such as tests of normality and adjustment for multiple comparisons
☐	☒	A full description of the statistical parameters including central tendency (e.g. means) or other basic estimates (e.g. regression coefficient) AND variation (e.g. standard deviation) or associated estimates of uncertainty (e.g. confidence intervals)
☐	☒	For null hypothesis testing, the test statistic (e.g. *F*, *t*, *r*) with confidence intervals, effect sizes, degrees of freedom and *P* value noted<br>*Give P values as exact values whenever suitable.*
☒	☐	For Bayesian analysis, information on the choice of priors and Markov chain Monte Carlo settings
☐	☒	For hierarchical and complex designs, identification of the appropriate level for tests and full reporting of outcomes
☐	☒	Estimates of effect sizes (e.g. Cohen's *d*, Pearson's *r*), indicating how they were calculated

Our web collection on statistics for biologists contains articles on many of the points above.

Software and code

Policy information about availability of computer code

Data collection	No software was used for data collection.
Data analysis	ADMIXTURE(v1.3.0) was used to estimate individual ancestry proportions.<br>KING was used for kinship estimation.<br>snipar was used to estimate genome-wide IBD.<br>Stata (v18.5) was used for phenotype data processing.<br>R (v4.3.2) was used to perform linear regression, logistic regression, generalised linear mixed model (R package lme4), partial correlation analyses (R package ppcor).<br>GCTA (v1.94.3) was used to perform REML analyses.<br>PLINK (v1.9) was used for genotype data processing and calculation.<br><br>The code used in this study is available from GitHub at https://github.com/mcps-analysts/Direct-effect-of-genetic-ancestry-on-complex-traits.

For manuscripts utilizing custom algorithms or software that are central to the research but not yet described in published literature, software must be made available to editors and reviewers. We strongly encourage code deposition in a community repository (e.g. GitHub). See the Nature Portfolio guidelines for submitting code & software for further information.

Data

Policy information about availability of data

All manuscripts must include a data availability statement. This statement should provide the following information, where applicable:

- Accession codes, unique identifiers, or web links for publicly available datasets
- A description of any restrictions on data availability
- For clinical datasets or third party data, please ensure that the statement adheres to our policy

Data from the Mexico City Prospective Study, including the GSAv2 genotype array dataset used in this study, are available to bona fide researchers upon application. The MCPS Data and Sample Sharing policy is available in English and Spanish at https://www.ctsu.ox.ac.uk/research/mcps, and metadata describing the available datasets can be accessed through the MCPS Data Showcase (https://datashare.ndph.ox.ac.uk/mexico/). This study also used individual-level data from the UK Biobank (project ID 116122). UK Biobank data are available through application to the UK Biobank (https://www.ukbiobank.ac.uk/).

Human research participants

Policy information about studies involving human research participants and Sex and Gender in Research.

Reporting on sex and gender	All participants were included in the analyses irrespective of sex or gender. Sex was accounted for by including it as a covariate in regression models or by standardising phenotypes within each sex group before analysis, as appropriate.<br><br>The objective of this study was not to investigate sex-specific effects; therefore, sex-stratified analyses were not performed. The sex distribution of the study population is provided in Extended Data Table 4 as part of the baseline characteristics.
Population characteristics	We include 92,297 participants from MCPS in the current study; their mean age was 50.97(sd=12.09), 55,217 (59.83%) of them are from the Iztapalapa district.
Recruitment	The MCPS is a prospective cohort of over 150,000 adult participants from Mexico City (references). The baseline survey took place between April 14 1998 and September 28 2004 and focused on households in two urban districts—Coyoacán and Iztapalapa. Residents aged 35 or older were invited to participate in the study. (Tapia-Conyer, R. et al. Cohort Profile: The Mexico City Prospective Study. International Journal of Epidemiology 35, 243–249 (2006). Ziyatdinov, A. et al. Genotyping, sequencing and analysis of 140,000 adults from Mexico City. Nature 622, 784–793 (2023)).
Ethics oversight	Ethics approval was obtained from the Mexican Ministry of Health, the Mexican National Council for Science and Technology, and the University of Oxford, UK. All participants provided written informed consent.

Note that full information on the approval of the study protocol must also be provided in the manuscript.

Field-specific reporting

Please select the one below that is the best fit for your research. If you are not sure, read the appropriate sections before making your selection.

☒ Life sciences ☐ Behavioural & social sciences ☐ Ecological, evolutionary & environmental sciences

For a reference copy of the document with all sections, see nature.com/documents/nr-reporting-summary-flat.pdf

Life sciences study design

All studies must disclose on these points even when the disclosure is negative.

Sample size	This study included all eligible participants from the MCPS with available genetic and phenotypic data (52,583 unrelated individuals and 39,714 individuals from family sets). As this was an observational study based on an existing cohort, all eligible data were analysed. The adequacy of the sample size for detecting within-family ancestry effects was evaluated by statistical power analyses described in Supplementary Note 2.
Data exclusions	To ensure that all pairs within a family were full siblings according to KING's estimation, 25 individuals were removed. Additionally, 4 individuals were excluded to ensure that each family had no more than two putative parents. Phenotype outliers were excluded if height (cm) < 120 or > 200, weight (kg) < 35 or > 250, BMI (kg/m2) < 15 or > 60, and WHR < 0.5 or > 1.5. The continuous traits were adjusted for age and age2 and standardised (mean 0, SD 1) within each sex, and outliers were further excluded according to mean ± 5SD.
Replication	N/A -- Rationale: This study analysed existing population-based cohort data rather than generating new experimental data. Therefore, independent replication through repeated data collection was not applicable.
Randomization	N/A -- Rationale: This study analysed existing population-based cohort data. No experimental interventions or treatments were assigned to participants; therefore, randomization was not applicable.

Blinding	N/A -- Rationale: This study analysed existing population-based cohort data. No experimental interventions or treatments were assigned to participants; therefore, blinding was not applicable.

Reporting for specific materials, systems and methods

We require information from authors about some types of materials, experimental systems and methods used in many studies. Here, indicate whether each material, system or method listed is relevant to your study. If you are not sure if a list item applies to your research, read the appropriate section before selecting a response.

Materials & experimental systems

n/a	Involved in the study
☒	☐ Antibodies
☒	☐ Eukaryotic cell lines
☒	☐ Palaeontology and archaeology
☒	☐ Animals and other organisms
☒	☐ Clinical data
☒	☐ Dual use research of concern

Methods

n/a	Involved in the study
☒	☐ ChIP-seq
☒	☐ Flow cytometry
☒	☐ MRI-based neuroimaging
