# Supplementary material for: Within-family effect of ancestry on complex traits in a Mexican population
Source: Nature. 2026 Sep 9;659(8139):133–40. doi: 10.1038/s41586-026-11039-9 (PMC7619614; doi:10.1038/s41586-026-11039-9)
Supplement: Supplementary file 3 [file 41586_2026_11039_MOESM4_ESM.pdf]

nature portfolio

Peer Review File

Within-family effect of ancestry on complex traits in a Mexican population

Corresponding Author: Professor Peter Visscher

Any redactions in this file are there to maintain patient confidentiality, the confidentiality of unpublished data, or to remove third-party material.

This file contains all reviewer reports in order by version, followed by all author rebuttals in order by version.

Version 0:

Reviewer comments:

Referee #1

(Remarks to the Author)
The authors present their work comparing unrelated individuals and nuclear families to disentangle the relationship between estimated genetic ancestry proportions and a selection of complex traits. Their results are compelling, with some supported by previous work and others being novel. I would like to state for the record that I am not against the topic entirely, only that it must be handled with care to avoid overinterpretation and misappropriation. Having a reference like this would be important for future work, both to dispel incorrect beliefs around race essentialism and eugenics and lay a groundwork for more within-family analyses. But it must be well-justified, presented within a specific context, and measured in its conclusions.My concerns are mostly with the framing of the work, including a lack of justification for the research question or selection of European ancestries as being the default when it is not the most common in this population, as well as the strong causal language when it is not justified. I expand on these concerns below with suggestions for the authors.

- The overall study question is not well justified. What do we have to gain from these results? Why is this question important? How does it influence the public health or precision medicine in these communities? More information on the background setting up this question is required. (This is a side note, but I would suggest not mentioning agriculture and dog breeds at the beginning of a paper intending to talk about admixed people.)
- There is a lack of acknowledgement throughout the manuscript about their structured population. Assortative mating in highly admixed populations in which classism and colorism are tightly linked is not unexpected. And yet the authors treat many analyses with a single pooled analysis without considering the geographic and socioeconomic structuring of the study population and recruitment area. Sensitivity analyses could address some of these concerns, but the larger point is that there is little information about the health of these patients within their communities.
- Overall, it would be beneficial to soften the language. These statistical tests cannot be used to establish causality, for while within-family analyses may control for differences in shared environment, those are not the only environmental differences that can matter. For example, what is described as a result of direct effects (within family analyses) could also be due to colorism, which is an environmental or social determinant of health, and can influence siblings differently within a family. The discussion states that environmental effects can only occur between families (lines 269-271), which is not true, especially in highly admixed populations in which a single family can have a wide range of skin tones. While a family may provide the opportunity for education consistently within their family, the effects of colorism (such as stress) are more likely to be felt for T2D. In the discussion, causality is mentioned and justified but there is no test of causality within the manuscript and you cannot conclude causality, just association and the suggestion of potential direct effects.
- The use of 100% EUR as the reference group goes against the recommendations of two NASEM reports on population descriptors in genetics and genomics (2023) and race/ethnicity in biomedical research (2024). I acknowledge that these are two US-based set of recommendations, but they may still be helpful for this manuscript in order to be consistent with newer guidelines and to minimize the potential for misuse of these results on a sensitive topic.
- If I were a general audience reader of this journal, I would not understand what you mean by direct genetic ancestry effects. Is it having over a certain proportion of IAM ancestry would therefore predispose you? Or is it just that you are more likely to have specific ancestry-specific haplotypes or variants? This distinction is incredibly important for the interpretation of results. Taking T2D as an example, there is nothing inherently “bad” about having IAM ancestry, just that having more IAM ancestry makes it more likely to have more variants that may increase risk. I realize this may seem like a minor distinction, but can

have an enormous effect on the reception and utilization of these results.

Specific analyses:
- The authors state in lines 204-207 that "if the polygenic score captures the direct ancestry effect", then you would see attenuation. However, it is difficult to conceptualize or make this direct point when (1) PGS can differ in distribution by ancestry because of trait differences AND (2) they can differ because of random drift unrelated to the trait but more a product of distance from the training dataset. It is therefore difficult to say with the language currently included in the manuscript that the attenuation of effects is because of anything trait-specific, or because the training PGS was from a predominantly EUR background group. This is consistent with a larger concern above regarding the softening of language, or allowing more nuance.
- How do you differentiate between selection and assortative mating, which has already been shown with ancestry proportions? It has already been shown to occur with height in other populations, so why not with this population? And if it is assortative mating, how does that interpretation differ from "natural selection", particularly in the interpretation of genetic determinism and racial essentialism?

Language choices:
In addition to agriculture and dog breeds, I would avoid calling an admixed population a "natural experiment" (line 253). I know it is used often and honestly is not on its own problematic, but in this context it could come across as offensive to this and other admixed populations.

(Remarks on code availability)

Referee #2

(Remarks to the Author)
Review of "Direct effect of genetic ancestry on complex traits in a Mexican population" by Wang et al. (2025), submitted to Nature

This paper estimates within- and between-phenotype associations between genetic ancestry and 15 complex phenotypes in a sample of admixed individuals from Mexico City. It reports evidence that Indigenous American (IAM) ancestry is associated, when comparing among unrelated individuals, with shorter stature, higher risk of Type 2 diabetes (T2D), and lower educational attainment (EA). However, when comparing within-families, IAM ancestry is not associated with EA, whereas associations with height and T2D persist. The within-family ancestry-phenotype association is interpreted as a causal effect of ancestry because of the randomness of within-family genetic segregation. The finding of a "direct" effect of ancestry on T2D and height is buttressed with results from analyses polygenic burden and selection. The major strengths of the study are (1) its focus on individuals with complex genetic ancestries, particularly on those with IAM ancestry who live in the global South, who are extremely underrepresented in genetics research generally, (2) its rigorous approach to estimating within-family associations, and (3) its integration of methods across medical, population, and evolutionary genetics. It addresses a question of long-standing, contentious interest: Why do populations differ in their trait averages? I anticipate that the paper will draw the attention of a broad readership.

My major criticism of the paper, which I think is readily ameliorable, is its characterization of the "direct" effect of ancestry. I agree with the authors that, because segregating genetic variance is randomly "assigned" at conception, the within-family association between genetic differences and phenotype differences can be interpreted as an estimate of the causal effect of the genetic differences tagged. As the authors describe, this causal effect is not confounded by dynastic environmental differences that are correlated with ancestry across generations and between-families, i.e., what behavioral geneticists would call "passive gene-environment correlation" (Plomin, deFries, & Loehlin, 1977).

This causal effect, however, cannot be equated with a purely "genetic" effect, as it still might depend on evocative or active gene-environment correlations. Sandy Jencks (1972) made this point conceptually with his "red-headed children" thought experiment. More recently, Dalton Conley and his colleagues made this point in their 2019 analysis of skin tone and hypertension ("New Evidence of Skin Color Bias and Health Outcomes Using Sibling Difference Models: A Research Note"). Their design was conceptually similar to the present manuscript, in that they analyzed sibling differences to estimate causal genetic effects on a complex medical trait. Using a sample of admixed black/Latinx Americans, they found that within-sibling differences in skin tone were associated with elevated rates of hypertension. However, they interpreted this association as evidence for colorism (social discrimination against darker skin tones). The hypothesized pathway, in other words, was gene --> proximal phenotype (skin tone) --> social environment (discrimination) --> distal phenotype (hypertension).

The authors acknowledge that genetic effects might have social mechanisms in the Discussion (p. 11), where they write: "While our within-family direct ancestry effect estimates can be given a causal interpretation, they do not imply a specific mechanism. It is possible that ancestry differences cause phenotype differences through social mechanisms such as ancestry-related appearance-based discrimination. However, our estimates are consistent with ancestry associated phenotype differences in height and T2D risk being entirely explained by direct ancestry effects (estimated within-family): if appearance related discrimination exerted an environmental effect on these phenotypes, it is likely this would also operate between families and induce a between-family ancestry association in our design, which we do not observe for height and

T2D."

I am not entirely convinced by this argument that a social mechanism connecting ancestry with health would necessarily operate as strongly between-families as within-families. Indeed, several scholars have argued that colorism operates as an intra-racial and intra-family social process (see, e.g., Lori Tharps, "Same Family, Different Colors: Confronting Colorism in America's Diverse Families"; Maria Abascal and Denia Garcia, "Pathways to Skin Color Stratification: The Role of Inherited (Dis)Advantage and Skin Color Discrimination in Labor Markets"; Jessica Kizer, "Skin Color Stratification and the Family: Sibling Differences and the Consequences for Inequality.") One could argue that these previous scholars have prematurely attributed within-family associations between skin tone and complex traits to social processes rather than to "inside the skin", biologically-mediated effects of genetic ancestry. My point, though, is that we don't really know, and this study doesn't offer strong evidence to resolve the question. There is not (yet) dispositive evidence about the specific mechanisms that connect genetic ancestry with complex phenotypes.

Given this lack of evidence about mechanism(s), and the potential misinterpretations of work connecting genetic ancestry to health outcomes, I think that the parameter the authors are estimating—-the causal effect of genetic ancestry on complex traits—-should be described with more care and caveat throughout the whole manuscript, not just in the Discussion. For example, in the Introduction, after the authors introduce the term "direct effects of ancestry", they should note that, as with direct genetic effects operating within-ancestries, "direct" effects can be mediated by culturally-specific gene-environment correlations. Similar caveating should continue through the Results and Discussion. I, personally, would avoid the term "direct" and just use "causal," being sure to emphasize that genetic causes can operate through social mechanisms ("transactional genetic effects" was the term used by Laurel Raffington and colleagues in "Polygenic Scores in Developmental Psychology").

My other major question about the paper concerns power. Given the high parental correlations for ancestry, the relatively small within-family variance in ancestry, and some reasonable priors about effect sizes, what is the sample size that would be necessary to reliably identify within-family effects of ancestry on these 15 traits, including on EA where the authors report a null association? How far below (or not below) that mark is the current sample? I anticipate that this report might open to door to many subsequent papers aiming to use admixed samples to investigate genetic contributions to between-group differences in complex traits, and giving the field more concrete guidance regarding power and sample size would be useful.

My final comment, which is more minor, concerns the term "shared environmental variance." The authors are initially clear (p. 7) that what they are estimating is the variance due to effects that are common to siblings but are not explained by IBD sharing, an estimate that might include genetic variance in the population due to assortative mating, but at other points they call this variance component "shared environmental variance." They don't have conclusive evidence that this variance is due to what would be commonly understood as environmentally transmitted effects rather than being due to assortative mating, so I suggest tweaking the language to reflect this ambiguity.

(Remarks on code availability)

Referee #3

(Remarks to the Author)
A. Summary of the key results

Wang et al. use data from the Mexico City Prospective Study (MCPS) to evaluate the contribution of genetic ancestry to complex phenotypes, leveraging the admixed nature of Mexican genomes. They estimate within-family ancestry effects across 15 traits and report significant direct effects of Indigenous American ancestry on height and type 2 diabetes. At the population level, strong associations are observed for educational attainment and several other traits; however, these do not persist once tested in family-based analyses. Despite this pattern being present across traits, the manuscript emphasizes educational attainment rather than presenting a balanced view of the broader set of null results.

B. Originality and significance

The study applies a within-family design to disentangle direct ancestry effects in an admixed population, which is uncomon. While the methodological framework itself is not entirely novel—building on established within-family regression approaches—the adaptation to global ancestry analyses in admixed cohorts represents a useful formalization.

C. Data & methodology

The MCPS dataset is large and well-suited for these analyses, particularly given the extensive family structure. The methodology is valid and clearly described, but there are important limitations. Analyses rely exclusively on global ancestry estimates; incorporating local ancestry mapping would considerably strengthen the findings, and the sample size is sufficient to support such analyses.

The definition of educational attainment based on categorical levels rather than years of schooling is problematic, as it reduces comparability across populations and weakens interpretability. Details on the choice of K in the ADMIXTURE analysis should also be added.

D. Appropriate use of statistics and treatment of uncertainties

The statistical models are appropriate, and the derivations clarifying the expectations of regression coefficients are carefully laid out.

However, the interpretation of the educational attainment results overlooks key confounding factors. Sensitivity analyses already show district of residence plays a role; this is expected given known demographic and educational differences between Iztapalapa and Coyoacán, including school quality and ancestry composition. This entanglement complicates interpretation and should be acknowledged more explicitly.

E. Conclusions: robustness, validity, reliability

The conclusions about height and type 2 diabetes are robust and supported by within-family analyses. By contrast, the conclusions regarding educational attainment are overstated, given the lack of direct ancestry effects and the methodological caveats noted. More caution is needed in communicating these results to avoid overinterpretation.

F. Suggested improvements

Present a balanced overview of all traits showing population-level associations but no significant direct ancestry effects, rather than emphasizing educational attainment alone.

Incorporate local ancestry mapping or admixture mapping analyses, which are feasible with the current sample size.

Provide justification for the ADMIXTURE K used, and report cross-validation results.

Expand secondary analyses beyond coding variants. The current claim that coding variants are “more likely to be causal” is misleading. Non-coding variants are central in complex disease through regulatory effects; they are simply harder to analyze, and lack of expertise should not be conflated with lack of relevance.

The authors could attempt to replicate their population-level findings for some traits using data from the Mexico Biobank. It is unclear why this was not pursued.

H. Clarity and context

The manuscript is generally well written and the statistical derivations are clearly presented. However, the abstract and conclusions currently give disproportionate weight to educational attainment, which could mislead readers. Greater balance in highlighting both significant and null findings would improve clarity and context.

(Remarks on code availability)
I verified that the code and documentation are available. However, I was unable to run the code on the platform. The documentation lacks a user manual for individuals not directly involved in the project. While the inline comments are helpful, a standalone manual would greatly facilitate broader usability.

Version 1:

Reviewer comments:

Referee #1

(Remarks to the Author)
I thank the authors for their revised manuscript, which greatly improved upon the messaging and clarify. My comments mainly revolve around the positioning of this work, including softening some language and explaining why this matters within the field. While the authors did respond within the “response to reviewers” document, much of this information is not adequately translated into test within the manuscript. While I realize some of my comments are repetitive with my prior comments, it is still worth stating and improving upon in the revised version. For what it’s worth, I found the response to reviewer comments much clearer than the manuscript for many of these points, so I hope it’s just a matter of changing language/framing. I’ve organized my comments below in terms of major comments, minor comments, and figure-related concerns.
MAJOR
1. I would like to see a better justification for these analyses given current discussions on the role of genetic ancestry and environment in disparities. The introduction sets it up with thinking generally between genetic and environmental differences, but the paper is focused on *genetic ancestry*. Clarification is needed for how these are connected and why this matters. (lines 70-75) I see the added line at the end, but this is not tied to a larger question as to why this study matters in the field.
2. Additionally, why is MCPS is a good study for this? The introduction should have a background of ancestries and how that interacts with the environmental differences. This would require the authors to draw upon the epidemiology of the traits in Mexico City and how that intersects with social stratification and genetic ancestry. The results are compelling, but the current

iteration lacks the "why" in the introduction and the "so what" in the discussion. This should also be included in the abstract, where right now it jumps from GxE to ancestry without a connection.
2.1. Lines 303-305, about how these associations could lead to misleading inference, is absolutely vital and should be hammered home much more often in the introduction and the results section. I know different folks have different feelings about this, but I think a bit of interpretation in the results section would be very helpful to ensure this paper is not misunderstood with a general audience.
3. Throughout the piece, I would soften the language to be less absolute. For example, consider rephrasing "estimating unconfounded" in line 36 with something not quite as strong, such as "addressing confounding" or "while controlling for between-family confounding".
3.1. In line 77, you could change to "therefore associations between within-family genotype variation and phenotype are (*assumed*) to be due only to the causal effects of inherited alleles." This is consistent with the note at the end of the paragraph, in which differences can be due to mediation.
3.2. Line 343: caution when using the terminology of causality. There are specific criteria to fulfill according to many causal inference frameworks, and it is not clear that these are fulfilled in this study design. There have been other studies demonstrating that there is confounding in aggregation between ancestry proportions and outcomes that is eliminated with granular labels, but this study is important as it looks at family structures. (You also claim to be the first to do this, but later (line 369 and on), there is a description of a similar study in Greenland families. (I do not think you need to be the first to be important, so would remove that language entirely.))
4. In your discussion, how do these results compare to previous results? What is new? Association with height is already known, as well as differences in metabolic traits. What is *new*, such as the comparison between these effect estimates and prior literature, should be highlighted.
5. The language when referring to ancestries does not follow current guidelines regarding the distinction of inferred ancestry based on reference populations versus some "truth", which cannot be ascertained. For example, line 97 could be altered to say "Genome-wide proportions of *inferred* IAM..." followed by stating the reference populations. This makes it clear which reference groups were used (TGP and HGDP) and that these are relative to those groups.
5.1. Justify the use of 100% EUR baseline in the main results. Why is EUR the standard? There are different reasons others use, such as it is the most common ancestry (not relevant for this population) or they care comparing results to previous findings, which were primarily done in EUR groups. I am completely fine with its use and how it was defended in the response to reviewers! But a line or two as to why it was chosen, not as a default for humans but for this specific analysis, would go far.
6. What was the correlation between a matched set of alleles for the traits? It is expected that due to genetic drift, a large proportion of GWAS SNPs will be positively correlated due to chance and not specifically from the trait. An appropriate comparison would be a matched trait-agnostic set of similar frequencies. Also, functional alleles are more likely to be common and therefore shared across ancestries. Again, the comparison for attenuation should be done with a different set of alleles that are matched by frequency to make a stronger case for these specific alleles being important. You could even use the SNPs matched in the later section for this analysis, so hopefully it isn't a big lift.

MINOR
7. Change lnOR to odds ratio to aid in interpretation. (Is the log odds really 5.13? That seems huge! If this is actually the OR, that would be more consistent compared to the unrelateds analysis for T2D.)
8. Include estimates of precision for correlations in results (line 112).
9. How was the increase of T2D prevalence estimated at 19%? This may have been detailed somewhere in the supplement (if so, I apologize), but it would be helpful to make it clearer in the results in terms of how you got from OR to prevalence differences.
10. The limitations for sample sizes could be partially addressed by redoing one of the analyses with a smaller sample of unrelated individuals. Additionally, if you estimated power, just say the actual numbers in the text (don't make me track down supplementary note 2 in order to understand how big of a limitation this is). It seems low based on the figure, but how low is it?
11. [This is not important for this manuscript per se but would be interesting to do in future work.] Do individuals identify themselves in terms of "ethnicity" or "group" within Mexico? How does these results within those self-identified groups? And do the results differ by district? I'm not as familiar with the geography and history of Mexico City, but considering the population substructure present in these participants, I wonder if the results would differ by district or by how people identify themselves (if that information is available).

FIGURES
12. Figure 2 may be more impactful as a heat plot showing direction of effects. Also, I would suggest changing the title to be specific to unrelated individuals and the legend to include the limitations. However, depending on the authors and editorial team, if there is a concern that this data could be used to further race science, I would even suggest removing this figure entirely as Figure 3 gets the point across more effectively with less chance of misinterpretation.
13. Is there a way to make Figure 4 more distinct from Figure 3? Perhaps to show how many of them have null associations? This could include making null associations in dashed lines and the non-null ones in bold? Just to make it more visually striking and get the message across as opposed to a data dump.

(Remarks on code availability)

Referee #2

(Remarks to the Author)
The authors responded to my previous critiques, and I have no further major concerns.

Given the topic of this paper & the relative novelty of its approach, I anticipate that it might attract considerable attention within and beyond the academy. In the interest of communicating the paper's objectives and results as clearly as possible, I want to flag the following sentences for review by the author/editor, as they might imply, especially to a general reader, the existence of an overly simple nature vs. nurture binary.

"but the extent to which differences are caused by genetic or environmental factors is unknown for most complex traits" --> could be simplified to just "but the extent to which differences are caused by genetic factors is unknown for most complex traits"

"estimating unconfounded effects of genetic ancestry on phenotypes" --> "estimating effects of genetic ancestry on phenotypes that are unconfounded by environmental differences between families"

"the relative contribution of genetic and environmental factors, and their interactions, to these differences. In this study, we combine large scale data and a gold standard approach to address this question." --> You're not really quantifying the contribution of environmental factors or genexenvironment interactions here. could simplify, again, to just "the relative contribution of genetic factors to these differences".

"However, these associations do not distinguish between within-family ancestry effects and environmental effects that are correlated with genetic ancestry proportions" --> suggest extending this sentence to say "environmental effects that are correlated with genetic ancestry proportions between families"

(Remarks on code availability)

Referee #3

(Remarks to the Author)
The manuscript has clearly improved in response to the reviewers' feedback, and the tone is now more cautious and responsible. I appreciate the additional care taken to frame the results in a way that supports careful interpretation by the community.

(Remarks on code availability)
The provided README files and simulation data ease usage of the code and methods for the community

Version 2:

Reviewer comments:

Referee #1

(Remarks to the Author)
I thank the authors for their responsiveness to feedback and this improved version of the manuscript. The clarifications throughout the manuscript greatly helped with contextualization and interpretation, as well as the additional methodological details.

While I disagree with the authors use of causality, even drawing from Mendelian randomisation in which certain assumptions need to be met (which I do not think this study design can do). My understanding of mechanistic causality is in contrast to that of unconfounded association, under which I would place this study design. The associations here are examining how ancestry may correlate with certain complex diseases, not that the AIMs are directly related to the biology of that trait. However, I think this is one of those philosophy of epidemiology "agree to disagree" moments and do not think it requires any additional revisions before publication.

A very very very minor point, mainly to set the tone for the piece, is to remain the word "species" at the beginning of the manuscript. Perhaps "Within humans, multiple...". And then you can remove the "In humans" part of the third sentence. While there isn't anything wrong with the original, I think it will better center the people in this study, especially since it is likely to generate some discussions. And then adding a "can" on line 65 ("Human populations *can* differ in phenotype means..."). I know that I sound like a broken record, but these things can help pre-empt some responses so that the rest of the manuscript is received in the appropriate context.

(Remarks on code availability)

Open Access This Peer Review File is licensed under a Creative Commons Attribution 4.0 International License, which permits use, sharing, adaptation, distribution and reproduction in any medium or format, as long as you give appropriate credit to the original author(s) and the source, provide a link to the Creative Commons license, and indicate if changes were made.
In cases where reviewers are anonymous, credit should be given to 'Anonymous Referee' and the source.
The images or other third party material in this Peer Review File are included in the article's Creative Commons license, unless indicated otherwise in a credit line to the material. If material is not included in the article's Creative Commons license and your intended use is not permitted by statutory regulation or exceeds the permitted use, you will need to obtain permission directly from the copyright holder.
To view a copy of this license, visit https://creativecommons.org/licenses/by/4.0/

We thank all three expert reviewers for their comments, queries and suggestions, which have all helped improve our manuscript. In our revision, we have tried to refine the language, be more specific about the interpretation of results and have added results from a number of new theoretical and empirical analyses to strengthen the conclusions. We respond to each of the individual reviewer comments below.

Referee #1 (genetic epidemiology, underrepresented populations):

The authors present their work comparing unrelated individuals and nuclear families to disentangle the relationship between estimated genetic ancestry proportions and a selection of complex traits. Their results are compelling, with some supported by previous work and others being novel. I would like to state for the record that I am not against the topic entirely, only that it must be handled with care to avoid overinterpretation and misappropriation. Having a reference like this would be important for future work, both to dispel incorrect beliefs around race essentialism and eugenics and lay a groundwork for more within-family analyses. But it must be well-justified, presented within a specific context, and measured in its conclusions. My concerns are mostly with the framing of the work, including a lack of justification for the research question or selection of European ancestries as being the default when it is not the most common in this population, as well as the strong causal language when it is not justified. I expand on these concerns below with suggestions for the authors.

We thank the referee for the time and effort spent reviewing our paper, for the insightful and critical comments, and for specific suggestions on how to address them.

- The overall study question is not well justified. What do we have to gain from these results? Why is this question important? How does it influence the public health or precision medicine in these communities? More information on the background setting up this question is required. (This is a side note, but I would suggest not mentioning agriculture and dog breeds at the beginning of a paper intending to talk about admixed people.)

We thank the reviewer for this comment. The general research question is whether genetic ancestry causes mean trait differences, either through trait-relevant biology ("trait-specific polygenic load") or social/environmental mediators, or if any such trait differences are due to known or unknown socio-environmental factors that are confounded with ancestry. As articulated by Reviewer #2, "Why do populations differ in their trait averages?". We are interested in understanding both the why and how. The specific research question in our study is to quantify and understand the effects of ancestry as an exposure on outcomes in Mexico City. Understanding the nature of ancestry effects is relevant to population health in Mexico because it can help improve risk classification and inform disease prevention.

If there are between-population differences in disease and other complex traits that are caused by drift or natural selection, then quantifying them goes towards explaining observed phenotypic variation in human traits (rather than just focusing on allele frequencies that differ between populations). It may also lead to useful findings. For example, mean plasma levels of the biomarker lipoprotein(a) differ between populations of African and European ancestry, and this is mostly due to genetic factors. To make the aim clearer, we added text in the Introduction (page 4, line 88-91).

We have also deleted the description of agriculture and dog breeds from the first sentence of the introduction.

- There is a lack of acknowledgement throughout the manuscript about their structured population. Assortative mating in highly admixed populations in which classism and colorism are tightly linked is not unexpected. And yet the authors treat many analyses with a single pooled analysis without considering the geographic and socioeconomic structuring of the study population and recruitment area. Sensitivity analyses could address some of these concerns, but the larger point is that there is little information about the health of these patients within their communities.

We thank the reviewer for this comment. We agree that the population is structured, both genetically and socio-environmentally, and indeed we leverage the genetic structure in a family analysis to address our scientific questions. Individuals in the Mexican City Prospective Study were recruited from two districts of Mexico City (Coyoacán and Iztapalapa) that differ in socio-economic environment. Participants were not recruited because they are known to be patients – the experimental design was a prospective study. We have now provided a detailed breakdown of the baseline characteristics of the two districts (updated Supplementary Table 2). We also provide new analyses in which we fit educational attainment (EA) as a proxy for a socio-economic indicator in the within-family analyses. Please see below our detailed response and results from these analyses to address the questions about trait-specificity of polygenic load and the question of mechanism ("proximal trait biology" versus "distal trait mediation").

- Overall, it would be beneficial to soften the language. These statistical tests cannot be used to establish causality, for while within-family analyses may control for differences in shared environment, those are not the only environmental differences that can matter. For example, what is described as a result of direct effects (within family analyses) could also be due to colorism, which is an environmental or social determinant of health, and can influence siblings differently within a family. The discussion states that environmental effects can only occur between families (lines 269-271), which is not true, especially in highly admixed populations in which a single family can have a wide range of skin tones. While a family may provide the opportunity for education consistently within their family, the effects of colorism (such as stress) are more likely to be felt for T2D. In the discussion, causality is mentioned and justified but there is no test of causality within the manuscript and you cannot conclude causality, just association and the suggestion of potential direct effects.

We thank the reviewer for this helpful comment. We have now substantially changed the language (including in the title) and have tried to clarify in more detail what the possible explanations are for a within-family association with ancestry. We have provided a new Supplementary Figure 1 that shows the possible causal paths, which demonstrates that without additional information we cannot distinguish between a direct/causal biological effect and a causal effect that is mediated by socio-environmental factors. An example of the latter would be mediation through skin tone, as suggested by the reviewer. We have provided additional analyses with counts of trait-increasing alleles (cTIA) associated with height, T2D, and EA. The results from these analyses are consistent with the hypothesis that the observed within-family effects associated with height and T2D are likely caused by a genome-wide accumulation of trait-increasing (or trait-decreasing) alleles acting upon these traits.

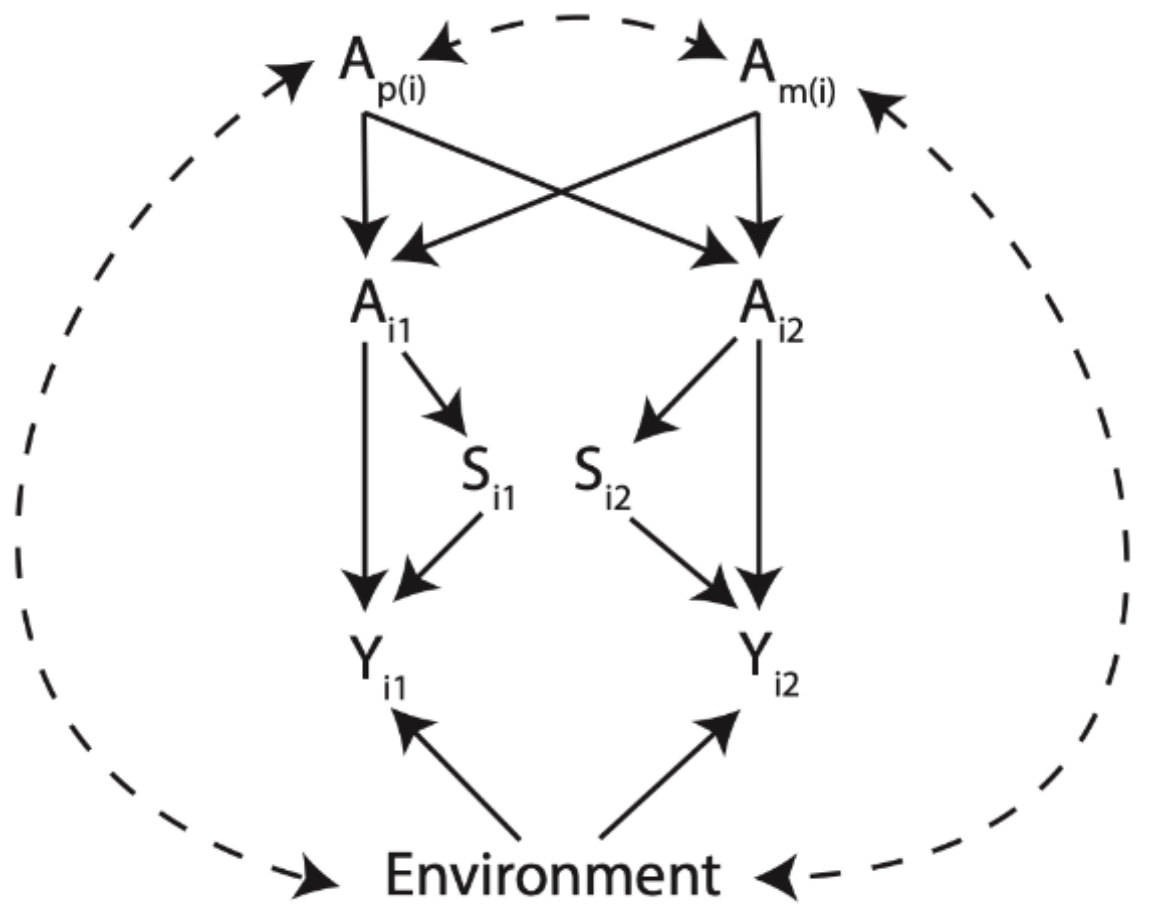

Supplementary Figure 1: Causal paths that lead to an association between ancestry (A) and a trait (Y) at the population and family level. The black single-headed arrows represent causal effects, and the dashed double-headed arrows are correlations (not necessarily causal). $A_{p(i)}$ and $A_{m(i)}$ are the ancestries of the father and mother in family i. A_{i1} and A_{i2} are the ancestries of two siblings in family i. This shows that the sibling ancestries are conditionally independent of the environment given the parental ancestries (implying that their difference is also independent). This also shows that the within-family ancestry effect of A_{ij} on Y_{ij} can 'directly' affect the trait through a mechanism such as proximal trait biology (A → Y) or be mediated through S_{ij}, the ancestry-induced social environment of sibling j in family i (A → S → Y), for example the effect of ancestry on skin tone, which induces social discrimination that affects the trait.

- The use of 100% EUR as the reference group goes against the recommendations of two NASEM reports on population descriptors in genetics and genomics (2023) and race/ethnicity in biomedical research (2024). I acknowledge that these are two US-based set of recommendations, but they may still be helpful for this manuscript in order to be consistent with newer guidelines and to minimize the potential for misuse of these results on a sensitive topic.

We thank the reviewer for this comment and for pointing out that these are context-specific recommendations. We had the choice to leave out either IAM or EUR because we can only fit 3 effects and have 4 ancestries, and IAM & EUR together make up most (>90% on average) of the estimated genome-wide ancestry. We chose to leave out EUR for several reasons. Firstly, we believe it would look strange to conduct an ancestry association analysis in the Mexican population and then report effects of EUR, AFR, and EAS ancestries (relative to IAM) across the entire study, i.e. leaving out the indigenous ancestry that comprises the majority ancestry proportion in MCPS. Secondly, if we were to display the results relative to an IAM baseline, then it will look as if EUR ancestry individuals are taller and healthier, and this may also lead to misuse/misinterpretation. Statistically it does not matter, and the best way to present these results will always be a somewhat arbitrary choice.

For completeness, we here show the results for when we express results relative to an IAM baseline. As can be seen, the main difference is that essentially the signs of effects are flipped: previously the main contrast was IAM-EUR and the results below are for EUR-IAM.

Reviewer Table 1: Main results when performing the analysis fitting EUR, AFR and EAS ancestry proportions in the model. The first two rows for each trait or analysis model are the results that were shown in the original submission.

Trait	Source	IAM			AFR			EAS		
		**Estimate**	**SE**	***P***	**Estimate**	**SE**	***P***	**Estimate**	**SE**	***P***
**Height**	within-family effect	-1.51	0.26	1.02E-08	0.59	0.84	0.48	-1.45	0.89	0.10
	between-family effect	-0.05	0.27	0.85	0.19	0.91	0.83	1.21	1.00	0.22
		**EUR**			**AFR**			**EAS**		
		**Estimate**	**SE**	***P***	**Estimate**	**SE**	***P***	**Estimate**	**SE**	***P***
	within-family effect	1.51	0.26	1.02E-08	2.10	0.79	8.05E-03	0.06	0.83	0.94
	between-family effect	0.05	0.27	0.85	0.24	0.85	0.78	1.26	0.95	0.18
**T2D**	***REML**** ***(mixed model on 0-1 scale)***	**IAM**			**AFR**			**EAS**		
		**Estimate**	**SE**	***P***	**Estimate**	**SE**	***P***	**Estimate**	**SE**	***P***
	within-family effect	0.41	0.12	4.67E-04	-0.30	0.37	0.42	0.48	0.39	0.22
	between-family effect	-0.23	0.12	0.06	0.50	0.39	0.20	-0.40	0.42	0.35
		**EUR**			**AFR**			**EAS**		
		**Estimate**	**SE**	***P***	**Estimate**	**SE**	***P***	**Estimate**	**SE**	***P***
	within-family effect	-0.41	0.12	4.67E-04	-0.71	0.35	0.04	0.07	0.37	0.85
	between-family effect	0.23	0.12	0.06	0.73	0.37	0.05	-0.17	0.40	0.67
	Mixed model logistic regression*	**IAM**			**AFR**			**EAS**		
		**Estimate**	**SE**	***P***	**Estimate**	**SE**	***P***	**Estimate**	**SE**	***P***
	within-family effect	5.13	1.35	1.51E-04	-3.72	4.31	0.39	6.05	4.17	0.15
	between-family effect	-2.98	1.36	0.03	6.62	4.41	0.13	-5.02	4.53	0.27
		**EUR**			**AFR**			**EAS**		
		**Estimate**	**SE**	***P***	**Estimate**	**SE**	***P***	**Estimate**	**SE**	***P***
	within-family effect	-5.13	1.38	1.91E-04	-8.84	3.95	0.02	0.93	3.59	0.80
	between-family effect	2.98	1.39	0.03	9.59	4.13	0.02	-2.05	3.88	0.60

****Linear mixed model includes both family and IBD random effects in the GCTA REML analysis**<br>*****Using a generalised linear mixed model with a single random effect of family fitted***

- If I were a general audience reader of this journal, I would not understand what you mean by direct genetic ancestry effects. Is it having over a certain proportion of IAM ancestry would therefore predispose you? Or is it just that you are more likely to have specific ancestry-specific haplotypes or variants? This distinction is incredibly important for the interpretation of results. Taking T2D as an example, there is nothing inherently "bad" about having IAM ancestry, just that having more IAM ancestry makes it more likely to have more variants that may increase risk. I realize this may seem like a minor distinction, but can have an enormous effect on the reception and utilization of these results.

We thank the reviewer for this comment. We have revised the text and replaced "direct genetic ancestry effect" with "within-family effect of ancestry" throughout the manuscript, supplementary information, and figures/tables. The new title is "Within-family effect of ancestry on complex traits in a Mexican population". We have also tried to clarify the interpretation of our detected within-family effects. In particular, we now explain (page 4, lines 79-84) that the within-family effect of ancestry proportion on outcomes treats the overall genome-wide ancestry proportion as the "exposure". As shown above in the Supplementary Figure 1, IAM ancestry can have an effect on T2D because, everything else being the same, a larger proportion of IAM ancestry in the genome carries more T2D risk alleles (IAM ancestry → T2D risk alleles → T2D), or because a larger proportion of IAM ancestry in the genome leads to social environments that predispose to T2D (e.g., IAM ancestry → skin tone → social environment → T2D).

For further context regarding the ancestry "exposures", the ADMIXTURE software used in our analysis estimates per-individual ancestry proportions (Q parameters) and ancestry-specific allele frequencies (F parameters) by maximizing the log-likelihood of observed genotypes under a model of K postulated ancestral populations (Alexander et al. 2009, Genome Res.; Alexander et al. 2011, BMC Bioinformatics). This is performed on a set of LD-pruned variants treated as independent. Therefore, haplotype information is not directly used in estimating the ancestry "exposures" used in our study. However, these ancestry proportions are estimated from genetic variants that may be differentially segregating between populations and among differentiated haplotypes. Moreover, as stated above, the effects of these ancestry proportions could involve direct effects of such genetic variation, or be mediated through social factors.

Specific analyses:

- The authors state in lines 204-207 that "if the polygenic score captures the direct ancestry effect", then you would see attenuation. However, it is difficult to conceptualize or make this direct point when (1) PGS can differ in distribution by ancestry because of trait differences AND (2) they can differ because of random drift unrelated to the trait but more a product of distance from the training dataset. It is therefore difficult to say with the language currently included in the manuscript that the attenuation of effects is because of anything trait-specific, or because the training PGS was from a predominantly EUR background group. This is consistent with a larger concern above regarding the softening of language, or allowing more nuance.

We thank the reviewer for this interesting comment. We have performed additional analyses to address the question of "trait specificity". Specifically, we have fitted a cTIA for T2D (or EA) in the analysis of height. The results below and in the updated Supplementary Table 8 show that for both traits, there is no attenuation when we fit the "wrong" cTIA in the model. We also fitted models in which EA is fitted in the model as a covariate as a proxy for a socioeconomic indicator. If the observed within-family effect is mediated by EA-associated socioenvironmental factors, then we should observe an attenuation of the within-family effect size.

The new models we fitted were:

- ***Height ~ between-family effects + within-family effects + cTIA$_{T2D}$***
- ***Height ~ between-family effects + within-family effects + cTIA$_{EA}$***
- ***Height ~ between-family effects + within-family effects + EA***
- ***T2D ~ between-family effects + within-family effects + cTIA$_{height}$***
- ***T2D ~ between-family effects + within-family effects + cTIA$_{EA}$***
- ***T2D ~ between-family effects + within-family effects + EA***

The full results are shown in the updated Supplementary Table 8. Here we show the results on the IAM ancestry.

Trait	Model	Source	Estimate	SE	*P*
Height	Height ~ ancestries (between and within)	IAM within-family effect	**-1.51**	0.26	1.02E-08
	+ cTIA$_{height}$	IAM within-family effect	**-0.43**	0.25	0.08
	+ cTIA$_{T2D}$	IAM within-family effect	-1.47	0.26	2.70E-08
	+ cTIA$_{EA}$	IAM within-family effect	-1.49	0.26	1.88E-08
	+ EA	IAM within-family effect	-1.52	0.26	8.49E-09
T2D	T2D ~ ancestries (between and within)	IAM within-family effect	**0.41**	0.12	4.67E-04
	+ cTIA$_{T2D}$	IAM within-family effect	**0.24**	0.12	0.04
	+ cTIA$_{height}$	IAM within-family effect	0.41	0.12	4.61E-04
	+ cTIA$_{EA}$	IAM within-family effect	0.41	0.12	5.86E-04
	+ EA	IAM within-family effect	0.41	0.12	4.43E-04

Results from these additional analyses are consistent with the hypothesis that we are detecting a biological trait-specific effect.

- How do you differentiate between selection and assortative mating, which has already been shown with ancestry proportions? It has already been shown to occur with height in other populations, so why not with this population? And if it is assortative mating, how does that interpretation differ from "natural selection", particularly in the interpretation of genetic determinism and racial essentialism?

We thank the reviewer for the question about assortative mating and should have explained our test of selection better. We do not think that assortative mating has influenced our inference on selection versus drift. Our analyses of drift versus natural selection contrasts the frequencies of trait-associated alleles in a ~100% IAM population vs a 100% external EUR population. Allele frequencies in our inferred ~100% IAM population were validated with an independent IAM sample from the HGDP panel (Supplementary Figure 6). Therefore, we have not used the MCPS individuals who are admixed to draw inference about selection at trait-associated loci in the ancestral populations. In the absence of natural selection but with both drift and assortative mating on height in ancestral IAM and EUR populations, we would not expect the observed systematic difference in average height-increasing alleles frequency between these populations.

For these reasons, assortative mating on ancestry in the current admixed population does not affect the data and method we have used to infer natural selection. We are sorry that we did not make that clearer and have now added text to make this explicit (page 36-37, line 741-743).

We believe that a study of what drives the observed correlation in ancestry proportion between parents when ancestry proportions are correlated with traits (e.g., height) is very interesting but outside the scope of our current study.

Language choices:

In addition to agriculture and dog breeds, I would avoid calling an admixed population a "natural experiment" (line 253). I know it is used often and honestly is not on its own problematic, but in this context it could come across as offensive to this and other admixed populations.

We thank the reviewer for this helpful suggestion and have now deleted that sentence.

Referee #2 (behavioural genetics):

Review of "Direct effect of genetic ancestry on complex traits in a Mexican population" by Wang et al. (2025), submitted to Nature

This paper estimates within- and between-phenotype associations between genetic ancestry and 15 complex phenotypes in a sample of admixed individuals from Mexico City. It reports evidence that Indigenous American (IAM) ancestry is associated, when comparing among unrelated individuals, with shorter stature, higher risk of Type 2 diabetes (T2D), and lower educational attainment (EA). However, when comparing within-families, IAM ancestry is not associated with EA, whereas associations with height and T2D persist. The within-family ancestry-phenotype association is interpreted as a causal effect of ancestry because of the randomness of within-family genetic segregation. The finding of a "direct" effect of ancestry on T2D and height is buttressed with results from analyses polygenic burden and selection. The major strengths of the study are (1) its focus on individuals with complex genetic ancestries, particularly on those with IAM ancestry who live in the global South, who are extremely underrepresented in genetics research generally, (2) its rigorous approach to estimating within-family associations, and (3) its integration of methods across medical, population, and evolutionary genetics. It addresses a question of long-standing, contentious interest: Why do populations differ in their trait averages? I anticipate that the paper will draw the attention of a broad readership.

My major criticism of the paper, which I think is readily ameliorable, is its characterization of the "direct" effect of ancestry. I agree with the authors that, because segregating genetic variance is randomly "assigned" at conception, the within-family association between genetic differences and phenotype differences can be interpreted as an estimate of the causal effect of the genetic differences tagged. As the authors describe, this causal effect is not confounded by dynastic environmental differences that are correlated with ancestry across generations and between-families, i.e., what behavioral geneticists would call "passive gene-environment correlation" (Plomin, deFries, & Loehlin, 1977).

We thank the reviewer for these comments and for agreeing that within-family ancestry effects are not confounded with passive GE.

This causal effect, however, cannot be equated with a purely "genetic" effect, as it still might depend on evocative or active gene-environment correlations. Sandy Jencks (1972) made this point conceptually with his "red-headed children" thought experiment. More recently, Dalton Conley and his colleagues made this point in their 2019 analysis of skin tone and hypertension ("New Evidence of Skin Color Bias and Health Outcomes Using Sibling Difference Models: A Research Note"). Their design was conceptually similar to the present manuscript, in that they analyzed sibling differences to estimate causal genetic effects on a complex medical trait. Using a sample of admixed black/Latinx Americans, they found that within-sibling differences in skin tone were associated with elevated rates of hypertension. However, they interpreted this association as evidence for colorism (social discrimination against darker skin tones). The hypothesized pathway, in other words, was gene --> proximal phenotype (skin tone) --> social environment (discrimination) --> distal phenotype (hypertension).

We thank the reviewer for pointing to this interesting paper which we read and now cite. We elaborate further below how we have addressed the questions of terminology and causation.

Regarding the 2019 paper from Conley and colleagues, we note that the evidence for within-family associations between skin tone and hypertension are quite weak (p-values <0.1 and <0.05, without any correction for multiple testing; there are many models in which multiple fixed effects are estimated), that the association between PC1 ("African ancestry") and skin tone is not statistically significant (Table A8, model 1), that none of the six chosen SNPs are associated with skin tone (Table A8, model 2) and that the authors themselves are quite cautious in their overall conclusions. The authors state "Genes that predict both complexion and hypertension risk may vary between full siblings and dizygotic twins (i.e., genetic pleiotropy). In this case, our estimates would potentially be biased by not accounting for a possible biologically endogenous genetic link between complexion and hypertension". [We note that pleiotropy does not need to be invoked because ancestries can differ in polygenic loads for complexion and hypertension without the same genes being involved, an example of global ancestry confounding or admixture induced population stratification]. Further, "Although there may be other pleiotropic alleles that we have not tested, and although the linkage patterns between the SNPs and cardiovascular-related causal alleles may differ in the black and non-Hispanic white populations, we nonetheless believe the weight of evidence suggests that the effects shown in the main analysis are more likely the result of social pathways than within-family variation in genetic background."

The authors acknowledge that genetic effects might have social mechanisms in the Discussion (p. 11), where they write: "While our within-family direct ancestry effect estimates can be given a causal interpretation, they do not imply a specific mechanism. It is possible that ancestry differences cause phenotype differences through social mechanisms such as ancestry-related appearance-based discrimination. However, our estimates are consistent with ancestry associated phenotype differences in height and T2D risk being entirely explained by direct ancestry effects (estimated within-family): if appearance related discrimination exerted an environmental effect on these phenotypes, it is likely this would also operate between families and induce a between-family ancestry association in our design, which we do not observe for height and T2D."

I am not entirely convinced by this argument that a social mechanism connecting ancestry with health would necessarily operate as strongly between-families as within-families. Indeed, several scholars have argued that colorism operates as an intra-racial and intra-family social process (see, e.g., Lori Tharps, "Same Family, Different Colors: Confronting Colorism in America's Diverse Families"; Maria Abascal and Denia Garcia, "Pathways to Skin Color Stratification: The Role of Inherited (Dis)Advantage and Skin Color Discrimination in Labor Markets"; Jessica Kizer, "Skin Color Stratification and the Family: Sibling Differences and the Consequences for Inequality.") One could argue that these previous scholars have prematurely attributed within-family associations between skin tone and complex traits to social processes rather than to "inside the skin", biologically-mediated effects of genetic ancestry. My point, though, is that we don't really know, and this study doesn't offer strong evidence to resolve the question. There is not (yet) dispositive evidence about the specific mechanisms that connect genetic ancestry with complex phenotypes.

We thank the reviewer for these references and for articulating the possible explanations of our observed effects. We agree with the point that the reviewer makes: if ancestry-associated social mediation mechanisms at the individual level fully explain the population-level effects then we would not expect a between-family effect in the analysis of family data when jointly fitting within and between effects. We have removed the sentence about the

between-family effect accordingly and have elaborated the discussion (page 12-13, lines 284-295).

We thank the reviewer for citing the three publications, which we have now read. We note that the Tharps book is anecdotal and that, to our knowledge, the Kizer thesis has not (yet) resulted in a peer-reviewed paper and that the results in the thesis point to a number of significant between-family effects of skin tone (adult male arrest, Table 2.4; household income, Table 4.3). We also note that the results from the sibling fixed-effects model (of labour market outcomes on skin tone) were statistically not significant in Abascal and Garcia (their Table 2). We have chosen not to cite these papers in the context of the possible pathways of their specific examples and analyses because of the reasons articulated by the reviewer (the possibility of a biologically-mediated effect) and the risk of misinterpretation.

Given this lack of evidence about mechanism(s), and the potential misinterpretations of work connecting genetic ancestry to health outcomes, I think that the parameter the authors are estimating—-the causal effect of genetic ancestry on complex traits—-should be described with more care and caveat throughout the whole manuscript, not just in the Discussion. For example, in the Introduction, after the authors introduce the term "direct effects of ancestry", they should note that, as with direct genetic effects operating within-ancestries, "direct" effects can be mediated by culturally-specific gene-environment correlations. Similar caveating should continue through the Results and Discussion. I, personally, would avoid the term "direct" and just use "causal," being sure to emphasize that genetic causes can operate through social mechanisms ("transactional genetic effects" was the term used by Laurel Raffington and colleagues in "Polygenic Scores in Developmental Psychology").

We thank the reviewer for the summary of our work, for suggesting how we can improve terminology and provide more clarity on the interpretation of estimated effect sizes and for providing helpful references. Some of these comments echo those from Reviewer #1 and we have made substantial changes to the text, including changing the title, adding text to the Introduction and expanding the Discussion. We have also provided a causal path diagram with explanation (Supplementary Figure 1), that shows that a causal effect can either be direct (e.g., increased count of trait-increasing alleles (cTIA) for height or liability to T2D) or mediated through socio-environmental factors acting upon a proximal trait such as skin tone.

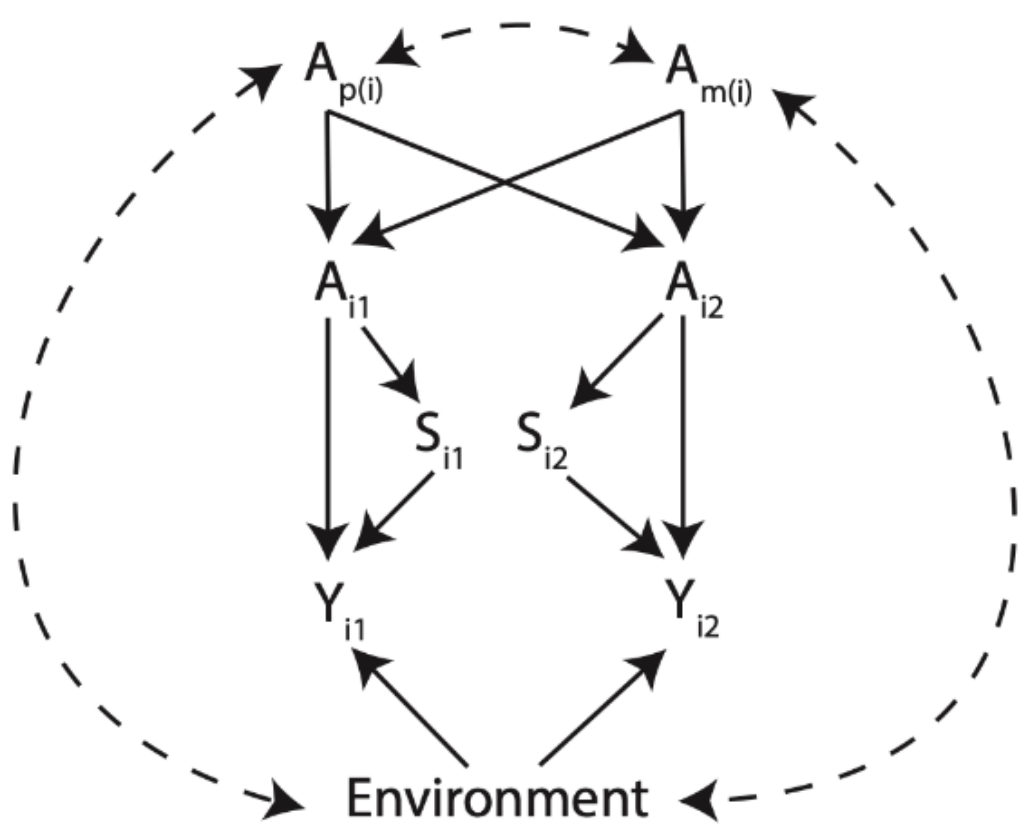

Supplementary Figure 1: Causal paths that lead to an association between ancestry (A) and a trait (Y) at the population and family level. The black single-headed arrows represent

causal effects, and the dashed double-headed arrows are correlations (not necessarily causal). $A_{p(i)}$ and $A_{m(i)}$ are the ancestries of the father and mother in family i. A_{i1} and A_{i2} are the ancestries of two siblings in family i. This shows that the sibling ancestries are conditionally independent of the environment given the parental ancestries (implying that their difference is also independent). This also shows that the within-family ancestry effect of A_{ij} on Y_{ij} can 'directly' affect the trait through a mechanism such as proximal trait biology (A → Y) or be mediated through S_{ij}, the ancestry-induced social environment of sibling j in family i (A → S → Y), for example the effect of ancestry on skin tone, which induces social discrimination that affects the trait.

We have performed additional analyses to address the question of "trait specificity" and to quantify the effect of fitting educational attainment (EA), as a proxy for socioeconomic circumstances, in the model. In particular, we have fitted a cTIA for T2D in the analysis of height and fitting a cTIA for height in the analysis of T2D. The results show that for both traits, there is no attenuation when we fit the "wrong" cTIA in the model.

The new models we fitted were:

- ***Height ~ between-family effects + within-family effects + $cTIA_{T2D}$***
- ***Height ~ between-family effects + within-family effects + $cTIA_{EA}$***
- ***Height ~ between-family effects + within-family effects + EA***
- ***T2D ~ between-family effects + within-family effects + $cTIA_{height}$***
- ***T2D ~ between-family effects + within-family effects + $cTIA_{EA}$***
- ***T2D ~ between-family effects + within-family effects + EA***

The full results are shown in the updated Supplementary Table 8. Here we show the results on the IAM ancestry.

Trait	Model	Source	Estimate	SE	*P*
Height	Height ~ ancestries (between and within)	IAM within-family effect	**-1.51**	0.26	1.02E-08
	+ $cTIA_{height}$	IAM within-family effect	**-0.43**	0.25	0.08
	+ $cTIA_{T2D}$	IAM within-family effect	-1.47	0.26	2.70E-08
	+ $cTIA_{EA}$	IAM within-family effect	-1.49	0.26	1.88E-08
	+ EA	IAM within-family effect	-1.52	0.26	8.49E-09
T2D	T2D ~ ancestries (between and within)	IAM within-family effect	**0.41**	0.12	4.67E-04

	+ $cTIA_{T2D}$	IAM within-family effect	**0.24**	0.12	0.04
	+ $cTIA_{height}$	IAM within-family effect	0.41	0.12	4.61E-04
	+ $cTIA_{EA}$	IAM within-family effect	0.41	0.12	5.86E-04
	+ EA	IAM within-family effect	0.41	0.12	4.43E-04

Results from these analyses support our claim that we are detecting a biological trait-specific effect.

My other major question about the paper concerns power. Given the high parental correlations for ancestry, the relatively small within-family variance in ancestry, and some reasonable priors about effect sizes, what is the sample size that would be necessary to reliably identify within-family effects of ancestry on these 15 traits, including on EA where the authors report a null association? How far below (or not below) that mark is the current sample? I anticipate that this report might open to door to many subsequent papers aiming to use admixed samples to investigate genetic contributions to between-group differences in complex traits, and giving the field more concrete guidance regarding power and sample size would be useful.

We thank the reviewer for the question about statistical power. Since we have already performed the empirical analyses, what we can conclude from our own study is contained in the estimates of the effect sizes and their standard errors and the selection process (focusing on statistically significant results when performing analyses on multiple traits). As detailed in the Methods (page 34, lines 675-676), we adjusted the p-value threshold for the number of independent tests we conducted. We have now added a sentence in the discussion that the estimates that are statistically significant in the within-family analyses are affected by the winner's curse (page 14, lines 335-337).

For educational attainment, the IAM estimate of the within-family effect size was -0.12 with a standard error (SE) of 0.26. If we use the actual SE of 0.26 and the type-I error rate used in the study (5.89e-4), we can quantify the power of detection given a true within-family effect size on that scale (either positive or negative). We performed this power calculation (using a non-centrality parameter of b^2/0.26^2) and find that the power to detect a true effect size of (+/-) 0.5, 0.75, 1.0 and 1.25 is 0.07, 0.29, 0.66 and 0.91, respectively. Therefore, we could not reject a true effect size in the range of -0.75 to +0.75 but have good power to reject a true within-family effect size smaller than -1 or larger than +1. For comparison, the effect size at the population level was -2.03.

We agree with the reviewer that giving some guidance with respect to statistical power to detect a within-family effect size associated with ancestry is useful. We therefore added a derivation of such power in Supplementary Note 2 and added a figure with a summary of the power of detection for a range of parameters and provided computer code so that experimenters can input their own study-specific parameters.

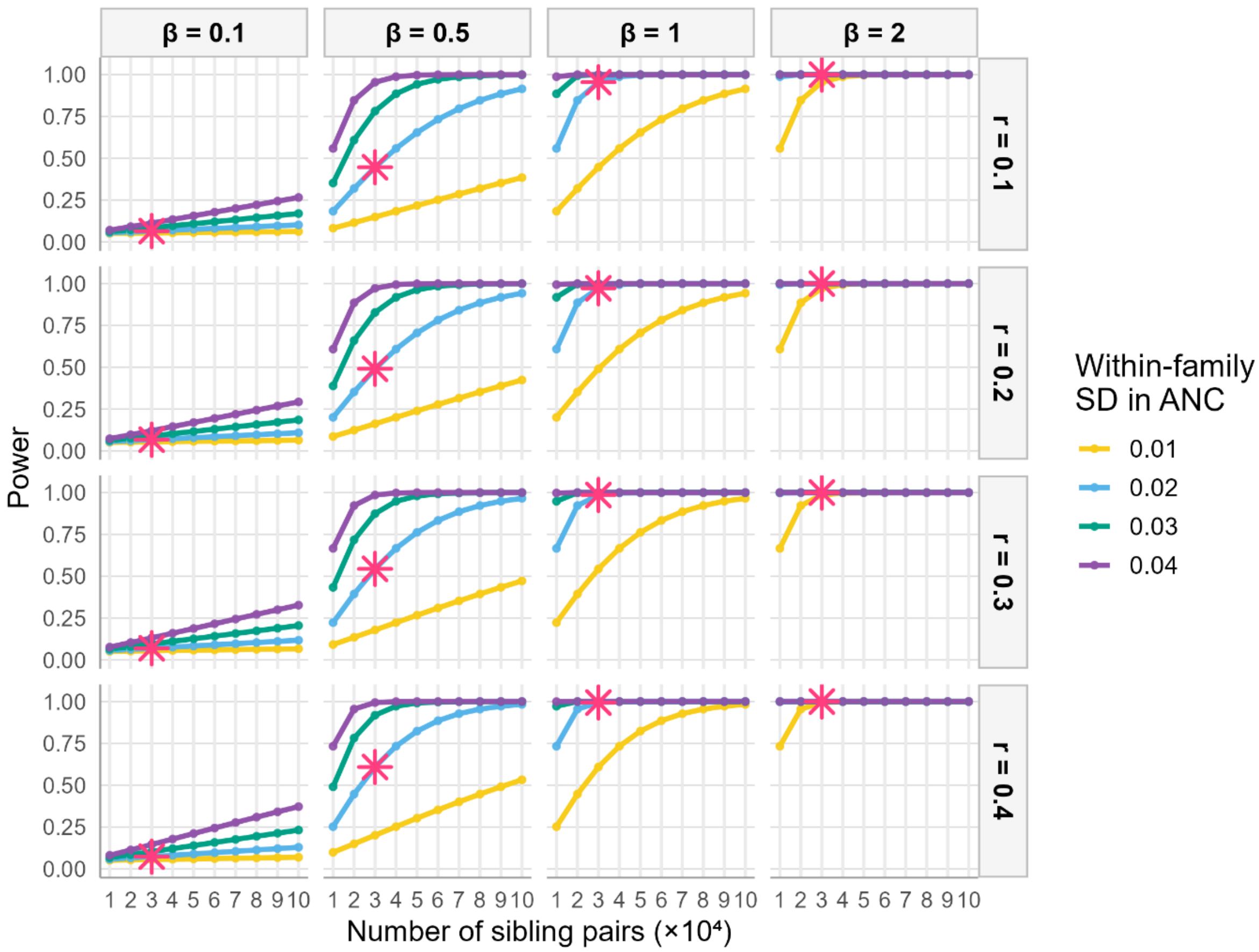

Supplementary Note Figure. Statistical power to detect a within-family ancestry effect.

Statistical power (at a type-I error rate of 0.05) to detect a within-family ancestry effect in a population admixed with two ancestries for a given number of sibling pairs (10,000 to 100,000), sibling phenotypic correlation (0.1 to 0.4), and within-family standard deviation in ancestry proportion (0.01 to 0.04). We have indicated the approximate power of the MCPS study with a red star corresponding to 30,000 sibling pairs and a within-family SD of 0.02. Effect size β is expressed in population standard deviation units.

My final comment, which is more minor, concerns the term "shared environmental variance." The authors are initially clear (p. 7) that what they are estimating is the variance due to effects that are common to siblings but are not explained by IBD sharing, an estimate that might include genetic variance in the population due to assortative mating, but at other points they call this variance component "shared environmental variance." They don't have conclusive evidence that this variance is due to what would be commonly understood as environmentally transmitted effects rather than being due to assortative mating, so I suggest tweaking the language to reflect this ambiguity.

We agree with the reviewer that we do not have conclusive evidence that this residual phenotypic covariance between siblings is due to shared environmental effects and have changed the language accordingly (page 7-8 lines 175-183).

Referee #3 (population genetics):

A. Summary of the key results

Wang et al. use data from the Mexico City Prospective Study (MCPS) to evaluate the contribution of genetic ancestry to complex phenotypes, leveraging the admixed nature of Mexican genomes. They estimate within-family ancestry effects across 15 traits and report significant direct effects of Indigenous American ancestry on height and type 2 diabetes. At the population level, strong associations are observed for educational attainment and several other traits; however, these do not persist once tested in family-based analyses. Despite this pattern being present across traits, the manuscript emphasizes educational attainment rather than presenting a balanced view of the broader set of null results.

We thank the referee for a summary of our work and for the comment about the emphasis on educational attainment (EA). The reason for highlighting EA together with height and T2D is that they each represent a different kind of trait and show a distinctive set of results. Height is often used as a model trait in complex trait genetics and has been studied widely within and between populations, and with very large sample sizes. In our study, it is an example of a trait with strong evidence for a genetic ("biological") between-ancestry difference and evidence that natural selection has contributed to that difference. T2D is an example of a common disease that has been studied widely using gene mapping studies and has a wide range of prevalence among populations. In our study, we show evidence of a between-population difference and that natural selection may have contributed to that difference. EA is an example of a socioeconomic trait that is correlated with outcomes later in life, including disease incidence and longevity. Genetic variation associated with EA has been studied between and within families with large sample sizes, almost exclusively in populations of European ancestry. In our study, we find strong evidence of associations with ancestry between families; however, in contrast to the results for height and T2D, we do not find evidence within families.

Should the reviewers and editors feel that emphasising EA in the Results section is not ideal, we will of course revise the paper accordingly.

B. Originality and significance

The study applies a within-family design to disentangle direct ancestry effects in an admixed population, which is uncommon. While the methodological framework itself is not entirely novel—building on established within-family regression approaches—the adaptation to global ancestry analyses in admixed cohorts represents a useful formalization.

We thank the referee for this nice summary of the novelty.

C. Data & methodology

The MCPS dataset is large and well-suited for these analyses, particularly given the extensive family structure. The methodology is valid and clearly described, but there are important limitations. Analyses rely exclusively on global ancestry estimates; incorporating local

ancestry mapping would considerably strengthen the findings, and the sample size is sufficient to support such analyses.

The definition of educational attainment based on categorical levels rather than years of schooling is problematic, as it reduces comparability across populations and weakens interpretability. Details on the choice of K in the ADMIXTURE analysis should also be added.

We thank the reviewer for these thoughtful suggestions.

Regarding local ancestry mapping (which we understand encompasses admixture mapping and local-ancestry-informed GWAS approaches), we agree with the reviewer that this an interesting line of investigation. However, this would fall outside the scope of this study, as our aim here is to draw inference about genome-wide ancestry effects, rather than mapping individual loci. Moreover, although we do have a relatively large sample size, special care needs to be applied in the presence of extensive genetic relatedness, which is pronounced in MCPS (this is actually the focus of a separate study done in collaboration with the developer of the TRACTOR method, please see this manuscript on medRxiv).

The choice of setting K to 4 in the ADMIXTURE analyses was informed by previous work where we observed that the continental-level, 'ancestral' populations delineated by reference samples, as resolved by studies such as the 1000 Genomes Project (e.g. Auton et al. 2015, Nature) and sometimes referred to as 'superpopulations' (e.g. Fairley et al. 2020, Nuc. Acids Res.), that were most represented within the MCPS cohort were Indigenous American, European, and, to a lesser extent, African and East Asian (see Fig. 3 and Extended Fig. 3 in Ziyatdinov et al. 2023, Nature). As a side note, estimates of global ancestry proportions for IAM, EUR and AFR ancestries obtained from ADMIXTURE and the local-ancestry-based RFMix method were concordant in the Ziyatdinov et al. study (EAS reference samples were not included in the RFMix analysis to improve computational efficiency, as the mean EAS proportion in MCPS was less than 1%). We now provide an explanation for the choice of K within the Methods section (page 31, lines 596-598).

For educational attainment, the way the data were collected in the Mexican City Prospective Study is fixed, so we are unable to modify this aspect. However, we have performed analyses using a transformation of the observed categorical scale to an underlying scale of liability, which is similar to the mapping from education categories to years of schooling that is performed in published GWAS meta-analysis of EA (e.g., Okbay et al. 2022, Nature Genetics; Lee et al. 2018, Nature Genetics; Okbay et al. 2016, Nature). Our analysis shows that the phenotypic correlation between observations on the 1-4 categorical scale and an expanded 14-category z-scale is 0.94 (as described in the Methods section), which implies that our results have not been adversely affected by the scale of measurement. Our scale of analysis is in standard deviation units of educational attainment, which facilitates comparisons across studies.

Appropriate use of statistics and treatment of uncertainties

The statistical models are appropriate, and the derivations clarifying the expectations of regression coefficients are carefully laid out.

However, the interpretation of the educational attainment results overlooks key confounding factors. Sensitivity analyses already show district of residence plays a role; this is expected

given known demographic and educational differences between Iztapalapa and Coyoacán, including school quality and ancestry composition. This entanglement complicates interpretation and should be acknowledged more explicitly.

We thank the reviewer for this comment. We have now provided a more detailed breakdown of the data across these two districts (updated Supplementary Table 2). In our analyses, we fit the districts Iztapalapa and Coyoacán in the model as well as ancestry proportions, and our main analyses are within-families. So effectively the information comes from differences between siblings within families and within districts. We are not sure how entanglement would affect the interpretation but have now mentioned the known demographic and educational differences between the two districts in Mexico City explicitly (page 6, line 128-130).

E. Conclusions: robustness, validity, reliability

The conclusions about height and type 2 diabetes are robust and supported by within-family analyses. By contrast, the conclusions regarding educational attainment are overstated, given the lack of direct ancestry effects and the methodological caveats noted. More caution is needed in communicating these results to avoid overinterpretation.

We thank the reviewer again for the summary of our work and for the comment regarding EA, which we tried to justify in our response to comment C from the reviewer.

F. Suggested improvements

Present a balanced overview of all traits showing population-level associations but no significant direct ancestry effects, rather than emphasizing educational attainment alone.

We thank the reviewer for the comment about a balanced overview. We tried to justify our emphasis on EA in our response to comment C from the reviewer.

Incorporate local ancestry mapping or admixture mapping analyses, which are feasible with the current sample size.

We appreciate and share the reviewer's interest in local ancestry mapping. However, as intimated above, we believe this line of investigation is outside the scope of the current study, as our aim is to infer the effects of genome-wide ancestry rather than map trait loci. Although the sample size in this cohort is relatively large (but small compared to large-scale population GWAS studies which have sample sizes > 1 million), such analyses are not trivial in the setting of extensive family relatedness, which requires special consideration in MCPS where 71% of participants have at least one close relative in the cohort within the 3rd degree (as noted above, this is actually the focus of a separate study, see this manuscript for more details).

Expand secondary analyses beyond coding variants. The current claim that coding variants are "more likely to be causal" is misleading. Non-coding variants are central in complex disease

through regulatory effects; they are simply harder to analyze, and lack of expertise should not be conflated with lack of relevance.

We thank the reviewer for this comment and apologise for the confusing wording. We fully agree that most variants associated with complex traits are non-coding and act through regulation. What we meant by "more likely to be causal" is that, conditional on a variant being statistically associated with the trait, a (usually rare) protein-changing loss-of-function (LoF) or missense variant is more likely to be causal than a common SNP that is statistically associated with the trait. For example, Zheng et al. (2024, Nature Genetics), using a Bayesian mixture model on dense GWAS summary statistics, showed that the annotation of non-synonymous variants increases the probability of such SNPs have a non-zero large effect size. Such effect of non-synonymous variant annotation on the posterior inclusion probability is conditional on all other annotations. Kanai et al. (2021, medRxiv) fine-mapping loci across multiple biobanks and reported a disproportionate number of coding variants having a high (> 0.9) posterior inclusion probability. Huang et al. (2017, Nature) reported a significant enrichment of fine-mapped loci for inflammatory bowel disease for protein-coding changes. The reason is not just that the biological annotation on coding variants is more precise, it is also because LoF and missense variants are less likely to be in linkage disequilibrium with other variants. In contrast, common SNPs have less precise genomic annotation and are typically in LD with many other common variants. We have changed the wording in the text to clarify these points (page 9, lines 207-208).

The authors could attempt to replicate their population-level findings for some traits using data from the Mexico Biobank. It is unclear why this was not pursued.

We thank the reviewer for this suggestion. The purpose of our study was to leverage the family structure in MCPS to estimate within-family effects, not to estimate population-level effect sizes of ancestry proportions per se (the population-level analyses served as a comparator to our within-family analyses). Such studies have been done before (Lin et al. 2024, Genome Med; Asgari et al. 2020, Nature; Moreno-Estrada et al. 2014, Science; Kumar et al. 2010, N Engl J Med; Florez et al. 2009, Diabetologia), and typically show statistically significant associations with genetically-defined ancestry. Without large-scale family data to estimate within-family ancestry effects, it is difficult to see what a 'replication' of the population-level effects would add.

H. Clarity and context

The manuscript is generally well written and the statistical derivations are clearly presented. However, the abstract and conclusions currently give disproportionate weight to educational attainment, which could mislead readers. Greater balance in highlighting both significant and null findings would improve clarity and context.

We are grateful to the referee for the additional comment regarding educational attainment. This issue has been addressed in our response to Comment A.

I verified that the code and documentation are available. However, I was unable to run the code on the platform. The documentation lacks a user manual for individuals not directly involved

in the project. While the inline comments are helpful, a standalone manual would greatly facilitate broader usability.

We thank the reviewer for the thoughtful review. The original dataset cannot be shared on the platform due to data confidentiality restrictions. To assist with the evaluation, we have generated synthetic random-number data that follows the same structure as the original dataset. This allows the code to run and demonstrates the workflow without compromising restricted information. An expanded README file has been provided. We would be happy to provide any additional clarification that may be helpful.

We sincerely thank all three reviewers for their continued time and insightful comments in this second round of review. We greatly appreciate their careful evaluation of our revised manuscript and their constructive suggestions, which have further helped us to improve the quality, clarity, and overall presentation of our work. We have carefully considered all comments and have revised the manuscript accordingly. Below, we provide a detailed, point-by-point response to each of the reviewers' comments. We have also taken the opportunity to make very minor changes to wording and presentation to enhance clarity. All changes are highlighted in the manuscript (in red font).

Referee #1 (Remarks to the Author):

I thank the authors for their revised manuscript, which greatly improved upon the messaging and clarify. My comments mainly revolve around the positioning of this work, including softening some language and explaining why this matters within the field. While the authors did respond within the "response to reviewers" document, much of this information is not adequately translated into test within the manuscript. While I realize some of my comments are repetitive with my prior comments, it is still worth stating and improving upon in the revised version. For what it's worth, I found the response to reviewer comments much clearer than the manuscript for many of these points, so I hope it's just a matter of changing language/framing. I've organized my comments below in terms of major comments, minor comments, and figure-related concerns.

MAJOR

1. I would like to see a better justification for these analyses given current discussions on the role of genetic ancestry and environment in disparities. The introduction sets it up with thinking generally between genetic and environmental differences, but the paper is focused on *genetic ancestry*. Clarification is needed for how these are connected and why this matters. (lines 70-75) I see the added line at the end, but this is not tied to a larger question as to why this study matters in the field.

We thank the reviewer for this comment. We agree that the motivation for focusing on genetic ancestry should be more clearly connected to the broader question of genetic and environmental contributions to phenotypic differences. We have revised the introduction with the following sentences. "Here, ancestry proportions are first inferred from genotype data, and we then apply the same underlying logic to estimate the differing effects of

genome-wide ancestry from different ancestral populations within families." (page 4, lines 83- 85).

(The text at the end of this section (lines 85–90) was added based on feedback from a previous round of review.)

2. Additionally, why is MCPS is a good study for this? The introduction should have a background of ancestries and how that interacts with the environmental differences. This would require the authors to draw upon the epidemiology of the traits in Mexico City and how that intersects with social stratification and genetic ancestry. The results are compelling, but the current iteration lacks the "why" in the introduction and the "so what" in the discussion. This should also be included in the abstract, where right now it jumps from GxE to ancestry without a connection.

We thank the reviewer for this comment. We agree that the motivation for focusing on the MCPS cohort and its relevance to the broader question could be more clearly articulated. In response, we have revised the Introduction as follows.

"For example, in Mexico, obesity[17] and T2D are highly prevalent and associated with ancestry[18], but the pathways underlying this association remain unclear." (page 3, lines 75-77).

"With its large sample size, substantial genomic ancestry variation, and enrichment for related individuals, the Mexico City Prospective Study (MCPS)[3,4] enables the quantification and dissection of associations between ancestry and complex traits." (page 4, lines 91- 93).

2.1. Lines 303-305, about how these associations could lead to misleading inference, is absolutely vital and should be hammered home much more often in the introduction and the results section. I know different folks have different feelings about this, but I think a bit of interpretation in the results section would be very helpful to ensure this paper is not misunderstood with a general audience.

We thank the reviewer for this useful suggestion. We agree that the potential for misleading inference from population-level associations is an important point. In addition to the discussion section highlighted by the reviewer, we note that this issue is already introduced earlier in the introduction part (page 3, lines 71–73) and results part (page 7, lines 159-161). We have further revised the text to reinforce this point and improve clarity for a general

audience. "However, these associations do not distinguish genetic effects from social and environmental factors correlated with inferred genetic ancestry." (page 7, lines 159-161). We still have an entire paragraph in the Discussion about the possible mechanism of within-family associations with inferred genetic ancestry (page 14, lines 317-333).

3. Throughout the piece, I would soften the language to be less absolute. For example, consider rephrasing "estimating unconfounded" in line 36 with something not quite as strong, such as "addressing confounding" or "while controlling for between-family confounding". 3.1. In line 77, you could change to "therefore associations between within-family genotype variation and phenotype are (*assumed*) to be due only to the causal effects of inherited alleles." This is consistent with the note at the end of the paragraph, in which differences can be due to mediation.

We thank the reviewer for this thoughtful suggestion. We have revised the manuscript to adopt more cautious language throughout and avoid confusion. Specifically, we have replaced the sentence that contained "estimating unconfounded" with "Family-based analyses of genetically admixed individuals offer a framework for estimating ancestry effects that are not confounded by ancestry-environment correlations" (page 2, lines 35-37). For the second sentence mentioned by the reviewer, we have changed it to read "therefore associations between within-family genotype variation and phenotype are expected to be due only to the causal effects of inherited alleles" (page 4, lines 81-82), because even if the mechanism is due to mediation, inherited alleles are the cause. We have revised the manuscript to ensure the tone is more accurate.

3.2. Line 343: caution when using the terminology of causality. There are specific criteria to fulfill according to many causal inference frameworks, and it is not clear that these are fulfilled in this study design. There have been other studies demonstrating that there is confounding in aggregation between ancestry proportions and outcomes that is eliminated with granular labels, but this study is important as it looks at family structures. (You also claim to be the first to do this, but later (line 369 and on), there is a description of a similar study in Greenland families. (I do not think you need to be the first to be important, so would remove that language entirely.))

We thank the reviewer for this thoughtful comment. We agree that careful use of causal language is important, and we have revised the manuscript to remove the term "causally"

from the sentence (page 16, line 365) to avoid potential misinterpretation. However, we respectfully maintain that our within-family design provides a well-established basis for causal inference under standard genetic epidemiology frameworks because it is based upon Mendelian randomisation within families. Accordingly, we have not revised other instances referring to causal effects, as these are consistent with the assumptions and interpretation of the study design. Our approach leverages the random segregation of genetic material during meiosis, which reduces confounding from shared environmental and ancestral background. Under standard assumptions, this allows within-family ancestry–phenotype associations to be interpreted causally, although we clarify that this does not imply a specific biological mechanism.

We agree that population-level associations between ancestry and outcomes may be confounded by environmental factors. For example, we find that several strong associations observed in the population sample—particularly for educational attainment—are not supported within families, underscoring the value of family-based designs.

We appreciate the reviewer's point regarding novelty. We acknowledge the important prior work in Greenlandic families (Lin et al.), which applied a within-family framework to metabolic phenotypes. We clarify that our "first" claim (page 16, line 365) refers specifically to disease outcomes, as our study provides, to our knowledge, the first within-family evidence of ancestry-related genetic differences in susceptibility to a common complex disease (type 2 diabetes).

4. In your discussion, how do these results compare to previous results? What is new? Association with height is already known, as well as differences in metabolic traits. What is *new*, such as the comparison between these effect estimates and prior literature, should be highlighted.

We thank the reviewer for this question. Apart from the study in Greenland (which we cite in the Abstract), all associations between ancestry and complex traits reported in the literature are at the population level, for example, the association between ancestry and height in a sample from Peru (Asgari, S. et al. Nature,2020). The association between height and ancestry within families reported in the Greenland study is statistically not significant (estimate of -3.5 cm, SE 6.8 cm), as we reported (page 17, lines 396-397). Therefore, even for the model trait height, there is no prior evidence of associations with ancestry that are not confounded by environmental and social effects between families. In contrast, we show a strong robust association of height with genetically inferred ancestry within families, show that the effect size attenuates when a count of height-increasing alleles is fitted in the

model and provide evidence that the height-increasing alleles are more differentiated between Indigenous American and European ancestries than expected under drift. Therefore, there is no prior study that shows any robust evidence that the observed phenotypic associations at the population level is mostly due to genome-wide genetic effects of height-associated loci. Furthermore, using our more stringent approach to multiple testing than the FDR approach in the Greenland study (page 17, lines 398-399) would imply that none of the reported associations in that study for metabolic phenotypes (based upon 697 families) are statistically significant with that threshold. We report, to our knowledge for the first time, a within-family association between ancestry and disease (page 16, lines 364-366). We believed that we had already articulated the novelty of our approach and findings but have now added a few sentences in the Discussion. "Our study provides both a template on how to perform robust ancestry associations on complex traits (including disease) with family data whilst modelling the covariance between siblings, and provides power calculations that identify the sources of information that determine power and quantifies future sample sizes needed to perform these analyses in different ancestries." (page 13, lines 297-301).

5. The language when referring to ancestries does not follow current guidelines regarding the distinction of inferred ancestry based on reference populations versus some "truth", which cannot be ascertained. For example, line 97 could be altered to say "Genome-wide proportions of *inferred* IAM..." followed by stating the reference populations. This makes it clear which reference groups were used (TGP and HGDP) and that these are relative to those groups.

We thank the reviewer for this helpful suggestion. We agree that it is important to clearly distinguish inferred ancestry from any notion of a fixed or "true" ancestry. In response, we have revised the manuscript to explicitly refer to "inferred" ancestry where appropriate (page 4, line 98; page 5, line 106, 112, and 114; page 7, line 161; page 16, line 379) and to clarify that these estimates are defined relative to the reference populations used (page 5, lines 107-109). We have implemented these changes throughout the manuscript to improve clarity.

5.1. Justify the use of 100% EUR baseline in the main results. Why is EUR the standard? There are different reasons others use, such as it is the most common ancestry (not relevant for this population) or they care comparing results to previous findings, which were primarily done in EUR groups. I am completely fine with its use and how it was defended in the response to

reviewers! But a line or two as to why it was chosen, not as a default for humans but for this specific analysis, would go far.

We thank the reviewer for this suggestion. We have added the following clarification to the Methods section. "Ancestry association analyses were conducted with EUR ancestry specified as the reference. IAM and EUR ancestries together account for the majority (> 90% on average) of genome-wide inferred ancestry in this population, and model identifiability constraints permit the inclusion of only three of the four ancestry components. Retaining IAM in the model therefore enabled a more interpretable result in this study population where genetically inferred IAM ancestry comprises the largest proportion." (page 36, lines 715-720).

6. What was the correlation between a matched set of alleles for the traits? It is expected that due to genetic drift, a large proportion of GWAS SNPs will be positively correlated due to chance and not specifically from the trait. An appropriate comparison would be a matched trait-agnostic set of similar frequencies. Also, functional alleles are more likely to be common and therefore shared across ancestries. Again, the comparison for attenuation should be done with a different set of alleles that are matched by frequency to make a stronger case for these specific alleles being important. You could even use the SNPs matched in the later section for this analysis, so hopefully it isn't a big lift.

Follow-up clarification in email:

"This was pertaining to their analyses looking at coding variants (page 9, lines 209-214). My comment was pertaining to the link between saying that the coding cTIA (missense and LOF) are more likely to be causal (which I don't debate) and then using the cTIA to see if the effects are attenuated. The concern is that coding variants are more likely to be common compared to a random distribution of variants that are trait-increasing (or trait-agnostic). This can be seen with the relatively few number of T2D cTIA, which they attribute to low frequencies.

In order to say that the attenuation is due to cTIA (and not just common TIA), they would need to do the same analyses with frequency-matched non-coding variants. Swapping out the outcomes (using T2D for the height analyses) does not do this as there are such fewer variants and at lower frequencies, so it of course will not have the same signal.

Later in the manuscript they do have some frequency-matched variants (page 10, lines 243-246), although this is looking at frequency differences between IAM and EUR, so maybe they could adapt those scripts?"

We thank the reviewer for the question and follow-up clarification (copied and pasted from what we received from the Editor). We were still not entirely sure what the reviewer was asking and hope that we have interpreted the question(s) correctly. We note that the "c" in cTIA does not stand for "coding" but for "count", and have emphasised that in the main text.

Genome-wide ancestry proportions in admixed populations can be correlated with linear combinations of SNP dosage because any SNP is, on average and irrespective of trait association, informative on ancestry if the ancestral populations differ in allele frequencies. We quantified the robustness of our associations between ancestry and the count of trait-increasing alleles (cTIA) and the attenuation of the within-family estimate of ancestry when fitting trait-specific cTIA by randomly permuting the sign of the increasing allele label at associated loci multiple times. We performed these analyses for height and T2D using both the full set of associated SNPs and the set of LoF/missense variants.

We find that the reported partial correlations between IAM ancestry and height and IAM ancestry and T2D are statistically significant when performing this control analyses (P = 0.001 and 0.006, respectively, from 1000 permutation analyses). For the 314 LoF/missense variants associated with height, the reported partial correlation of -0.25 with IAM ancestry is marginally significant (P = 0.055). None of the results are significant for the 36 missense variants associated with T2D. Results are summarised in Rebuttal Fig. 1 and 2.

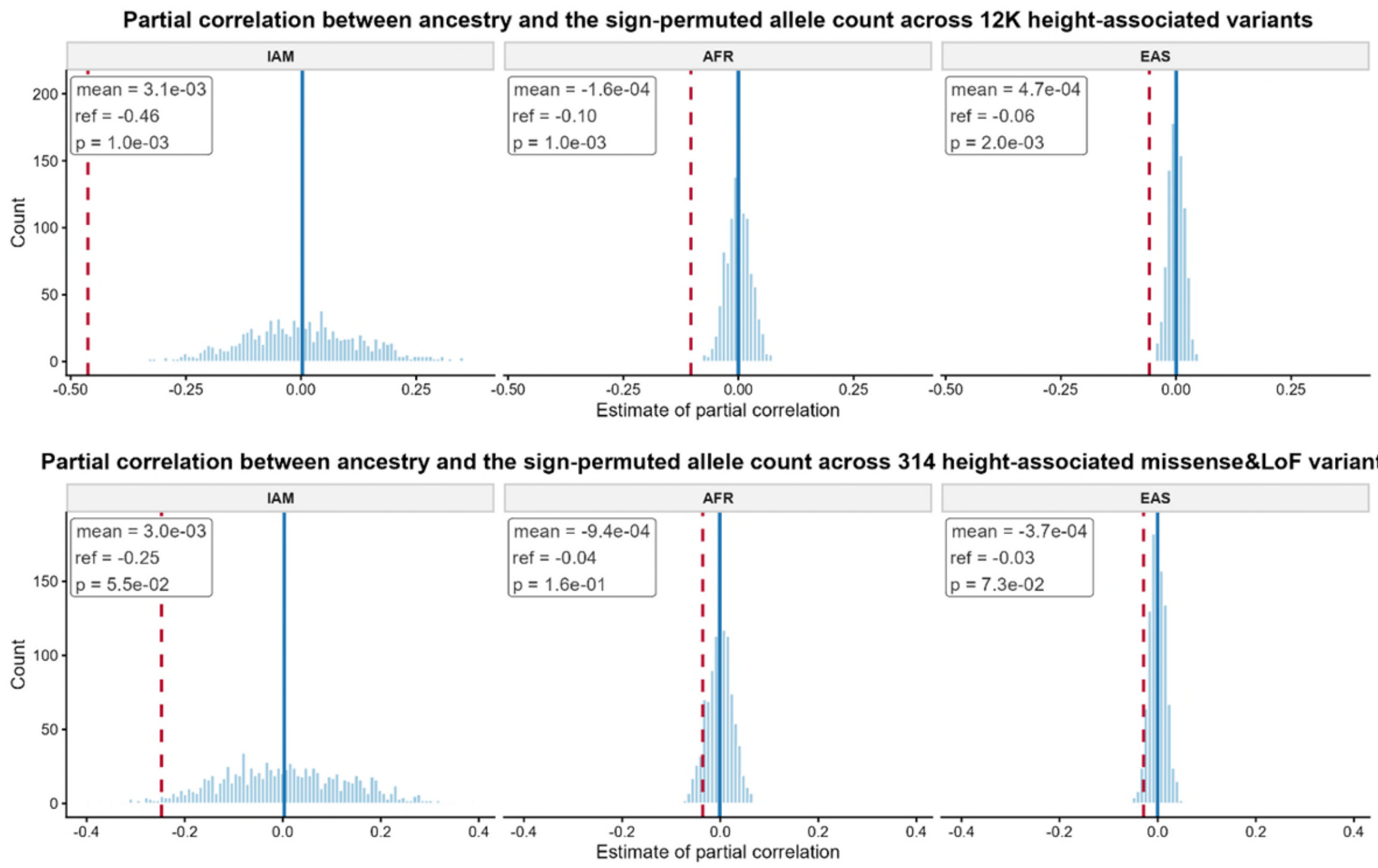

Rebuttal Fig 1. Partial correlation between ancestry and the sign-permuted allele count across 12K height-associated SNPs (top) and 314 height-associated missense & LoF variants (bottom)

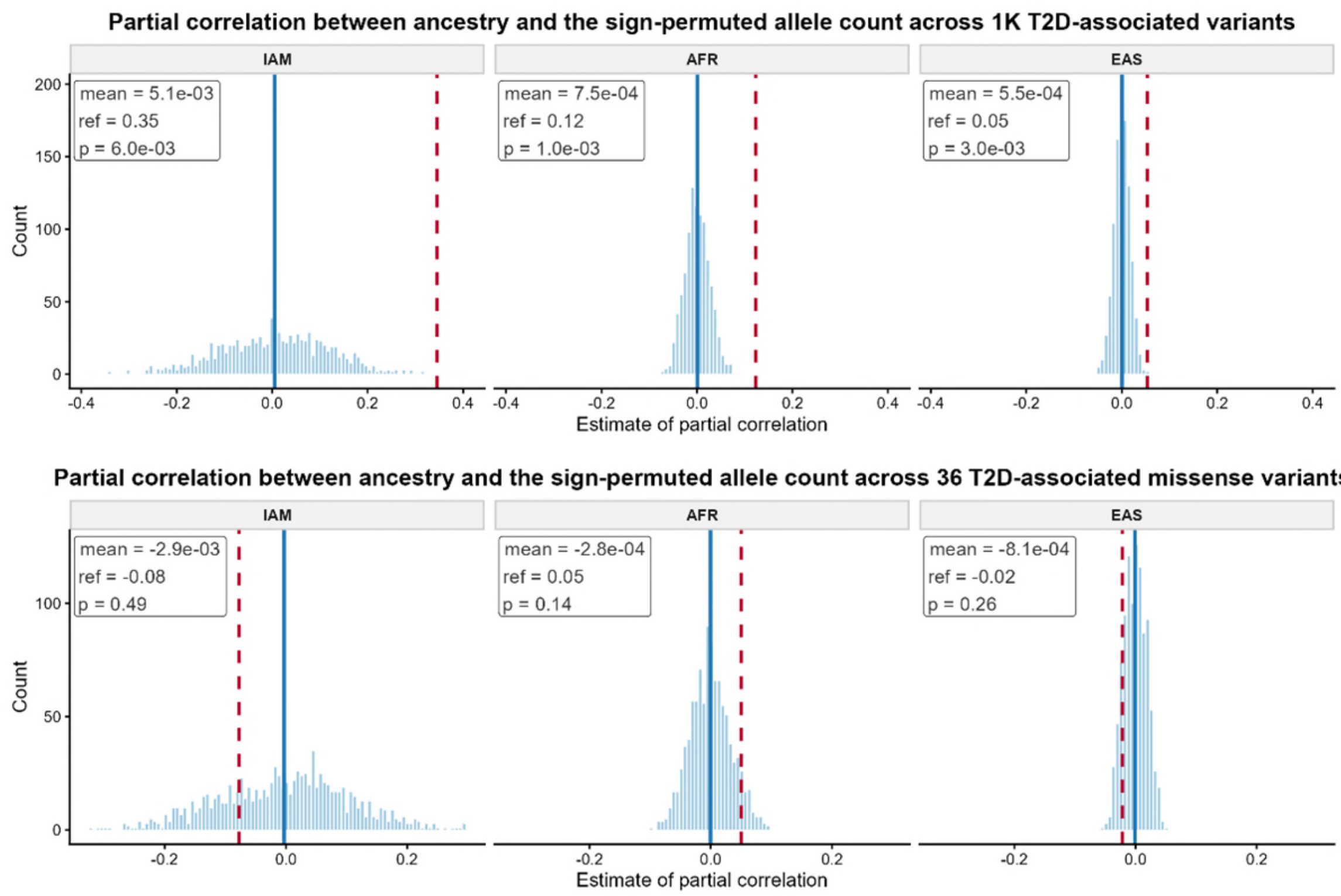

Rebuttal Fig 2. Partial correlation between ancestry and the sign-permuted allele count across 1K T2D-associated SNPs (top) and 36 T2D-associated missense variants (bottom).

We next performed the within-family analyses of trait association with ancestry, using 100 iterations of permuted counts. For T2D, we used the 0-1 scale for analyses. As shown in the following figures, fitting the randomised allele counts in the model has a negligible effect on height or T2D and do not influence (attenuate) the effect size of ancestry on the phenotypes.

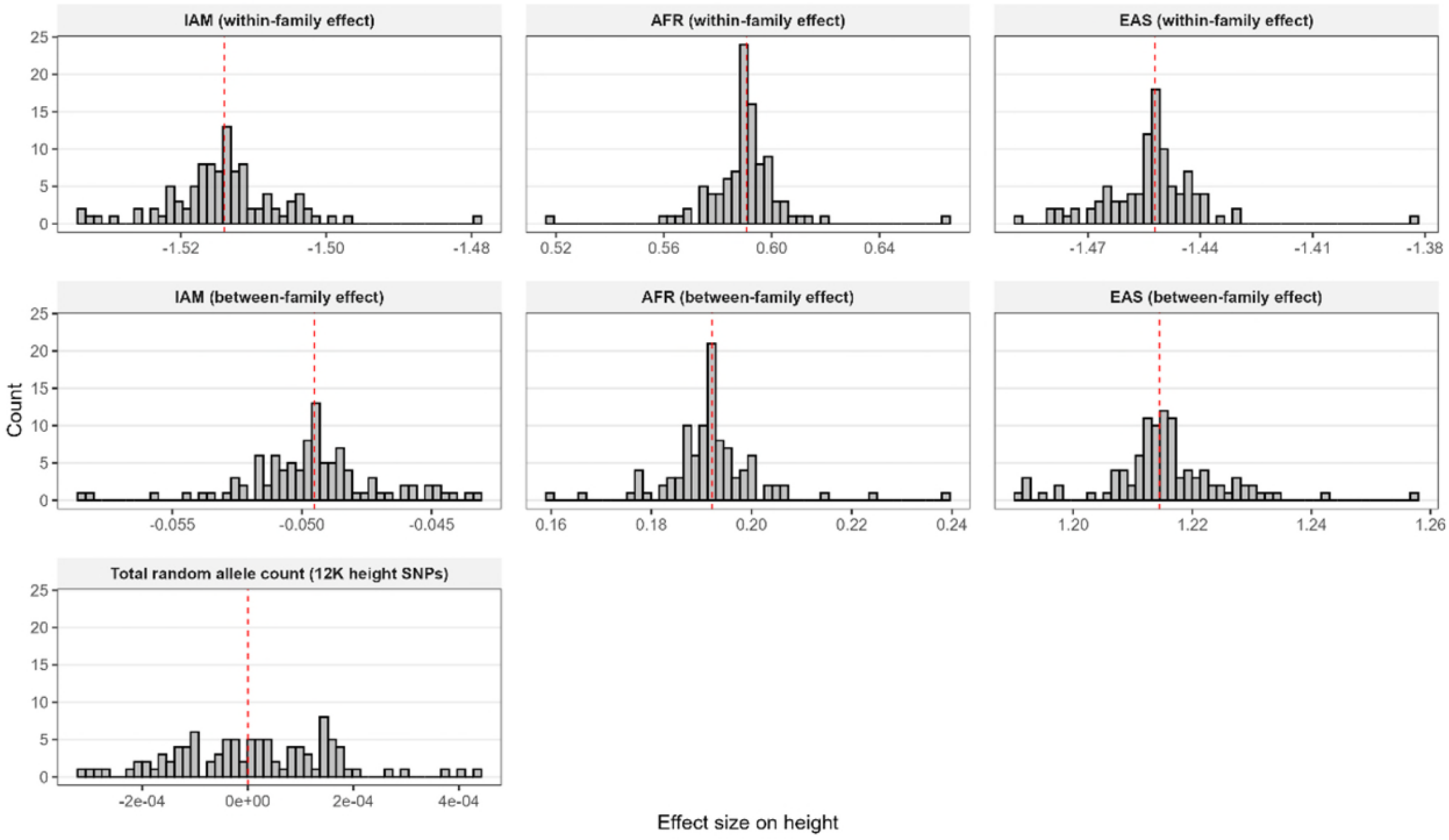

Rebuttal Fig 3. Effects of ancestry and allele count on height from sign permutations at 12K height-associated loci.
The histogram illustrates the distribution of effect size estimates from the REML analysis. By incorporating random allele counts—derived from 100 sampling iterations—into the model, we estimate the effect of genetic ancestry on height within families. The red dashed line for ancestries indicates the reported effect of different ancestries on height without the random allele count in the model, and that is the effect size we reported in the main manuscript. The red dashed line in the random allele count plot represents 0.

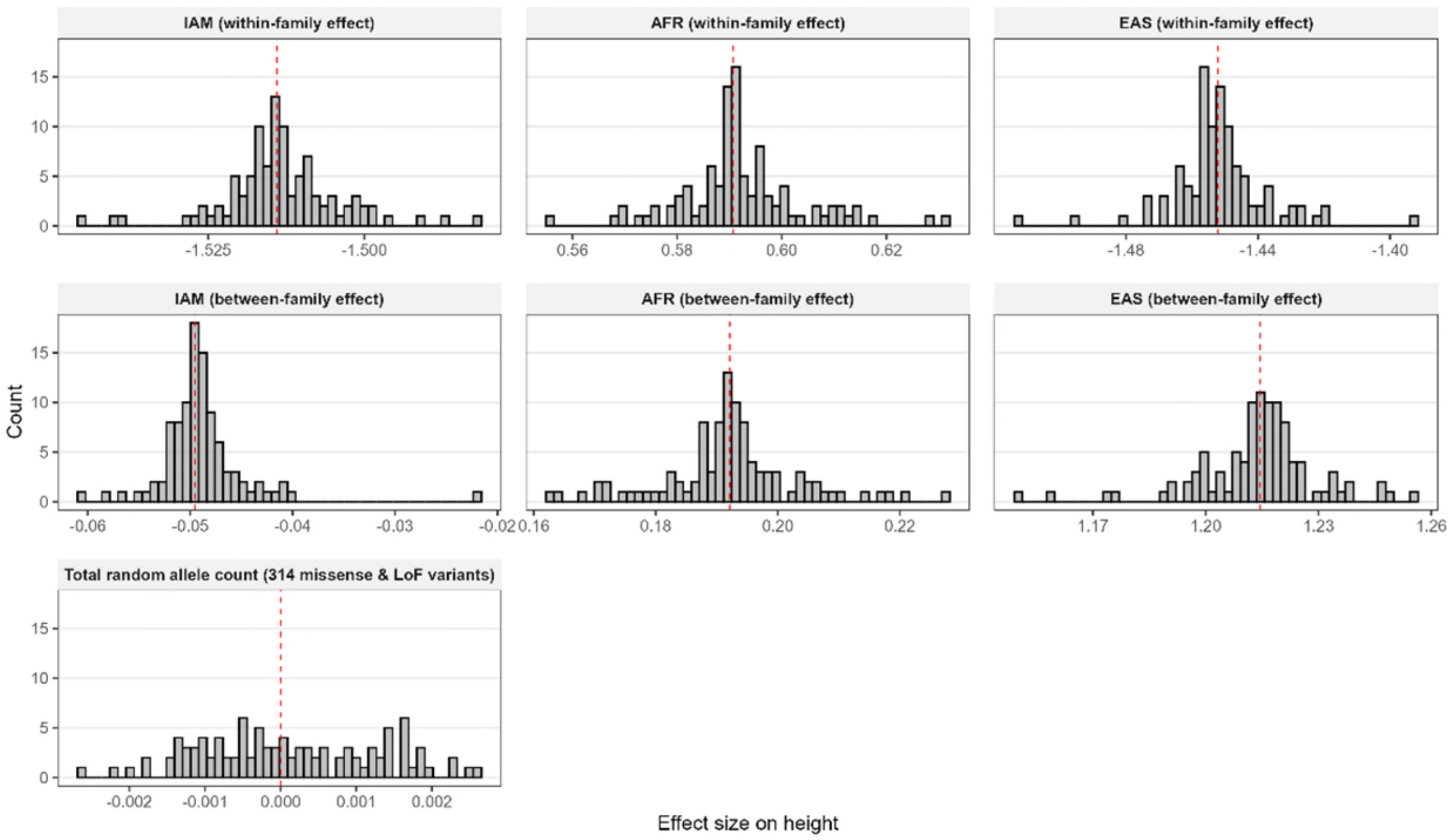

Rebuttal Fig 4. Effects of ancestry and allele count on height from sign permutations at 314 height-associated LoF/missense variants.

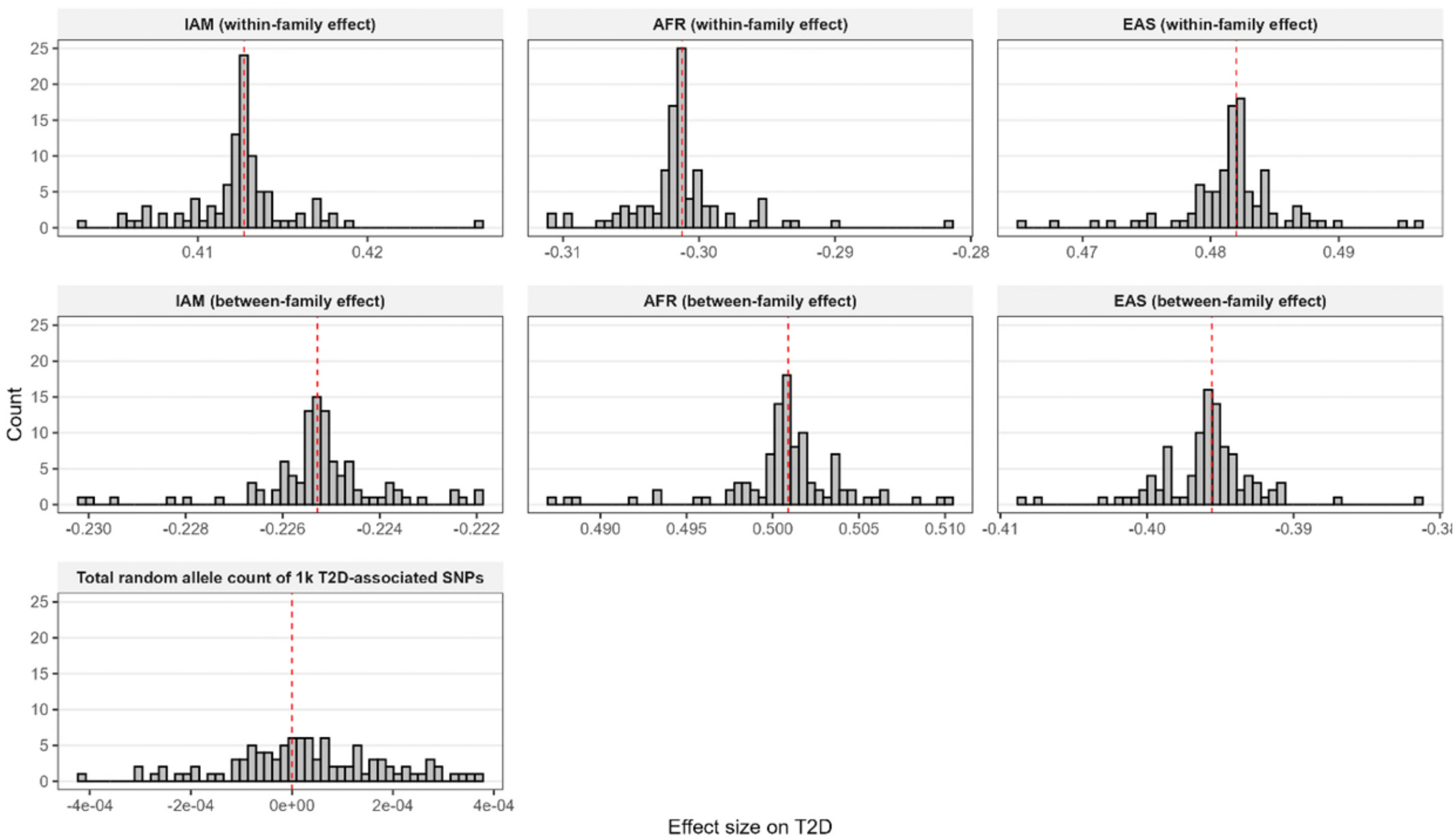

Rebuttal Fig 5. Effects of ancestry and allele count on T2D from sign permutations at 1K T2D-associated loci.

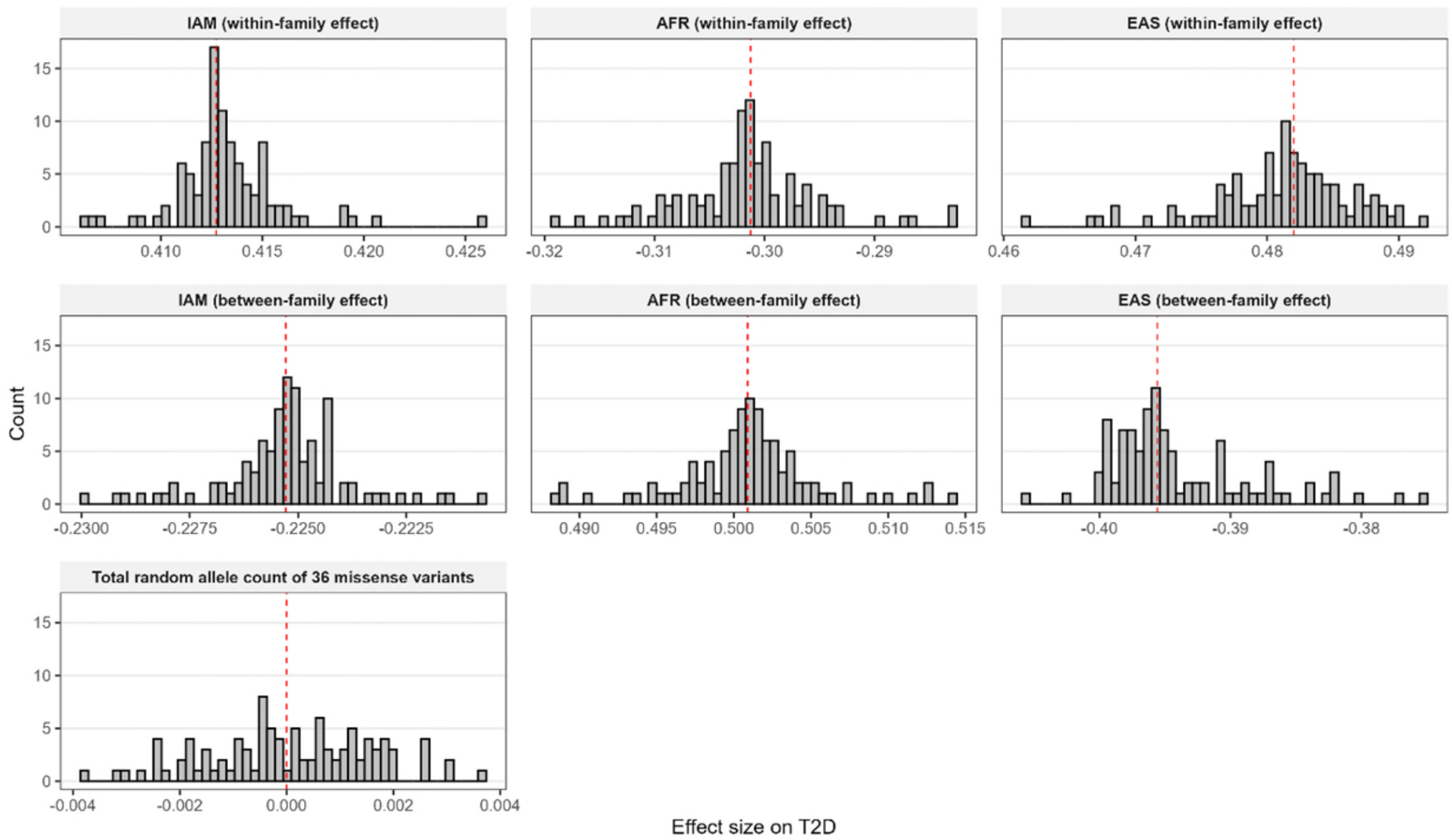

Rebuttal Fig 6. Effects of ancestry and allele count on T2D from sign permutations at 36 T2D-associated missense variants.

Taking these results together, they are consistent with what we reported in the manuscript. The reported partial correlations between trait-increasing allele counts and ancestry are trait-specific, and fitting permuted counts of trait-increasing alleles in the within-family trait analysis does not attenuate the effect size of ancestry, whereas fitting the non-permutated counts attenuates the signal.

We have added a sentence in the text about the permutation test for the partial correlations between genome-wide ancestry proportions and cTIA, "The observed partial correlations between cTIA and ancestry proportions remained statistically significant for the full set of associated variants when compared to random permutations of the label of the trait-increasing allele at each associated locus (Methods), but not when considering the smaller set of coding variants (Supplementary Figure 6)." (page 10, lines 227-231). We have also included a new figure (Supplementary Figure 6) that combines Rebuttal Figures 1 and 2.

MINOR

7. Change lnOR to odds ratio to aid in interpretation. (Is the log odds really 5.13? That seems huge! If this is actually the OR, that would be more consistent compared to the unrelateds analysis for T2D.)

We thank the reviewer for this comment. We confirm that the reported values are natural log odds ratios (lnOR), not odds ratios. As discussed in the manuscript, although the within-family estimate is extremely large, so is its standard error and the large magnitude is likely

overestimated due to the winner's curse (selection bias). We have clarified this point to aid interpretation (page 15, lines 355-359). We note that our estimate of the within-family effect size from the linear mixed model (on the 0-1 scale) is also very large (0.41, SE=0.12), implying an approximately 41 percentage-point increase in prevalence between 0% and 100% IAM ancestry.

8. Include estimates of precision for correlations in results (line 112).

We thank the reviewer for this suggestion. We have revised the results section to include the corresponding confidence intervals (page 5, lines 122-123), to improve clarity and interpretability.

9. How was the increase of T2D prevalence estimated at 19%? This may have been detailed somewhere in the supplement (if so, I apologize), but it would be helpful to make it clearer in the results in terms of how you got from OR to prevalence differences.

We thank the reviewer for this helpful comment and appreciate the opportunity to clarify the original text. The reported approximately 19% increase refers to an absolute difference in T2D prevalence (19 percentage points), which was estimated using the coefficients from the multiple regression linear model with T2D as the outcome (Supplementary Table 3, line 20). This estimate was therefore derived directly from the linear regression coefficient, rather than from the odds ratio obtained from the logistic regression model. We can also obtain an approximation from the logistic regression estimate (=1.73), the sample mean prevalence (= 0.138) and sample mean IAM proportion (=0.65), which yields an approximate increase of ~18 percentage points, highly consistent with the linear model estimate. We have detailed the calculation in the Methods (page 37, lines 737-745).

10. The limitations for sample sizes could be partially addressed by redoing one of the analyses with a smaller sample of unrelated individuals. Additionally, if you estimated power, just say the actual numbers in the text (don't make me track down supplementary note 2 in order to understand how big of a limitation this is). It seems low based on the figure, but how low is it?

We thank the reviewer for this comment. We are not sure how downsampling unrelated individuals would help to clarify or quantify sample size limitations. The limitation of the sample size to estimate within-family effect sizes of ancestry refers to the number of families and we have quantified in the power calculations (Supplementary Note 2) how the sampling

variance of the estimate of within-family effect scaled with sample size. We have reported the power of our analysis in the main text and now explicitly refer to the corresponding Supplementary Note figure, making the details easy to locate (page 14, lines 331-333).

11. [This is not important for this manuscript per se but would be interesting to do in future work.] Do individuals identify themselves in terms of "ethnicity" or "group" within Mexico? How does these results within those self-identified groups? And do the results differ by district? I'm not as familiar with the geography and history of Mexico City, but considering the population substructure present in these participants, I wonder if the results would differ by district or by how people identify themselves (if that information is available).

We thank the reviewer for these helpful suggestions. We agree that examining results by self-identified ethnicity or geographic substructure would be informative. However, such information is not currently available in the MCPS dataset. Geographic variation is partially accounted for by including district as a covariate in our models. We acknowledge that social identity and finer-scale geographic context may capture additional environmental and socio-cultural factors, and we consider this an important direction for future work.

FIGURES

12. Figure 2 may be more impactful as a heat plot showing direction of effects. Also, I would suggest changing the title to be specific to unrelated individuals and the legend to include the limitations. However, depending on the authors and editorial team, if there is a concern that this data could be used to further race science, I would even suggest removing this figure entirely as Figure 3 gets the point across more effectively with less chance of misinterpretation.

We thank the reviewer for these helpful suggestions. In response, we have removed the previous Figure 2 to reduce the risk of misinterpretation and use Supplementary Table 3 to show the results of this part.

13. Is there a way to make Figure 4 more distinct from Figure 3? Perhaps to show how many of them have null associations? This could include making null associations in dashed lines and the non-null ones in bold? Just to make it more visually striking and get the message across as opposed to a data dump.

We have revised previous Figure 4 (current Figure 3, page 28) to enhance clarity and to better distinguish it from previous Figure 3 (current Figure 2, page 26). Specifically, null and

non-null associations are now differentiated using red asterisk symbols, making the overall pattern of results more readily interpretable. We also drew a figure with dotted lines to indicate non-significant results following the reviewer's suggestion (see below). However, this distinction was not very visually clear in the figure. Since statistical significance is already explicitly indicated by red asterisk symbols, we believe this provides a clearer and more interpretable presentation.

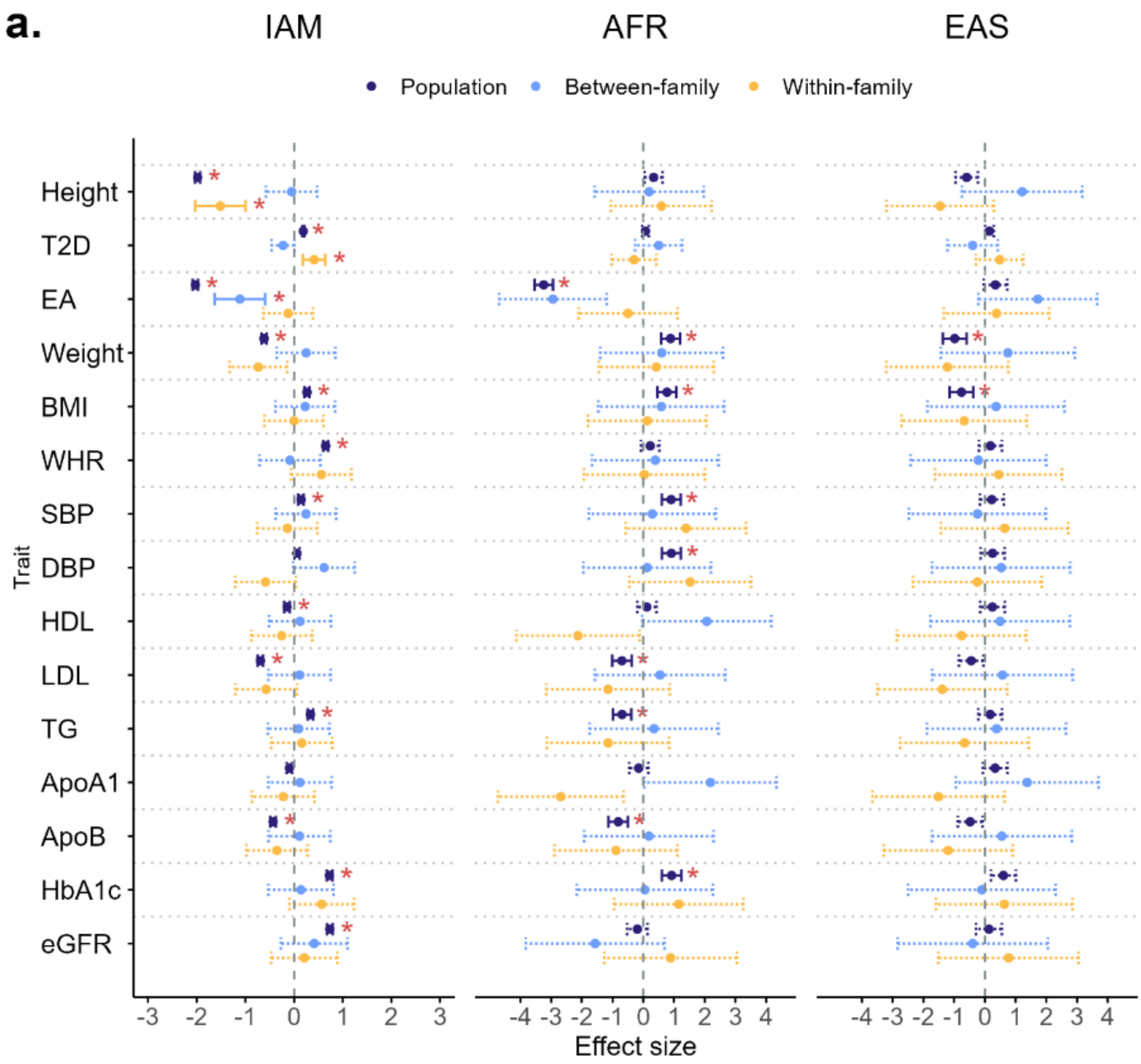

Referee #2 (Remarks to the Author):

The authors responded to my previous critiques, and I have no further major concerns.

Given the topic of this paper & the relative novelty of its approach, I anticipate that it might attract considerable attention within and beyond the academy. In the interest of communicating the paper's objectives and results as clearly as possible, I want to flag the following sentences for review by the author/editor, as they might imply, especially to a general reader, the existence of an overly simple nature vs. nurture binary.

We thank the reviewer for these helpful suggestions and agree that for each of the highlighted sentences the wording can be improved.

"but the extent to which differences are caused by genetic or environmental factors is unknown for most complex traits" --> could be simplified to just "but the extent to which differences are caused by genetic factors is unknown for most complex traits"

Revised accordingly (page 2, line 32).

"estimating unconfounded effects of genetic ancestry on phenotypes" --> "estimating effects of genetic ancestry on phenotypes that are unconfounded by environmental differences between families"

We have changed the wording to "Family-based analyses of genetically admixed individuals offer a framework for estimating ancestry effects that are not confounded by ancestry-environment correlations" (page 2, lines 35-37).

"the relative contribution of genetic and environmental factors, and their interactions, to these differences. In this study, we combine large scale data and a gold standard approach to address this question." --> You're not really quantifying the contribution of environmental factors or genexenvironment interactions here. could simplify, again, to just "the relative contribution of genetic factors to these differences".

Revised accordingly (page 3, line 67).

"However, these associations do not distinguish between within-family ancestry effects and environmental effects that are correlated with genetic ancestry proportions" --> suggest extending this sentence to say "environmental effects that are correlated with genetic ancestry proportions between families"

We have changed the wording to "However, these associations do not distinguish genetic effects from social and environmental factors correlated with inferred genetic ancestry" (page 7, lines 159-161).

Referee #3 (Remarks to the Author):

The manuscript has clearly improved in response to the reviewers' feedback, and the tone is now more cautious and responsible. I appreciate the additional care taken to frame the results in a way that supports careful interpretation by the community.

We thank the reviewer for their positive assessment of the revised manuscript. We really appreciate that the reviewer's previous comments have contributed to the improved tone and the more cautious framing of the results.

Referee #3 (Remarks on code availability):

The provided README files and simulation data ease usage of the code and methods for the community.

We appreciate the reviewer highlighting the usefulness of the provided README files and simulation data, and we are glad to see that this facilitates the use by researchers.

Referee #1 (Remarks to the Author):

I thank the authors for their responsiveness to feedback and this improved version of the manuscript. The clarifications throughout the manuscript greatly helped with contextualization and interpretation, as well as the additional methodological details.

While I disagree with the authors use of causality, even drawing from Mendelian randomisation in which certain assumptions need to be met (which I do not think this study design can do). My understanding of mechanistic causality is in contrast to that of unconfounded association, under which I would place this study design. The associations here are examining how ancestry may correlate with certain complex diseases, not that the AIMs are directly related to the biology of that trait. However, I think this is one of those philosophy of epidemiology "agree to disagree" moments and do not think it requires any additional revisions before publication.

We thank the reviewer for this thoughtful comment, including the "agree to disagree" (or disagree agreeably) comment. We note that the reviewer uses the phrase "mechanistic causality", which is not a term we are familiar with. Our interpretation of what the reviewer means is that it applies to a situation where X is known to cause Y <u>and</u> it is known how (mechanistically) X causes Y. Taken literally, this definition would exclude even a randomised controlled trial (RCT)—the gold standard for causal inference—if the mechanism by which the exposure (e.g., a therapeutic) affects disease outcome were unknown.

We appreciate and respect the reviewer's perspective and have revised the manuscript to clarify our use of causal terminology while preserving the intended interpretation. The revisions are detailed below.

- ***"Within-family genetic variation is due to random segregation of parental alleles during meiosis, therefore associations between within-family genotype variation and phenotype are expected to be due only to the causal effects of inherited alleles[21–23]." (page 4, lines 94-97)***
- ***"In summary, our study shows that within-family analyses of admixed families can be used to estimate the effects of genetic ancestry on complex traits and diseases. Using data from the MCPS, we found evidence for a within-family effect of Indigenous American ancestry on height and risk of type 2 diabetes relative to European ancestry, and that these ancestry differences were shaped, at least in part, by natural selection." (page 17, lines 428-433)***
- ***"Here, ancestry proportions are first inferred from genotype data, and we then apply the same underlying logic to estimate within-family effects of genome-wide ancestry." (page 4, lines 97-99)***

- ***"We exploited the random segregation of parental chromosomes during meiosis to estimate within-family effects of genetic ancestry differences on complex traits in an admixed sample from Mexico City." (page 12, lines 297-299)***
- ***"In principle this design can be applied whenever there is phenotypic and genome-wide genetic data from admixed families, enabling the separation of within-family ancestry effects from population-level correlations between genetic ancestry and phenotype due to other factors." (page 12, lines 299-303)***

A very very very minor point, mainly to set the tone for the piece, is to remain the word "species" at the beginning of the manuscript. Perhaps "Within humans, multiple...". And then you can remove the "In humans" part of the third sentence. While there isn't anything wrong with the original, I think it will better center the people in this study, especially since it is likely to generate some discussions. And then adding a "can" on line 65 ("Human populations *can* differ in phenotype means..."). I know that I sound like a broken record, but these things can help pre-empt some responses so that the rest of the manuscript is received in the appropriate context.

We appreciate the reviewer's comment, and we have now revised the first paragraph of the Main part.

"Natural selection and genetic drift produce differences in allele frequencies among populations, which could differentially shape phenotype distributions[6]. In humans, some phenotypic differences among genetic ancestry groups are known to have a genetic basis, including pigmentation[7], drug metabolism[8] and the prevalence of single-gene disorders[9,10]." (page 3, lines 66-73)
